# *Bifidobacterium* and *Lactobacillus* Probiotics and Gut Dysbiosis in Preterm Infants: The PRIMAL Randomized Clinical Trial

**DOI:** 10.1001/jamapediatrics.2024.2626

**Published:** 2024-08-05

**Authors:** Thea Van Rossum, Annette Haiß, Rebecca L. Knoll, Janina Marißen, Daniel Podlesny, Julia Pagel, Marina Bleskina, Maren Vens, Ingmar Fortmann, Bastian Siller, Isabell Ricklefs, Jonas Klopp, Katja Hilbert, Claudius Meyer, Roman Thielemann, Sybelle Goedicke-Fritz, Martin Kuntz, Christian Wieg, Norbert Teig, Thorsten Körner, Angela Kribs, Hannes Hudalla, Markus Knuf, Anja Stein, Christian Gille, Soyhan Bagci, Frank Dohle, Hans Proquitté, Dirk M. Olbertz, Esther Schmidt, Lutz Koch, Sabine Pirr, Jan Rupp, Juliane Spiegler, Matthias V. Kopp, Wolfgang Göpel, Egbert Herting, Sofia K. Forslund, Dorothee Viemann, Michael Zemlin, Peer Bork, Stephan Gehring, Inke R. König, Philipp Henneke, Christoph Härtel

**Affiliations:** 1 European Molecular Biology Laboratory, Heidelberg, Germany; 2 Department of Pediatrics, University Hospital Schleswig-Holstein, Campus Lübeck, Lübeck, Germany; 3 Department of Pediatrics, University Hospital Mainz, Mainz, Germany; 4 Experimental and Clinical Research Center, Max Delbrück Center for Molecular Medicine in the Helmholtz Association and Charité-Universitätsmedizin Berlin, Berlin, Germany; 5 Department of Pediatrics, University Hospital Würzburg, Würzburg, Germany; 6 Department of Pediatrics, University Hospital Hamburg-Eppendorf, Hamburg-Eppendorf, Germany; 7 Institute for Medical Biometry and Statistics, University of Lübeck, Lübeck, Germany; 8 Department of General Pediatrics and Neonatology, Saarland University Homburg, Germany; 9 Department of Pediatrics, University of Freiburg, Freiburg, Germany; 10 Children’s Hospital Aschaffenburg-Alzenau, Aschaffenburg, Germany; 11 Department of Pediatrics, University of Bochum, Bochum, Germany; 12 Children’s Hospital Mitte Bremen, Bremen, Germany; 13 Department of Pediatrics, University of Cologne, Cologne, Germany; 14 Department of Neonatology, University of Heidelberg, Heidelberg, Germany; 15 Children’s Hospital Horst-Schmidt-Kliniken Wiesbaden, Wiesbaden, Germany; 16 Children’s Hospital Worms, Worms, Germany; 17 Department of Pediatrics I, University of Duisburg-Essen, Duisburg-Essen, Germany; 18 Department of Neonatology, University of Tübingen, Tübingen, Germany; 19 Department of Neonatology, University of Bonn, Bonn, Germany; 20 Children’s Hospital Paderborn, Paderborn, Germany; 21 Department of Neonatology, University of Jena, Jena, Germany; 22 Department of Neonatology, Hospital Rostock Südstadt, University of Rostock, Rostock, Germany; 23 Helios Children’s Hospital Schwerin, Schwerin, Germany; 24 Children’s Hospital Hamburg Wilhelmstift and Marien-Hospital Hamburg, Medical School Hamburg, Hamburg, Germany; 25 Department of Neonatology, Allergology and Pediatric Pneumology, Hannover Medical School, Hannover, Germany; 26 Department of Infectious Diseases and Microbiology, University of Lübeck, Lübeck, Germany; 27 German Center of Infectious Diseases Research, Hamburg-Lübeck-Borstel-Riems, Hamburg, Germany; 28 Department of Pediatrics, University Hospital of Berne, Berne, Switzerland; 29 Center for Genderspecific Biology and Medicine, Saarland University Homburg, Homburg, Germany; 30 Center vor Digital Neurotechnologies Saar, Saarland University Homburg, Homburg, Germany; 31 Institute for Immunodeficiency, Centre for Chronic Immunodeficiency, University Medical Centre and Faculty of Medicine, University of Freiburg, Freiburg, Germany.; 32 Institute for Infection Prevention and Control, University Medical Centre and Faculty of Medicine, University of Freiburg, Freiburg, Germany

## Abstract

**Importance:**

The effects of probiotic interventions on colonization with resistant bacteria and early microbiome development in preterm infants remain to be clarified.

**Objective:**

To examine the efficacy of *Bifidobacterium longum* subsp *infantis*, *Bifidobacterium animalis* subsp *lactis* (BB-12), and *Lactobacillus acidophilus* (La-5) probiotics to prevent colonization with multidrug-resistant organisms or highly epidemic bacteria (MDRO+) and to shape the microbiome of preterm infants toward the eubiotic state of healthy full-term infants.

**Design, Setting, and Participants:**

The multicenter, double-blinded, placebo-controlled, group sequential, phase 3 Priming Immunity at the Beginning of Life (PRIMAL) randomized clinical trial, conducted from April 2018 to June 2020, included infants with gestational age of 28 to 32 weeks at 18 German neonatal units. Data analyses were conducted from March 2020 to August 2023.

**Intervention:**

A total of 28 days of multistrain probiotics diluted in human milk/formula starting within the first 72 hours of life.

**Main Outcomes and Measures:**

Colonization with MDRO+ at day 30 of life (primary end point), late-onset sepsis and severe gastrointestinal complication (safety end points), and gut dysbiosis, ie, deviations from the microbiome of healthy, term infants (eubiosis score) based on 16-subunit ribosomal RNA and metagenomic sequencing.

**Results:**

Among the 643 infants randomized until the stop of recruitment based on interim results, 618 (median [IQR] gestational age, 31.0 [29.7-32.1] weeks; 333 male [53.9%]; mean [SD] birth weight, 1502 [369] g) had follow-up at day 30. The interim analysis with all available data from 219 infants revealed MDRO+ colonization in 43 of 115 infants (37.4%) in the probiotics group and in 39 of 104 infants (37.5%) in the control group (adjusted risk ratio, 0.99; 95% CI, 0.54-1.81; *P* = .97). Safety outcomes were similar in both groups, ie, late-onset sepsis (probiotics group: 8 of 316 infants [2.5%]; control group: 12 of 322 infants [3.7%]) and severe gastrointestinal complications (probiotics group: 6 of 316 infants [1.9%]; control group: 7 of 322 infants [2.2%]). The probiotics group had higher eubiosis scores than the control group at the genus level (254 vs 258 infants; median scores, 0.47 vs 0.41; odds ratio [OR], 1.07; 95% CI, 1.02-1.13) and species level (96 vs 83 infants; median scores, 0.87 vs 0.59; OR, 1.28; 95% CI, 1.19-1.38). Environmental uptake of the *B infantis* probiotic strain in the control group was common (41 of 84 [49%]), which was highly variable across sites and particularly occurred in infants with a sibling who was treated with probiotics.

**Conclusions and Relevance:**

Multistrain probiotics did not reduce the incidence of MDRO+ colonization at day 30 of life in preterm infants but modulated their microbiome toward eubiosis.

**Trial Registration:**

German Clinical Trials Register: DRKS00013197

## Key Points

**Question:**

For preterm infants exposed to a variety of microbiome-disturbing factors, does administration of multistrain *Bifidobacteria* and *Lactobacillus* probiotics reduce the rate of colonization with multidrug-resistant organisms and highly epidemic bacteria (MDRO+) at day 30 of life compared with placebo?

**Findings:**

In this large-scale, phase 3, randomized clinical trial targeting MDRO+ colonization in 618 preterm infants at 28 to 32 weeks’ gestation, MDRO+ colonization occurred in 37.4% receiving probiotics compared with 37.5% receiving placebo. Probiotic treatment modulated the microbiome composition toward eubiosis patterns typical for healthy full-term infants, and the *B infantis* probiotic strain had a low threshold for environmental acquisition.

**Meaning:**

In preterm infants at high risk for dysbiosis, multistrain probiotics did not lead to a reduction in colonization with MDRO+ pathogens at day 30 of life; these findings on environmental uptake of probiotic strains in infants treated with placebo contribute to a better understanding of endemic flora dynamics in neonatal care units.

## Teaser

The Priming Immunity at the Beginning of Life (PRIMAL) randomized clinical trial investigates if preterm infant exposure to multistrain *Bifidobacteria* and *Lactobacillus* probiotics reduces the colonization rate of multidrug-resistant organisms at day 30 of life compared with placebo.

## Introduction

Preterm infants face numerous challenges that can perturb their developing microbiomes including exposure to multidrug-resistant organisms (MDRO), a problem that is increasing. It is, therefore, not surprising that their microbiome establishment significantly deviates from the physiological trajectories observed in term infants born vaginally, fully breastfed, and not exposed to antibiotics.^1,2^ Key features that commonly distinguish these dysbiotic states in preterm infants from the eubiosis patterns in their term counterparts include reduced diversity, a scarcity of *Bifidobacteria*, increased abundance of pathobionts with MDRO features and/or epidemic potential (hereafter collectively referred to as MDRO+), and functional deficiencies, eg, in metabolizing human milk oligosaccharides (HMOs).^3^ Although the microbiomes of many preterm infants eventually recover and realign toward eubiosis, the specific factors that drive this shift remain elusive.^1^ Dysbiosis early in life can result in long-term health consequences, such as altered immune development.^2,3,4^ In cases where the microbiome further derails, infants are at an increased risk of serious complications, including bloodstream infections,^5^ the need for reserve antibiotics, and transmission of MDRO+.^6,7^ The bloom of MDRO+ in the preterm gut has also been linked to the development of necrotizing enterocolitis (NEC)^8^ and brain damage.^9^ Dysbiosis leading to disease may be characterized as a failure of the microbiome to prevent MDRO+ from negatively impacting health, either through deficient MDRO+ colonization resistance or failure to control MDRO+ population growth. As a clinical consequence, infants colonized with MDRO+ are often cared for with extended barrier precautions including isolation rooms, which may impact patient safety and neurodevelopment.

Probiotics are live bacteria that, when administered in adequate amounts, hold promise for targeting dysbiosis in preterm infants, as they excel as gut colonizers of vaginally born, breast milk–fed infants.^1,2,10,11^ Although the European Society for Paediatric Gastroenterology Hepatology and Nutrition guidelines recommend the use of multistrain probiotics to reduce the incidence of NEC,^12^ the American Academy of Pediatrics does not support routine administration of probiotics to preterm infants based on the rationale that dietary supplement–grade probiotics have recently been recalled due to contamination.^13^ Little is known about the capacity of probiotics to normalize the nascent microbiome using cutting-edge methodology.^1,11,14^ The Priming Immunity at the Beginning of Life (PRIMAL) randomized clinical trial tested the hypotheses that multistrain probiotics containing *Bifidobacterium longum* subsp *infantis*, *B animalis* subsp *lactis* (BB-12), and *Lactobacillus acidophilus* (La-5) would prevent dysbiosis by reducing colonization with MDRO+ or shaping the microbiome in preterm infants (born at 28 to 32 weeks of gestation) toward term infant patterns.

## Methods

### Study Design

The PRIMAL study was an investigator-initiated, multicenter, double-blinded, placebo-controlled, group-sequential, randomized clinical trial conducted in 18 tertiary neonatal intensive care units (NICUs) in Germany. The study protocol was previously published^11^ and is described in detail in Supplement 1 (statistical analysis plan available in Supplement 2). In brief, infants were considered for enrollment within the first 48 hours of life if they were (1) born at a study center and (2) within the gestational age range of 28 weeks 0 days and 32 weeks 6 days. After written informed consent, participants were block randomized to probiotics (verum) or placebo in a 1:1 ratio within 48 hours after birth. Multiple births were randomized independently. Maternal race and ethnicity were identified by asking for citizenship and first language spoken in the family; the following races and ethnicities were included: Asian; Middle East, North Africa, or Turkey; other African, and White. The PRIMAL trial was approved by the institutional review board of all participating sites. The EMMA (Impact of Mother’s Own Milk on the Development of Allergy and Airway Infections) study was approved by the institutional review board of the University of Lübeck. The study was conducted according to current Consolidated Standards of Reporting Trials (CONSORT) reporting guidelines and Standard Protocol Items: Recommendations for Interventional Trials (SPIRIT) reporting guidelines.

### Intervention

Verum and placebo were provided in daily dose capsules of identical appearance and administered over 28 days. Probiotics were taken from a single batch of the probiotic mixture consisting of *B longum* subsp *infantis*, BB-12, and La-5, at a dose of 1.5 × 10^9^ colony-forming units of each strain per capsule. Placebo was cornstarch powder of similar color and odor as verum. Probiotic bacteria in verum powder were analyzed by whole-genome DNA sequencing as described in eTable 1 in Supplement 3.

### Data Collection and Sampling

Clinical metadata were documented in case report forms at day 3, day 30, and at discharge. Follow-up assessments were performed at age 6, 12, and 24 months and will be reported elsewhere.^11^ Stool samples were collected on day 3 and day 30 while timing varied in the study population, ie, day 3 (median [IQR], 3 [2-4] days of life), day 30 (median [IQR], 30 [28-31] days of life). From each stool sample, 3 aliquots were taken for (1) local MDRO+ screening by the study site microbiology laboratory, (2) central MDRO+ screening, and (3) microbiome analysis (eFigure 1 in Supplement 3).

### Outcomes

The primary efficacy end point was defined as colonization with MDRO+ based on local, site-specific microbiological screening for MDRO+ at day 30 (eAppendix 1 in Supplement 3) as these data have an ad hoc impact on the clinical management, eg, choice of empirical antibiotics or extended barrier precautions. We thereby acknowledged the heterogeneity of screening approaches across sites regarding selection of pathogens, culture media, and diagnostic methods. Predefined safety outcomes were late-onset sepsis,^7^ severe gastrointestinal complication, or death. Secondary outcomes were weight gain at 30 days of life and at discharge and MDRO+ status assessed by a standardized MDRO+ surveillance of fecal samples shipped to our core facility in Mainz (eTable 2 in Supplement 3). We also explored the efficacy of probiotics to prevent dysbiosis on genus and species level (eAppendix 1 in Supplement 3). Adverse events were defined according to Common Terminology Criteria for Adverse Events and ascertained for potential relatedness to the intervention.

### Microbiome Composition Analysis

16-Subunit (S) ribosomal RNA gene sequencing was performed as recently described in a study by Klopp et al.^15^ Deep whole-genome metagenomic sequencing (metaG data) was performed on the Illumina HiSeq 4000 platform (Illumina). Strain-resolved detection results for the verum strains were generated using the metaG data, using the metagenomic single-nucleotide variants (metaSNVs)-based ProTection tool^16^ and SameStr^17^ (eAppendix 3 and eTables 6-7 in Supplement 3).

### Eubiosis Score Modeling

Eubiosis modeling was created by using published metagenomes to distinguish between preterm microbiomes (339 metagenomes of probiotic-naive infants from 5 studies) and healthy full-term microbiomes (153 metagenomes from 7 studies) and cross-validated in a training set (eAppendix 4, eTable 8, and eFigure 2 in Supplement 3). For further evaluation of eubiosis, we enrolled a single-center cohort of term, healthy, exclusively breastfed infants with fecal sampling on day 30 in the EMMA study.

### Statistical Analysis

The study aimed to recruit 654 infants, providing 80% power using the 1-sided α = .02 test level (continuity-corrected χ^2^) to detect an absolute risk reduction of 7.5% (relative risk reduction of 50%) in the incidence of MDRO+ positivity, which for the control was projected to be 15%.^11^ A group sequential plan was used with interim analysis at 50% information time (n = 322 infants), a 1-sided α = .01 at the interim, and a futility stop of α0 = 0.7.^11^ The primary end point was analyzed using the intention-to-treat set as randomized with a generalized linear mixed-effects model including sex and gestational age as fixed factors and study site as random effects for both, interim, and final analysis. Exploratory outcomes were analyzed in the as-treated population. Statistical analysis of microbiome sequencing is described in eAppendix 2 and eTables 3 to 5 in Supplement 3. All *P* values were 2-sided, and *P* <.05 was considered significant. Data analyses were conducted from March 2020 to August 2023 using SAS, version 9.4 (SAS Institute); R, version 3.5.1 to 4.3.1 (R Foundation); and addPlan10 (Berry Consultants). Other data analyses software used are listed in eTable 5 in Supplement 3.

## Results

### Study Infants

**Figure 1.**
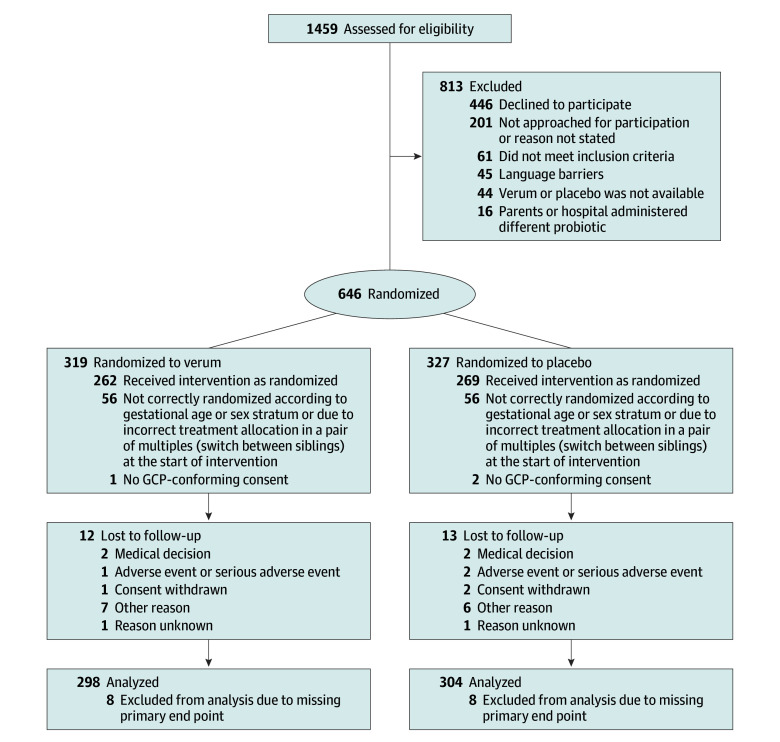
Enrollment, Randomization, and Follow-Up in the Priming Immunity at the Beginning of Life (PRIMAL) Randomized Clinical Trial The study flow diagram describes the study design. The lack of adherence to block randomization toward the end of the enrollment period was mainly related to shortage of boxes for intervention for specific strata. The boxes had been prepacked by the study pharmacy, shipped to the sites in 1 load, and stored on site until use. Site investigators had chosen remaining boxes from different gestational age or sex strata than set by the study protocol in 110 infants (18%). One sibling pair was mixed and did not receive the allocated intervention. GCP indicates Good Clinical Practice.

Participants were enrolled from April 14, 2018, to June 10, 2020. Patient follow-up to hospital discharge was completed on July 31, 2020. Until the end of recruitment based on interim results, a total of 1459 infants were screened, 646 were randomized, 643 were allocated, and 618 infants (median [IQR] gestational age, 31.0 [29.7-32.1] wk; 285 female [46.1%]; 333 male [53.9%]) had follow-up at day 30 (Figure 1). The following maternal races and ethnicities were identified: 9 Asian (1.5%), 35 Middle East, North Africa, or Turkey (5.7%), 14 other African (2.3%), and 540 White (90.3%). A total of 99 term, healthy, exclusively breastfed infants with fecal sampling on day 30 were enrolled in the EMMA study (eAppendix 5 in Supplement 3). The mean (SD) birth weight was 1502 (369) g. Cesarean delivery was the predominant mode of delivery (497 of 618 [80.4%]), and 396 of 618 infants (64.2%) received postnatal antibiotics. The PRIMAL trial intervention was commenced on day 2 (median [IQR], 1 [1-2] days of life) and stopped at day 28 (median [IQR], 26 [26-30] days of life). Baseline characteristics of the study infants (Table 1), maternal characteristics (eTable 9 in Supplement 3), and treatments and continuous outcomes (eTable 10 in Supplement 3) were similar between groups. The number of enrolled infants per site is given in eTable 16 in Supplement 3.

**Table 1.** Baseline Characteristics of Study Population^a^

Characteristic	All No./total No.	All (N = 618)	Verum No./total No.	Verum (n = 306)	Control No./total No.	Control (n = 312)
Gestational age, median (IQR), wk	618/618	31.0 (29.7 to 32.1)	306/306	30.93 (29.9 to 32.0)	312/312	31 (29.5 to 32.14)
Birth weight, mean (SD), g	618/618	1502.2 (369.1)	306/306	1504.7 (360.0)	312/312	1499.8 (378.4)
z Score of birth weight^b^	618/618	0.03 (−0.65 to 0.45)	306/306	0.05 (−0.62 to 0.45)	312/312	0.01 (−0.67 to 0.44)
Female sex	618/618	285 (46.1)	306/306	140 (45.8)	312/312	145 (46.5)
Male sex	618/618	333 (53.9)	306/306	166 (54.2)	312/312	167 (53.5)
Multiple birth	618/618	266 (43.0)	306/306	135 (44.1)	312/312	131 (42.0)
Body length, cm	615/618	40.6 (3.3)	305/306	40.8 (3.5)	310/312	40.48 (3.2)
Body head circumference, cm	617/618	28.4 (2.0)	305/306	28.48 (2.0)	312/312	28.36 (2.0)
Peripartum data						
Mode of birth						
Spontaneous	618/618	121 (19.6)	306/306	61 (20.0)	312/312	60 (19.2)
Cesarean delivery, elective	618/618	431 (69.7)	306/306	211 (69.0)	312/312	220 (70.5)
Cesarean delivery, emergency	618/618	66 (10.7)	306/306	34 (11.1)	312/312	32 (10.3)
Cause of preterm birth						
Preterm labor	618/618	294 (47.6)	306/306	148 (48.4)	312/312	146 (46.8)
Premature rupture of membranes	618/618	30 (4.9)	306/306	12 (3.9)	312/312	18 (5.8)
Amniotic infection syndrome	618/618	62 (10.0)	306/306	31 (10.1)	312/312	31 (9.9)
Preeclampsia	618/618	44 (7.1)	306/306	24 (7.8)	312/312	20 (6.4)
HELPP syndrome	618/618	36 (5.8)	306/306	19 (6.2)	312/312	17 (5.5)
Growth restriction	618/618	89 (14.4)	306/306	45 (14.7)	312/312	44 (14.1)
Placental abruption	618/618	30 (4.9)	306/306	14 (4.6)	312/312	16 (5.1)
Pathological cardiotocography	618/618	112 (18.1)	306/306	55 (18.0)	312/312	57 (18.3)
Prolapse of membranes	618/618	16 (2.6)	306/306	10 (3.3)	312/312	6 (1.9)
Rupture of membranes without anhydramnios	618/618	105 (17.0)	306/306	58 (19.0)	312/312	47 (15.1)
Other	618/618	121 (19.6)	306/306	60 (19.6)	312/312	61 (19.6)
Antenatal steroids administered	617/618	536 (86.9)	305/306	261 (85.6)	312/312	275 (88.1)
If yes completed cycle (2 doses, 12 h after 2 dose)	531/618	405 (76.3)	259/306	195 (75.3)	272/312	210 (77.2)

Abbreviation: HELPP, hemolysis, elevated liver enzymes, and low platelets.

^a^ Table 1 describes the clinical baseline data of the study population (intention-to-treat population).

^b^ Fenton growth chart.

### Primary Outcome

At the time point of planned interim analysis, 662 infants were screened, 322 infants randomized, and 219 infants analyzed (eTable 11 in Supplement 3). The reasons for exclusion of infants at this time point are given in eFigure 3 in Supplement 3, which were mostly due to the postponement in query responses by study sites and further lockdown restrictions during the COVID-19 pandemic. The primary end point was observed in 43 of 115 infants (37.4%) in the verum group and 39 of 104 infants (37.5%) in the control group. The adjusted risk difference between groups was 1.3%, and the relative risk was 0.99 (95% CI, 0.54-1.81; *P* = .97). Further recruitment was stopped, all infants already enrolled were followed up as planned, and data were analyzed as exploratory outcomes. In the total study population (n = 602), MDRO+ colonization at day 30 was positive in 103 of 298 infants (34.6%) in the verum group vs 115 of 304 infants (37.8%) in the control group (odds ratio [OR], 0.87; 95% CI, 0.59-1.28), respectively (Table 2).

**Table 2.** Efficacy and Safety Outcomes^a^

Outcome	Total No.	Verum	Control	Adjusted RR or OR (95% CI)^b^
Primary outcome				
Local screening, MDRO+ colonization day 30, interim analysis, No./total No. (%)^e^	219	43/115 (37.4)	39/104 (37.5)	0.99 (0.54-1.81)^c^^,^^d^
Secondary outcomes				
Local screening, MDRO+ colonization day 30, all infants, No./total No. (%)	602	103/298 (34.6)	115/304 (37.8)	0.87 (0.59-1.28)^f^
Central screening, MDRO+ colonization day 30, No./total No. (%)	544	132/272 (48.5)	128/272 (47.1)	1.07 (0.76-1.5)
Eubiosis classification at bacterial genus resolution, No./total No. (%)	512	141/254 (55.9)	114/258 (44.2)	1.61 (1.12-2.31)
Eubiosis model score at bacterial genus resolution, No.; median (IQR)	512	254; 0.47 (0.31-0.67)	258; 0.41 (0.14-0.68)	1.07 (1.02-1.13)
Eubiosis classification at bacterial species resolution, No./total No. (%)	179	60/96 (62.5)	29/83 (34.9)	3.19 (1.71-5.96)
Eubiosis model score at bacterial species resolution, No.; median (IQR)	179	96; 0.87 (0.72-0.99)	83; 0.59 (0.35-0.81)	1.28 (1.19-1.38)
**Safety outcomes, No.**	638	316	322	
Blood culture–proven sepsis all, No. (%)^g^	28 (4.4)	14 (4.4)	14 (4.3)	NA
Early onset^h^	8 (1.3)	6 (1.9)	2 (0.6)	
Late onset	20 (3.1)	8 (2.5)	12 (3.7)	
Clinical sepsis all, No. (%)	49 (7.7)	26 (8.2)	23 (7.1)	
Early onset^i^	38 (6.0)	20 (6.3)	18 (5.6)	
Late onset	11 (4.2)	6 (1.9)	5 (1.6)	
NEC ≥Bell stage 2, No. (%)	1 (0.2)	NA	1 (0.3)^i^	
Focal intestinal perforation, No. (%)	2 (0.4)	1 (0.3)	1 (0.3)	
Other severe gastrointestinal complication, No. (%)^j^	10 (1.6)	5 (1.6)	5 (1.6)	
Death, No. (%)	1 (0.2)	1 (0.3)^k^	NA	
Gestational age at day 30, median (IQR), wk	35.0 (33.7 to 35.9)	35.0 (33.9 to 35.9)	35.0 (33.7 to 35.9)	
z Score of weight Fenton at day 30, median (IQR)^l^	−1.24 (−1.72 to −0.77)	−1.17 (−1.73)	−1.28 (−1.75 to −0.83)	
Gestational age at discharge, median (IQR), wk^l^	36.6 (35.9 to 37.9)	36.71 (35.9 to 37.7)	36.57 (35.9 to 37.9)	
z Score of discharge weight Fenton, median (IQR)^k^	−1.21 (−1.68 to −0.76)	−1.17 (−1.62 to −0.69)	−1.25 (−1.75 to −0.82)	

Abbreviations: aRR, adjusted risk ratio; GLM, generalized linear mixed-effects model; ITT, intention to treat; MDRO, multidrug-resistant organisms and highly epidemic bacteria; NA, not applicable; NEC, necrotizing enterocolitis; OR, odds ratio; RR, relative risk; SA, safety analysis.

^a^ Table 2 describes the primary outcome (ITT population) and the secondary efficacy outcomes (as-treated population) and the predefined clinical safety outcomes (SA population) of the PRIMAL trial. The primary end point of MDRO+ gut dysbiosis was analyzed using the ITT in the interim population (eFigure 3 in Supplement 3) set as randomized with a GLM with sex and gestational age as a fixed factor, and study site as random effect. To test the treatment effect, a Wald test for the log OR was used, and corresponding (1 − α) CIs. Secondary outcomes were analyzed in the as-treated analysis sets and regarded as explorative. Safety end points were analyzed in the SA set.

^b^ Estimated in a generalized linear mixed model adjusted for site, sex, and age,

^c^ Reported from the ITT population.

^d^ Further monitoring revealed that data originally analyzed at interim had to be curated including verum or placebo group allocation.

^e^ *P* = .97.

^f^ Alternative model including family effect (multiple sibling): aRR 0.87 (95% CI, 0.59-1.30).

^g^ Pathogens identified in blood-culture: verum group, EOS: 2× *Streptococcus mitis*, *Escherichia coli*, *Staphylococcus aureus*, *Enterococcus faecalis*, 1× unknown; LOS: *S aureus*, MRSA, 2× *S haemolyticus*, 4× *Klebsiella pneumoniae*; placebo group: EOS *S aureus*, group B streptococci; LOS: *Enterobacter cloacae*, *S haemolyticus*, *S aureus*, 5× *S epidermidis*, *Serratia marcescens*, 2× group B streptococci, *K pneumoniae*.

^h^ A total of 43 of 46 EOS cases occurred before the start of intervention or at the day of intervention, 4 control infants with clinical EOS had environmental uptake of *B infantis* probiotics.

^i^ NEC: the infant had a congenital heart defect (aortic isthmus stenosis) as a risk factor.

^j^ Severe gastrointestinal complication: meconium plug syndrome (n = 5), gastric perforation (n = 2 including 1 surgical complication after pylorus hypertrophy), volvulus (n = 1), hematochezia (n = 2).

^k^ Death: the infant died at the age of 6 months due to severe congenital kidney disease.

^l^ z Scores day 30 available data from 607 infants; z scores at discharge available data from 616 infants.

### Secondary Outcomes

Standardized (central) assessment of MDRO+ at day 30 was performed in 544 infants and revealed no differences between the verum group (132 of 272 [48.5%]) and the control group (128 of 273 [46.9%]) but did identify a discrepancy between local screening data in 37% (χ^2^ = 34.7; Pearson χ^2^ with Yates continuity correction). The prevalence of MDRO+ among the 2 treatment groups varied between hospitals, but there was no difference between intervention groups (eFigure 4 in Supplement 3). Safety outcomes were similar between verum and control groups, including 20 infants diagnosed with culture-proven late-onset sepsis (8 of 316 [2.5%] in the verum group and 12 of 322 [3.7%] in the control group) and severe gastrointestinal complications (6 of 316 [1.9%] in the verum group and 7 of 322 [2.2%] in the control group) (Table 2). There was no case of sepsis with a probiotic strain. We observed 1 single NEC case in the control group and 1 death in the verum group caused by congenital kidney failure. A total of 197 adverse events were reported (eTable 12 in Supplement 3), ie, 115 of 316 infants (36.4%) in the verum group and 82 of 322 infants (25.5%) in the control group. A potential association with study participation was declared for 10 of 197 events (5.1%), ie, 9 late-onset sepsis and 1 NEC case. Full recovery was documented in 163 of 197 events (82.7%).

### Probiotics and the Establishment of a Microbiome

**Figure 2.**
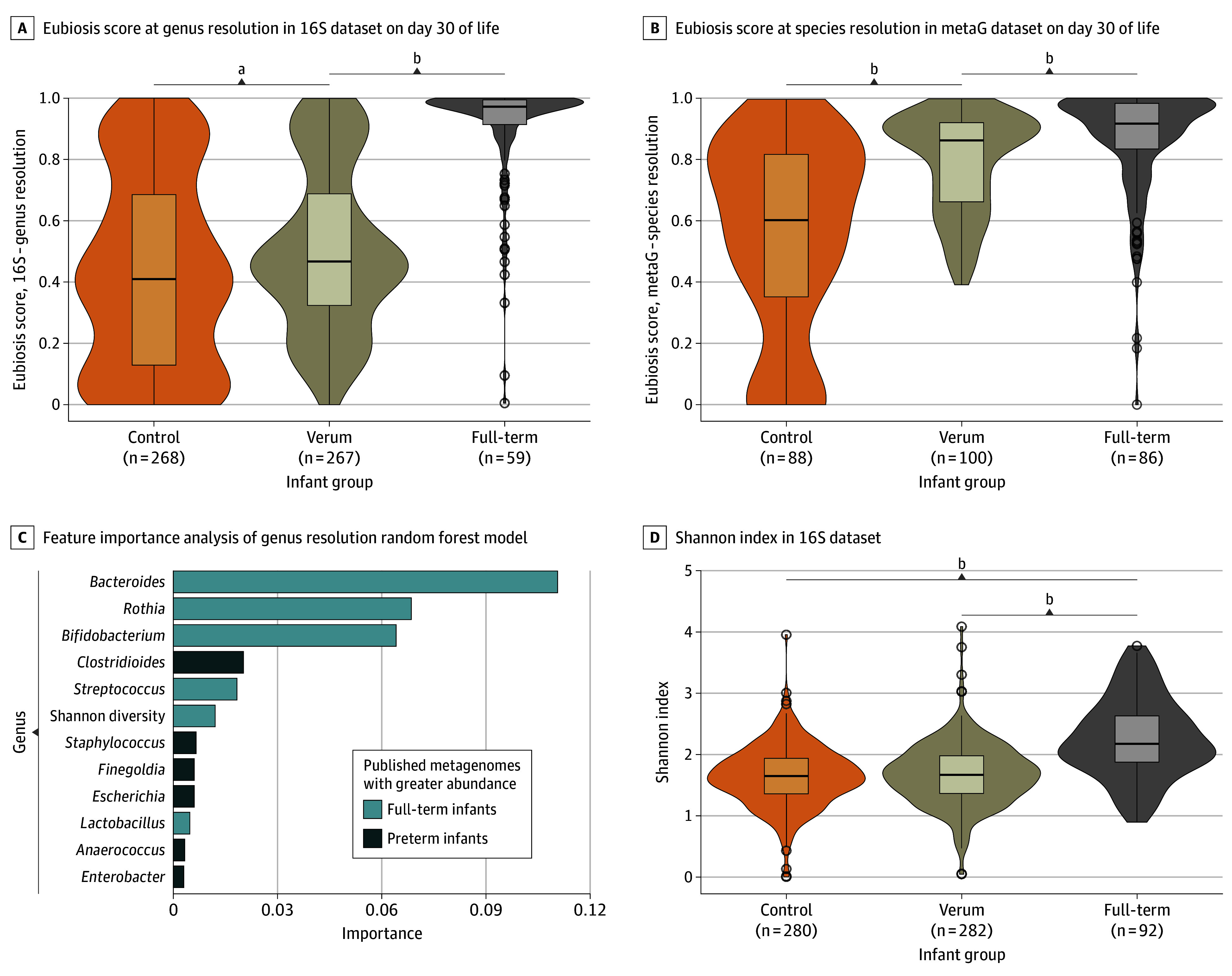
Probiotics, the Preterm Microbiome, and the Eubiotic State Typical of Full-Term Infants Eubiosis modeling was created for the first time by using published metagenomes to distinguish between preterm microbiomes (339 metagenomes of probiotic-naive infants from 5 studies) and healthy full-term microbiomes (153 metagenomes from 7 studies). The model produces a eubiosis score from 0 to 1, ie, higher values reflect the probability of a eubiotic (healthy) state and, therefore, less likely to be dysbiotic. At both species and genus level, this model distinguished preterm infant microbiomes from healthy, full-term microbiomes, with an accuracy of greater than 90% and an area under the curve of greater than 0.93 under cross-validation grouped by study. To assess whether differences in eubiosis scores were due to the presence of the probiotic bacteria themselves or due to other community composition differences, a second set of models was trained. Here, the modeling was repeated, but the probiotic taxa (*Bifidobacterium longum*, *Bifidobacterium animalis*, and *Lactobacillus acidophilus*) were excluded from model building. The resulting species and genus models distinguish appropriately between the preterm and full-term groups (accuracy >88%; area under the curve >0.92). The most important taxa for these models are the same as the previous models, without *Bifidobacterium*. These models were then also applied to the PRIMAL day 30 fecal microbiomes. A, Eubiosis score at genus resolution in the 16-subunit (S) dataset, compared among verum, control, and the healthy term EMMA (Impact of Mother’s Own Milk on the Development of Allergy and Airway Infections) trial cohort samples on day 30 of life. Infants in the verum group had higher eubiosis scores than infants in the control group but did not reach the maturation level of exclusively breastfed term infants at day 30. B, Eubiosis score at species resolution in the metagenomic (metaG) dataset, compared among verum, control, and the healthy term EMMA cohort samples on day 30 of life. C, Feature importance analysis of the genus resolution random forest model. Bar chart displaying the 12 most important features used by the random forest model to calculate the eubiosis score. The model was trained on published metagenomes from preterm and healthy term infants. The x-axis represents the feature importance. The color shades indicate whether the genus reported was more abundant in term infants or preterm infants in the published metagenomic datasets. Shannon diversity of those samples also contributed to the prediction score, with higher Shannon index found in healthy term infants. D, Alpha diversity, measured by the Shannon Index in the 16S dataset, compared among the verum, control, and the healthy groups, exclusively breastfed term infants (EMMA cohort samples on day 30 of life). ^a^Wilcoxon test significance denoted as (false discovery rate = 0.001). ^b^Wilcoxon test significance denoted as (false discovery rate = 2.2 × 10^−16^), explorative values.

The administration of probiotics resulted in an overall shift of the microbiome toward a state more similar to that of healthy full-term infants. This was illustrated by higher eubiosis scores in the verum group as compared with the control group at the genus level (254 vs 258 infants; median [IQR], 0.47 [0.31-0.67] vs 0.41 [0.14-0.68] scores; OR, 1.07; 95% CI, 1.02-1.13) and species level (96 vs 83 infants; median [IQR], 0.87 [0.72-0.99] vs 0.59 [0.35-0.81] scores; OR, 1.28; 95% CI, 1.19-1.38) (Figure 2A-C; Table 2). Alpha diversity was not directly affected by the intervention (Figure 2D).

### Environmental Uptake of Probiotics

**Figure 3.**
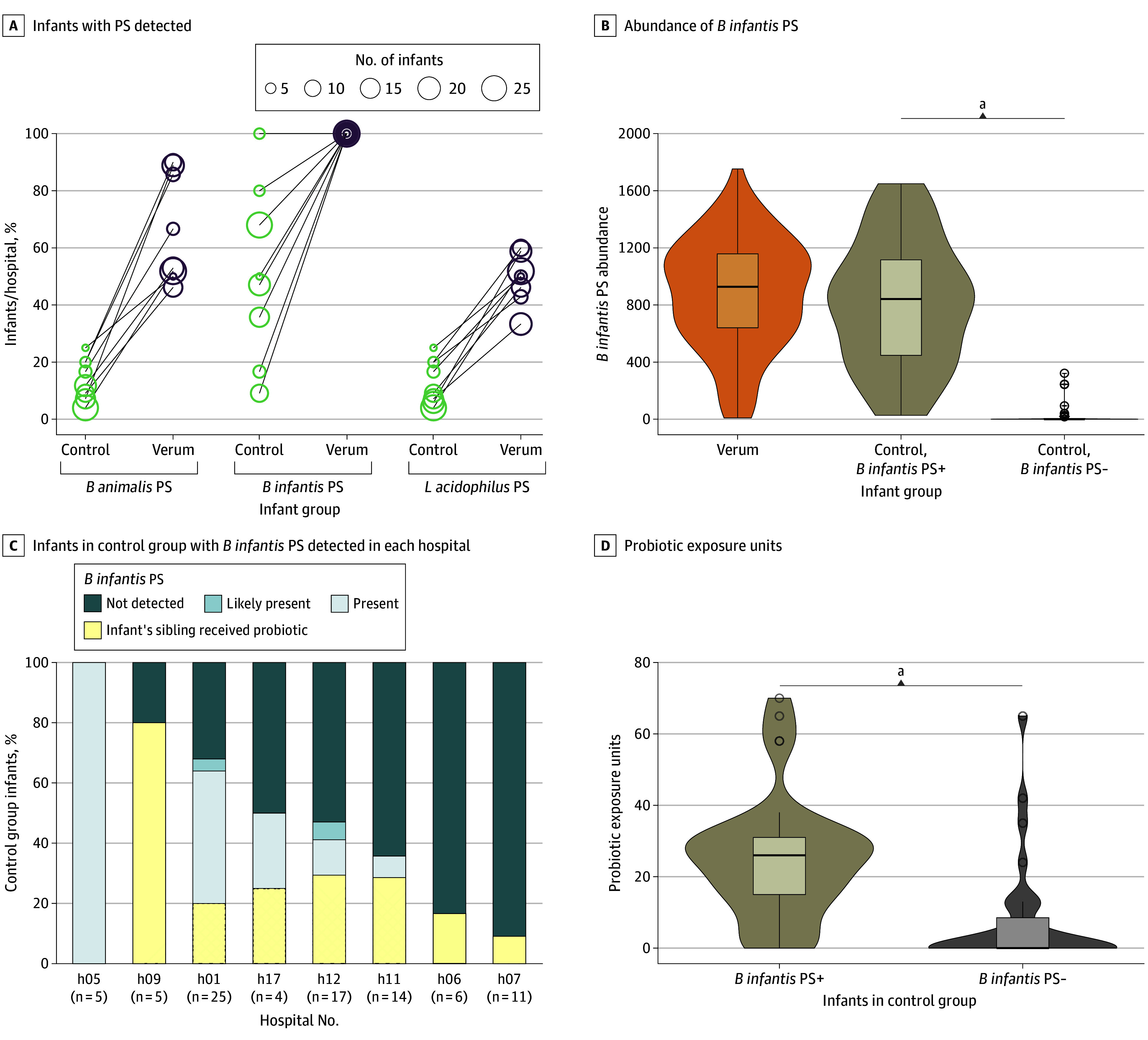
Analysis of Probiotic Uptake via Microbiome Profiling Demonstrating Environmental Acquisition of Probiotic *Bifidobacterium infantis* Strain Environmental uptake of probiotic *B infantis* strain in placebo infants mainly related to multiple birth (verum-treated sibling) and hospital site. Cross-colonization of control infants with *Bifidobacteria* has been suggested in the PIPS (Probiotics in Preterm Infants) trial without provision of firm evidence for this.^18^ We analyzed a subset sample undergoing metagenomic sequencing (n = 184) and found probiotic bacteria more prevalent in the verum group than in the placebo group. The abundance of the *B infantis *probiotic strain (PS), when present, was not significantly different between the verum and control group (Wilcoxon *P* = .20). *B animalis* and *Lactobacillus acidophilus* were observed in 65% and 49% of the verum group samples, respectively, and were rarely observed in the control group (<5%) (eTable 13 in Supplement 3). All 3 probiotic strains were observed in 43 of 100 infants in the verum group and 2 of 84 infants in the control group. We next addressed potential causes of *B infantis* PS environmental uptake in control group infants, which varied greatly across hospitals (10%-100%). A verum-treated sibling (twin or triplet) caused environmental uptake in 21 of 23 control siblings (90%). Exposure units were 3 times higher for *B infantis *PS-positive control infants than for PS-negative control infants (mean [SD], 25.5 [11.4] vs 7.9 [10.5] units). A, Percentage of infants per hospital wherein the PS were detected, compared between treatment groups. Each hospital is indicated by a circle, where green represents the control group, purple represents the verum group, and the size of the circle indicates the number of infants per hospital in each group. B, Abundance of *B infantis* PS between the verum and control groups. We performed high-resolution (single-nucleotide variant–level) detection of PS and estimated differences in the abundance between the groups using the Wilcoxon rank sum test. The control group is further divided into infants where the *B infantis* PS was detected (control *B infantis* PS+) and infants where it was not detected (control *B infantis* PS−). Abundance is measured as the mean depth of coverage of reads mapped to the reference genome divided by the total number of reads per sample normalized by z score. C, Each horizontal bar represents the percentage of control-group infants with *B infantis* PS detected in each hospital. Only the 8 hospital sites with infants having metagenomic sequenced samples are shown. Colors indicate whether *B infantis* PS was detected or not. The yellow bar displays the percentage of *B infantis* PS+ infants who had a sibling in the verum group. D, Comparison of probiotic exposure units between the control group infants with *B infantis* PS detected (control *B infantis* PS+) and those where it was not detected (control *B infantis* PS−). ^a^Wilcoxon test *P* < .001, explorative values.

Cross-colonization of control infants with *Bifidobacteria* has been suggested in the PIPS (Probiotics in Preterm Infants) trial without firm evidence for this at the species level.^18^ We therefore analyzed a subset of samples undergoing metagenomic sequencing (n = 184) and found probiotic bacteria more prevalent in the verum group than in the control group (Figure 3A). The scale of these differences varied across the 3 probiotic strains (PS) (eTable 13 in Supplement 3). Specifically, the PS of *B infantis* was detected in all verum infants (n = 100) and in 49% of the control infants (41 of 84) after the treatment period. The abundance of *B infantis* PS, when present, did not differ between groups (Figure 3B). BB-12 and La-5 were observed in 65% (65 of 100) and 49% (49 of 100) in the verum group, respectively, and rarely observed in controls (<5%) (eTable 13 in Supplement 3). Acquisition of *B infantis* PS in placebo infants varied greatly across hospitals (10%-100%) (Figure 3C) and was related to the extent of exposures to probiotic treatment in the infant’s environment (exposure units = days of proximity to an infant treated with verum) (Figure 3D and eTable 14 in Supplement 3). Specifically, 21 of 23 multiples (ie, 22 pairs of twins, 1 set of triplets) treated with placebo (90%) acquired *B infantis* PS from their verum-treated sibling. In the 16S dataset, we also noted a higher abundance of the subgenus group that contains *B infantis* probiotic (*Bifidobacterium* ASV3) in the control with verum sibling as compared with singletons treated with placebo (eAppendix 6 and eFigure 5A in Supplement 3). The microbiome variance was explained to 5% by the PRIMAL trial intervention and 6% by hospital (eFigure 5D in Supplement 3), whereas type of feeding or antibiotics had an impact of less than 2%. *Bifidobacterium* ASV3 abundance negatively correlated with *Clostridium sensu stricto 1*,* Klebsiella*, and* Enterococcus* genus (eFigure 6 and eAppendix 7 in Supplement 3).

### Eubiosis Shift and the Presence of *B infantis* PS

An exploratory analysis stratified to the presence of probiotic *B infantis* in 179 infants showed that infants who were positive for *B infantis* had no benefits in terms of MDRO+ colonization but had higher eubiosis scores (eTable 15 in Supplement 3). When in our model—based on published metagenomes of infants without exposure to probiotics—the multistrain probiotic species administered in the PRIMAL trial were not used for eubiosis prediction, a stringent distinction between typical full-term and preterm infants’ microbiome composition was possible. However, when this model was applied to infants in the PRIMAL trial, there was no difference according to treatment group or to probiotic presence (Kruskal-Wallis and Wilcoxon rank sum tests, *P* > .5). This indicates that the treatment-associated shift toward a more eubiotic state was driven by the presence of the probiotic bacteria themselves, especially strain *B infantis*, and not by broader community effects. Infants who were positive for *B infantis* also had higher carriage of HMO-metabolizing genes and lower abundance of pathobionts (eTable 15 in Supplement 3).

## Discussion

In the PRIMAL randomized clinical trial, administration of *B infantis, *BB-12, and La-5 compared with placebo did not significantly reduce the rate of MDRO+ colonization in preterm infants at high risk for gut dysbiosis. Furthermore, probiotic treatment remained without significant effect on clinical and safety outcomes such as sepsis, gastrointestinal complications, and weight gain. Probiotics promoted a microbiome pattern converging toward the taxonomic composition typical for healthy term infants with *B infantis* being the main driver of eubiosis or normative gut microbiome maturation. Intriguingly, metagenomic sequencing revealed the substantial presence of probiotic bacteria in 49% of infants treated with placebo, a phenomenon we presume to be due to environmental uptake. This uptake particularly occurred in infants with a verum-treated sibling, yet it was also found with notable frequency in placebo-treated singletons. Because *B infantis* was by far the most frequently acquired probiotic strain, it can be speculated that interindividual microbiome barriers are particularly low for this species. This finding implies different transmission rates for pathogens, which is important for understanding the dynamics of endemic flora in neonatal units. We found no effect on MDRO+ colonization based on probiotic *B infantis* colonization status.

The global challenge posed by MDRO+ as pathogens in neonatal infections highlights the urgent need for interventions.^6,19^ Although active surveillance through MDRO+ culture screening has been implemented in German NICUs, partly in response to increasing public media attention on MDRO+ outbreaks, the predictive value of routine screening on MDRO+ sepsis prevention remains debatable.^11,20^ We used the time point day 30 as assessment for MDRO+ because previous data had shown that colonization of preterm infants with MDRO usually occurs within the neonatal period,^21^ which is also the most vulnerable time frame for the development of dysbiosis-related disease such as sepsis.^11^ Current strategies to bolster host resistance against MDRO+ colonization are limited. Probiotics have MDRO+ preventive potential as they can produce bacteriocins against Enterobacteriaceae, stabilize mucosal barriers, and compete for intestinal adherence through their metabolizing capacity for HMOs.^22,23^ A randomized clinical trial^24^ in 60 term infants demonstrated a decreased intestinal carriage of antimicrobial resistance genes in the *B infantis* group. This finding was not confirmed in the PRIMAL trial, which may reflect differences in study design, gestational age, and timing of intervention, as earliest possible engraftment of probiotic strains into the nascent microbial ecosystem may enhance the competitive advantage against MDRO.^14^ We therefore postulate that MDRO+ surveillance is helpful for prompt identification of nosocomial transmission and guidance of antimicrobial use^6,11^; however, in the context of preventing dysbiosis at the ecosystem level, the binary end point of MDRO+ falls short as sufficient measure to evaluate efficacy.

A unique hallmark of this multicenter trial is assessment of microbiome composition at high resolution. Several key findings emerged. *B infantis* confirmingly demonstrated a high capacity to colonize the infant gut.^1,25^ This colonization, in turn, is suggested to establish a state of eubiosis, mirroring the microbiome profile typically found in term infants. This finding underlines the pivotal role of *B infantis* in promoting a balanced gut microbiome. Moreover, *B infantis* colonization is functionally relevant in terms of higher gene levels for HMO-metabolizing pathways and reduced abundance of pathobionts, which may translate into prevention of systemic inflammation.^4^ *Bifidobacteria*, in contrast to *Lactobacilli*, are highly adapted to the infant gut conditions, which may explain a reduced colonization rate of *L acidophilus* in verum-treated infants. The reduced prevalence of BB-12 as compared with *B infantis* highlights how species- and strain-level differences between bacteria are important to their clinical impact. *B infantis* principally internalizes substrates such as fucose and sialic acid without sharing^26^ cross-feeding effects by other substrate-sharing *Bifidobacteria* species that may be important for microbiome maturation. We did not observe a group difference in alpha diversity, which argues against the theoretical concern that by increasing diversity probiotics might also increase pathogen carriage.^27^

The PRIMAL trial unveiled a remarkable phenomenon of frequent environmental acquisition of probiotics within the hospital environment. It is well acknowledged that hospital environments differ in their microbial signature and that bacterial communities on patients and room surfaces may become increasingly similar over the course of a hospital stay.^28^ We elucidated potential causes for environmental uptake, which occurred even in hospitals that had not used probiotics before initiation of the study. A significant driver was cobedding with a verum-treated sibling and exposition units to probiotics, ie, having a room neighbor in the verum group or a nonstudy probiotic-treated infant (aged <28 weeks of gestation). These observations raise questions for future trials. How does the introduction of a probiotic influence microbial entropy on a NICU (seeding the NICU by treating the individual)? A cluster randomized clinical trial design might have the advantage to account for NICU inherent aspects of cross-contamination, eg, product handling and administration. Our data also reinforced the importance of hygiene measures, as cross-contamination may occur by hands. Finally, should siblings be allocated in the same treatment group in randomized clinical trials, as data from parental questionnaires suggest?^29^ This may enhance the consent rate but diminish the opportunity to study gene-environment interactions.

### Strengths and Limitations

The strengths of this trial are large sample size, representative study population with a high risk for dysbiosis, early start of intervention, and high-resolution microbiome analysis. We thoroughly investigated adverse events, which are not consistently reported in many trials on probiotics.^11,12^ Our trial raised no concerns on short-term safety, particularly no bacteremia with probiotics. The incidence of culture-confirmed late-onset sepsis was 3.1% and within the predicted range of 1.5% to 6%.^30^

This study also has some limitations. First, postponement of study sites’ responses to queries and further lockdown restrictions due to the COVID-19 pandemic led to a significant delay in the interim analysis. When the decision was made to stop further enrollment, 646 infants had already been recruited. Second, the primary end point—local MDRO+ screening results—was selected for pragmatic reasons, variability in microbiological testing was noted; this explains marked discrepancy to central standardized MDRO+ testing. We also acknowledge a comparably high rate of infants born by cesarean delivery in our study. Although the mode of delivery had no major impact on MDRO+ colonization in regression models, it may limit the external validity of our data. Fourth, we noted a lack of adherence to block randomization particularly due to shortage of boxes for intervention in specific strata at the end of the study affecting 110 infants (55 each group, 18%). After informed consent, the investigators on site had selected the remaining boxes from different gestational age or sex strata than the study protocol intended. Randomization nevertheless resulted in similar clinical characteristics between the groups.

## Conclusions

Results of the PRIMAL randomized clinical trial showed that multistrain probiotics did not reduce the incidence of MDRO+ colonization at day 30 of life in preterm infants but modulated their microbiome toward eubiosis. Understanding the complex interplay between microbiome-modulating agents, hospital environment, and clinical outcome is essential for refining interventions. This underscores the need for long-term follow-up as performed in the PRIMAL cohort^11^ and extended research in this area.

## Notes

Supplement 1.
Trial Protocol.

Supplement 2.
Statistical Analysis Plan.

Supplement 3. **eTable 1.** Probiotic Bacteria Reference Genomes **eTable 2.** MDRO+ Pathogens Reporting Criteria **eTable 3.** PERMANOVA 16S BC-Distance **eTable 4.** Variables Tested in MetadeconfoundR **eTable 5.** Software and Databases **eTable 6.** ProTection SNVs for *B. Infantis* Probiotic Strain **eTable 7.** Screened Pathogens by mOTU ID **eTable 8.** Data for Modeling **eTable 9.** Maternal Data **eTable 10.** Treatments and Continuous Outcomes of the Study Population **eTable 11.** Interim Analysis **eTable 12.** Reporting of Adverse Events **eTable 13.** Probiotic Strain (PS) Presence at Day 30 by Treatment Group **eTable 14.** Logistic Regression Model With Hospital as a Random Effect **eTable 15.** Secondary Analysis of Outcomes by Presence of Probiotic *B. Infantis* at Day 30 **eTable 16.** Number of Enrolled Infants per Site **eFigure 1.** Study Concept of the Multicentre Phase 3 PRIMAL Trial **eFigure 2.** Model of Eubiosis Based on Published Metagenomes **eFigure 3.** Enrollment, Randomization, and ITT Population of the Interim Analysis **eFigure 4.** Central MDRO+ Assessment of Day30 Samples According to Intervention **eFigure 5.** Environmental Uptake of Probiotic Strains **eFigure 6.** Microbiome Variability and Impact of Verum Treatment **eAppendix 1.** Study Efficacy Outcomes **eAppendix 2.** Statistical Analysis and Biostatistics for Microbiome Analysis **eAppendix 3.** Microbiome Composition Analysis **eAppendix 4.** Eubiosis Score Modeling **eAppendix 5.** EMMA Full-Term Cohort **eAppendix 6.** Environmental Uptake of Probiotic Strains **eAppendix 7.** Bifidobacterium Relative Abundance
eReferences

Supplement 4.
Data Sharing Statement.
