# Supplementary material for: Bifidobacterium and Lactobacillus Probiotics and Gut Dysbiosis in Preterm Infants: The PRIMAL Randomized Clinical Trial
Source: JAMA Pediatr. 2024 Aug 5;178(10):985–95. doi: 10.1001/jamapediatrics.2024.2626 (PMC12549143; doi:10.1001/jamapediatrics.2024.2626)
Supplement: Supplement 1. — Trial Protocol. [file jamapediatr-e242626-s001.pdf]

***Study protocol* for the clinical trial of the PRIMAL consortium**

Effect of *Bifidobacterium* and *Lactobacillus* probiotics on gut dysbiosis in preterm infants: The PRIMAL randomized controlled phase 3 trial

1.Background

It is a general assumption that the healthy ‘gold-standard’ microbiome (eubiosis) in newborns is derived from full term, vaginally delivered, antibiotic-„naive“, breastfed infants. In the context of preterm infants, gut dysbiosis is defined as a microbiome deviation from the healthy state including reduced microbial diversity, delayed colonization and decreased abundance of *Bifidobacteria* as well as a deficient capacity to control the colonisation with multidrug-resistant organisms (MDRO) and/or gram-negative bacteria with high epidemic potential (MDRO+) (2-7). The colonization with MDRO+ are emerging healthcare problems causing infection outbreaks, in particular in highly vulnerable infants. Therefore it will be used in this multicenter study as a surrogate for gut dysbiosis to be prevented by probiotics (primary endpoint).

The population of preterm infants born between 28 and 32 week exhibits a high risk for exposure to microbiome disturbing factors, i.e. delivery via caesarean section, formula feeding, primary colonisation with hospital rather than maternal flora and antibiotics (2-7). Previous studies suggested that the metabolic capacity of the microbiome is likely to be influenced by these exposures, as indicated by the reduced levels of bioactive short-chain fatty acids in the microbiota of preterm infants compared with term infants (7). There is an urgent need for a more in-depth analysis of gut dysbiosis in a clinical study context of highly vulnerable preterm infants as dybiosis patterns may precede the development of sepsis and necrotising enterocolitis (NEC) (8-10). It has been hypothesised that dysbiosis contributes to immunological dysregulation and sustained inflammation. Both are very probable causes of long-term health problems in preterm infants, including chronic lung disease, growth failure, metabolic disorders and an adverse neurodevelopmental outcome (11,12). Well defined ‘risk’ and ‘resilience’ microbiome patterns may eventually serve as biomarkers or targets for modifications. Several approaches to modify gut dysbiosis in adults, such as selective decontamination, use of prophylactic antibiotics and faecal transplantation (13-15) are not feasible for preterm infants, in particular for ethical and safety concerns. Instead, probiotics with bacteria that excel as gut colonisers of healthy breast milk-fed infants (16) are promising targets to foster the early microbiome establishment and to modulate the dysbiotic preterm microbiome towards the microbiome of the full term infant. For instance, a probiotic

supplementation has been suggested to restore the microbiome of antibiotic-treated or caesarean-born term infants (5). Numerous studies on the therapeutic effects of probiotics in preterm infants (PIPS, single strain *Bifidobacterium breve* probiotics) have been performed. However, the results remain inconclusive due to a high variability in study protocols, target populations, probiotic formulations (eg, strain composition and inclusion of single vs multiple strains) and endpoints. The majority of studies have focused on short-term endpoints, in particular NEC and sepsis (18-24). Meta-analyses have found a benefit for preterm infants regarding the risk of adverse short-term outcome (19, 25). In contrast, the largest randomised controlled trial (RCT) to date, the PIPS study, did not find any benefit for preterm infants (24). However, controversy remains regarding the analysis and interpretation of the findings (26). The scientific uncertainty in regard to the efficacy and safety of probiotics is reflected in their heterogeneous use in medical practice. Prophylaxis with *B. longum and infantis/L. acidophilus* has routinely been adopted by several European neonatal intensive care units (NICUs), for example, in Austria (22), the Netherlands (23) and Germany (20,27). In contrast, the Norwegian community of neonatologists has stopped this approach after occurrence of sporadic probiotic strain sepsis cases (28), and NICUs in the USA are still reluctant to implement probiotic prophylaxis strategies since the effect sepsis-predisposing (29). As of yet, mechanistic data on how probiotics exert a potentially their effect in preterm infants are lacking and no study has directly assessed the effects of probiotics on the microbiome at high resolution. Pre-clinical data suggest that Bifidobacteria probiotics inhibit the growth of resistant bacteria (30,31). One study in adults did not prove probiotics to be preventive against colonization with MDRO (32).

In the context of preterm birth, the efficacy of probiotics might depend on the gut microbiome composition when the probiotics are administered ('baseline'). Potentially influencing factors, such as maternal microbiome/metabolome (33, 34), the perinatal exposure to antibiotics and the nutritional context (eg, human milk oligosaccharides, 35) were hardly considered in previous studies. Hence, an adequately powered RCT using a well-defined probiotic strain mix and validated clinical and microbiological outcome measures in preterm infants at risk of microbiome-related sequels will improve clarity in this field.

The specific study questions of this large, multicenter double, placebo-controlled trial is:

In preterm infants 28-32 weeks of gestation at high risk for dysbiosis, does multistrain probiotics with *B. animalis* subsp. lactis and *infantis and L. acidophilus* as compared to placebo (a) prevent colonisation with MDRO+ and (b) accelerate microbiome establishment towards the eubiosis microbiome of healthy full-term infants?

We will investigate pre-defined clinical and safety endpoints such as sepsis, gastrointestinal complications, death and growth during primary stay in hospital. In addition, we hypothesise that the administration of probiotics will decrease the risk for infections, wheezing and growth deficits in the first year of life. This RCT is the core study of our PRIMAL consortium, which more broadly investigates the interaction between gut microbiome and immunity on a cellular level at the beginning of life.

2. Methods

2.1. Study design

The PRIMAL clinical study is an investigator-initiated, multicenter double-blinded, placebo-controlled randomized, group-sequential trial, conducted in 18 German Children's Hospitals with a tertiary NICU, including the University Children's Hospitals in Bochum, Bonn, Cologne,

Essen, Freiburg, Hannover, Heidelberg, Homburg, Jena, Lübeck and Tübingen, and the regional Children's Hospitals in Aschaffenburg-Alzenau, Bremen, Hamburg-Wilhelmstift, St. Vincenz Paderborn, Rostock-Klinikum Südstadt, Schwerin and Wiesbaden. The institutional review boards of all participating centers approved this study trial protocol. Between 1 April 2018 and 31 March 2020 preterm infants born between 28+0 and 32+6 weeks of gestation are assessed for eligibility, approached for informed consent and randomised in the first 48 hours to receive placebo or probiotics. Study drug or placebo will be administered once daily for 28 days after randomisation. Placebo is essential for study design in order to clearly address effcacy and safety issues. Physicians, nursing staff, parents and all study personnel are blind to the intervention.

The trial protocol complies with the Declaration of Helsinki, the current revision of International Conference on Harmonisation (ICH) Topic E6, and the Guidelines for Good Clinical Practice (GCP). Trial reporting was in alignment with current Consolidated Standards of Reporting Trials (CONSORT) and SPIRIT recommendations (36,37). The trial will be monitored by the Centre for Clinical Studies (CCS) at the University of Lübeck, Germany.

An independent data safety monitoring board (DSMB) will convene on a regular basis to review interim analyses for safety and efficacy and includes a parent representative (Barbara Mitschdörfer), a statistician (PD Dr. Markus Pfirrmann) and two experienced neonatologists (Prof. Peter Bartmann and Prof. Bernd Roth). The statistical analysis follows the statistical

analysis plan which is provided by the Institute of Medical Biometry and Statistics, University of Lübeck.

The method of analysis includes the denomination of primary endpoints (intention-to-treat analysis), identification of key clinical and safety outcomes (safety analysis), and explorative or ancillary measures (as-treated analysis).

2.2. Study participants

Infants will be considered for inclusion within the first 48 hours of life for enrollment if they are (1) born at a study center (2) within the gestational age range of 28 weeks 0 days and 32 weeks 6 days. Infants are ineligible if, in the judgment of the attending neonatologist, they showed malformations that are not compatible with survival beyond the first 48 hours of life, or conditions that severely affected the gastrointestinal tract (appropriate enteral feeding not possible, surgery in the neonatal period or NEC Bell's stage ≥1B). Reasons for non-participation are documented in the screening log. Eligible patients are identified by an approved study investigator (SI), who maintains a log of all screened preterm infants that is available for later monitoring. The SI discusses the background and objectives of the study with the caregivers of the patient and gives information on procedures and interventions. If written informed consent is given, the SI assigns a patient identification number, randomises the patient, collects baseline demographic variables and completes the according CRF.

2.3. Randomisation

Participants will be randomized to a multi-strain *B. animalis* subsp. lactis and *B. infantis and L. acidophilus* probiotic (verum) or placebo in a 1:1 ratio in the first 48 hours after birth. Randomisation will be organized centrally by the IMBS Lübeck using standard operating procedures (SOPs) to guarantee concealment of allocation, block randomisation with randomly varying block length and with stratification by (1) center, (2) sex and (3) gestational age, i.e. a) 28 weeks 0 days to 30 weeks 6 days and b) 31 weeks 0 days to 32 weeks 6 days. Multiple births will be randomized independently. The lists are sent to the pharmacy of the University Medical Centre of Lübeck which will prepare study treatment boxes for patients at all study sites according to the randomisation schedule. The boxes will be consecutively numbered according to the randomisation schedule and sent to the study centers according to requirements.

2.4. Intervention

Verum and placebo will be provided in daily dose capsules of identical appearance and administered over 28 days. Probiotics are taken from a single batch of the probiotic-mixture consisting of *Bifidobacterium* (*B.*) *longum* subsp. *infantis*, *B. animalis* subsp. *lactis* (BB-12) and *Lactobacillus acidophilus* (La-5), at a dose of 1.5×10^9 CFUs of each strain per capsule. Placebo will be cornstarch powder of similar color and odor as the verum. Capsules for a complete course for one infant will be packaged into one box and stored at 4°C until usage.

For quality assurance, probiotic bacteria in verum powder will be analyzed by whole genome DNA sequencing. DNA extraction was performed using the Qiagen® DNeasy PowerSoil Pro Kit, libraries will be prepared using the Nextera XT DNA Library Preparation Kit. Sequencing will be performed on an Illumina HiSeq 4000 platform (Illumina, San Diego, CA, USA) with 2x150 bp paired-end reads. Reads are processed to remove low quality data using ngless v1.1. Nucleotide calls with a Phred score of less than 25 are removed from the 3' end. Any reads that are less than 45 nucleotides long after removal of low-quality nucleotide calls will be discarded. Reads representing human DNA will be identified by comparing all reads' sequence similarity to the human reference genome. Reads with greater than 90% similarity to the human genome are removed from the analysis. Reads are assembled using Spades and compared to high quality, complete genomes to identify the closest match using the PATRIC platform and database.

Probiotic samples will also be tested every 6 months for microbiological quality (PRIMAL Faecal Core Centre in Mainz, FCCM), as dose decay has been an issue in the PIPS trial (24).

After inclusion of a patient, the boxes/blisters remain at the bed site at room temperature. The first dose should be administered as soon as possible after randomisation with the start of enteral feeding. According to an SOP provided by the clinical project management, the capsule content will be dissolved in milk/formula or glucose solution and administered orally or per nasogastric tube. The intervention will be considered successful if at least part of the capsule content was administered, if the intervention phase was not interrupted for more than 2 consecutive days and if it lasted for at least 14 days. The study sites are encouraged to preferably feed colostrum or breast milk. A record of doses omitted will be kept. Participants, families, healthcare providers, data collectors, outcome adjudicators and data analysts were blinded to the intervention. All aspects of clinical management, including discontinuation of the study medication due to medical reasons, are at the discretion of the attending neonatologists who should follow local, national and international guidelines.

2.5. Sample collection

After informed consent, the first two study investigations are performed by the attending physicians of the study site after randomisation (baseline meconium sample or first collectable stool, day 1–3 of life - ideally before start of intervention - time point 1) and 28 days after the start of intervention or discharge (day 28–30 of life; time point (2), while the infants are cared under controlled conditions in neonatal units. The third assessment is performed at 12 months (time point 3) corrected age at the study site by the PRIMAL study team from the lead site at the University of Lübeck or the local principal investigators. For microbiome analysis stool samples are collected. At the time points (1) and (2), three faecal aliquots are obtained from one sample. Timepoint of collection (all aliquots) and timing until freezing (aliquots 2, 3) will be documented for quality assurance. Aliquot 1 (room air) is sent to the local microbiological laboratory for microbiological culture and identification oft he primary endpoint of the study, colonisation with MDRO+ as per local assessment:

(a) Multidrug-resistant strains (multiresistant gram-negative bacteria (MRGN); 2 MRGN; 3 MRGN; 4 MRGN; methicillin-resistant *Staphylococcus aureus*.

(b) Pathogens without resistance characteristics but highly epidemic potential for outbreaks (*Serratia* spp; *Pseudomonas* spp; *Klebsiella* spp; *Enterobacter* spp).

This procedure is in line with the German screening recommendations according to the Kommission für Krankenhaushygiene und Infektionsprävention (KRINKO, 38). The results of the screening at time point 2 will be documented on the CRF-2 for the microbiological definition of 'gut dysbiosis'. Aliquot 2 and 3 are uniformly packed and frozen according to protocol and stored at −80°C, until the aliquots are sent to the PRIMAL FCCM (Dr. Claudius Meyer) for an independent microbiological analysis (central review) according to KRINKO from previously frozen material. For a nested-subgroup study of n=120 infants (n=60 verum, n=60 placebo) with complete faecal sample sets of time points 1–3, the FCCM receives a maternal stool sample for analysis taken at any time after birth during hospital stay. Preparation of stool samples, DNA extraction and microbiome sequencing is performed using two approaches:

(1) Amplicon-based sequencing of the V4 region of the bacterial 16S rRNA genes (Illumina MiSeq in Mainz, Dr. Claudius Meyer, see below);

(2) Whole genome shotgun metagenomic sequencing of a subset of the samples and their respective mother samples (Illumina HiSeq 2000/2500 at the European Molecular Biology Laboratory (EMBL) Genomics Core Facility in Heidelberg, Dr. Sofia Forslund, Prof. Peer Bork). Using the marker-based microbiome profiles and additional metagenomic information,

the genetic and phylogenetic composition of the gut flora is profiled and assessed as outlined below.

At the 12-month follow-up visit, a stool sample of the infant is collected according to a home stool collection protocol similar to collection an storage procedures for timepoints 1 and 2. The protocol consists of providing the family with a specimen collection kit and an insulated envelope. The samples should be as fresh as possible before follow-up visit, transported with cool packs and immediately frozen at −80°C on arrival at the study site. The aliquots are sent to FCCM and microbiome sequencing will be performed.

For immunophenotyping and experimental subprojects of the PRIMAL consortium, blood samples including plasma aliquots are collected at time points 1 to 3 at selected study sites (n=250, n=125 verum, n=125 placebo). For immuno-phenotyping, the peripheral blood mononuclear cells from whole blood are separated by ficoll density centrifugation, resuspended in wash buffer, and then pipetted into ZellSafe chips, using a standardised protocol. The chips are stored at 4°C until shipment to the PRIMAL Immunological Core Centre (ICC) in Hannover (Prof. Dr. Dorothee Viemann) for analysis of cellular markers of T-cells, B-cells, granulocytes, monocytes, NK-cells and stem cells. An analysis protocol including clinical metadata intergation will be developed in collaboration.

2.6. Data collection and sampling

Clinical metadata including a pre-defined set of antenatal, maternal and postnatal parameters of clinical assessment, treatment and outcomes will be documented in case report forms by attending physicians. Standardised CRFs are used to record information on the patient’s health at the time of birth (CRF-1; baseline), at day 28 after start of intervention (CRF-2; primary endpoint), at discharge (CRF-3), at the 6-month interview (CRF-4) and 12-month follow-up (CRF-5). Data collected at the follow-ups are collected to measure long-term outcomes. The forms are filled in manually by SIs in the study sites and are sent to the clinical project management (CPM) after the patients have been discharged. CRF-1 includes information on the birth, causes of preterm birth, age, weight, head circumference and length, bonding, antenatal treatments with antibiotics and on the mother (diet, habits, body weight). CRF-2 includes information on dysbiosis, growth parameters and detailed medical information on the treatment with antibiotics, invasive measures, feeding and on transfers to different hospital wards. Information on the use of mother’s own milk, donor breastmilk and formula in this population of preterm infants in the study NICUs will be collected. CRF-3 records growth parameters and details on feeding, pathogens in the event of sepsis and on

complications. CFR-4 data are collected in a telephone interview. Therefore, all caregivers will receive a diary at discharge to document infections, on medical consultations or hospital stays, antibiotic treatments, vaccinations and feeding. The interviews are coordinated by the CPM. CRF-5 data are obtained during the 1-year follow-up examination, which is coordinated by the CPM and performed at the study site. Next to details concerning age, weight and size, feeding, faecal sample collection will be performed to assess the sustainability of microbiome patterns throughout infancy.

2.7. Data handling and monitoring

The CRFs of the study sites are sent to the CPM. There, patient data are saved for further contacts of the families. All data will be sent completely pseudonymised to the ZKS/IMBS Lübeck, where incoming data are entered into the central research database. As personal data of the patients will be saved separately on a different server than the clinical database, data of individual participants cannot be 'reconstructed' by data mining or similar procedures. The procedures will closely follow the regulations as specified in the German data protection law. Data transfer between CCS Lübeck, the platform for microbiome analysis (FCCM/EMBL) and immune phenotyping (ICC Hannover/Homburg) is organised by a working group of platform members. The CPM will be responsible for maximising the output of PRIMAL clinical study. The steering group of the PRIMAL trial will also be contacted by external researchers who are interested in exploitation of the data. Data sharing will be made possible on publicly available data repostories.

2.8. Primary endpoint and secondary microbiome related outcomes

The primary efficacy endpoint is the rate of gut dysbiosis at day 30 of life. The surrogate definition of gut dysbiosis is based on the guideline definitions of the KRINKO (38,39): Colonisation with MDRO or bacteria with epidemic potential (*Enterobacter* spp, *Klebsiella* spp, *Serratia* spp, *Pseudomonas* spp) as detected by microbiological culture (primary endpoint). As secondary efficacy endpoints of gut dysbiosis we will perform central microbiological MDRO+ analysis and assess significant mirobiome deviations from the eubiosis pattern of healthy term infants (proportions of bacterial phyla, reduced diversity or specific features, such as increased virulence capacity and blooms of specific pathogens, functional changes such as reduced HMO genes; 40).

For central MDRO+ screening the stool samples will be thawd and cultured using BD Columbia CNA Agar with 5% sheep blood (Becton Dickinson, Heidelberg, Germany) for

detection of Gram-positive bacteria and chromID® CPS® Elite Agar plates (Biomérieux, Marcy l'Etoile, France) for Gram-negative bacteria. Classification of MDRO+ species will be performed with MALDI-TOF and antibiotic resistance (against penicillin, cephalosporin, carbapenem, fluoroquinolone and gentamicin) and screened on a VITEK machine (Biomérieux, Marcy l'Etoile, France.).

2.9. Microbiome composition analysis

Frozen stool samples will be used for further microbiome analysis if the following pre-analytical quality criteria are met, i.e. the sample has to remain frozen throughout the shipment process, and to be valid in terms of collection time point and start of intervention of the infant. The timepoint 2 sample was predefined for collection between Day 15 and Day 40 of life when the preceding treatment period lasted at least 14 days (before hospital discharge or transfer).

16S rRNA gene sequencing (16S data). For 16S rRNA gene sequencing, DNA extraction (Qiagen® DNeasy PowerSoil Pro Kit) and library preparation (Nextera XT DNA Library Preparation Kit) will be conducted. 16S rRNA gene sequencing (Illumina® MiSeq) will the V4 region (251 bp) using the primers 515f and 806r (forward: 5'-GTGCCAGCMGCCGCGGTAA-3', reverse: 5'-GACTACHVGGGTWTCTAATCC-3'). The obtained 16S rRNA gene reads are processed using DADA2. Before taxonomic annotation, quality control and removal of mitochondrial reads will be performed, amplicon sequence variants (ASVs) generated and the identified ASVs taxonomically annotated using the SILVA database.

Deep metagenomic sequencing (metaG data). For microbiome characterization at the species level, metagenomic sequencing (whole genome shotgun) will be conducted on a subset of timeoint 1 and 2 at the European Molecular Biology Laboratory (EMBL) Genomics Core Facility in Heidelberg, Germany. Libraries will be prepared using an automated liquid handling system (Beckman Coulter i7 Series) with the NEBNext Ultra II DNA Library Prep kit, targeting an insert size of 350-400 bp and incorporating Dual Index multiplex Oligos. The samples will be sequenced on the Illumina HiSeq 4000 platform (Illumina, San Diego, CA, USA) with 2x150 bp paired-end reads. Species profiles will be compiled using mOTUs. Strain-resolved detection results for the three probiotic treatment strains will be generated using genome-specific SNVs. Reference genomes of the strain of interest (SOI) will be identified by comparing metagenomic reads to public genomes using the PATRIC database. A mapping database will be created, comprising the closest reference genome and one other

species from the same genus, to prevent species-unspecific mapping of reads. Reads will be mapped to the reference genomes with a 99% identity threshold using ngless and bwa and only uniquely mapping reads will be kept. SNVs that differentiated the SOI from the closest reference are identified using metaSNV. Bioinformatic quality criteria will be defined for SNVs being higly distinctive of the probiotic strain. This will allow us to control for inherent differences among neonatal intensive care units, such as their microbial environment (cross-contamination among room neighbors), and for the maternal microbiome in a subset of infants. HMO genes will be detected by mapping the metagenomic reads against the GMGC database and annotating the resulting hits with functional groups using according software. Potential pathogens relevant to preterm infants to be detected by metagenomic species profiles will be compiled from guidelines recommendations for MDRO+ screening and from the literature (38, 39) and tested for their relative abundance according to verum or placebo. Bacterial-gene mediated antimicrobial drug resistances and virulence factors will be detected using most current databases.

Eubiosis score modeling. To address the concept of eubiosis measure post-intervention eubiosis rates at Day 30, we will establish a model of normative eubiosis using previously available data of previously published infant fecal metagenomes. Ideally, the model will differentiate the fecal microbiome of “probioic-naïve” preterm infants with dysbiosis from the eubiosis pattern typical for full term, vaginally delivered, healthy infants (“resurrection of the microbiome, 41). The model will use species and genus profiles to generate a score, from 0 to 1, that reflects the probability that a microbiome sample belongs to the “preterm” or the “full term” group (“eubiosis score”, higher values mean less likely to be dysbiotic). Two versions of the model will be made: one with genus profiles (based on 16 S data) and one with species profiles (based on metaG data). In parallel, we plan to enroll an independent full-term infant cohort as alternative approach to address eubiosis and compare to preterm infant datasets.

2.10. Pre-defined clincal and Z score outcomes

Key clinical safety outcomes were pre-specified based on their importance in relation to early microbiome establishment, gastrointestinal function and potential effect on long-term outcome. Weight gain will be estimated at Day 30 of life and at discharge. Neonatal sepsis was defined according to the criteria of the national infection surveillance system “NEO-KISS” (42,43). Clinical sepsis was defined as sepsis with at least two clinical signs (temperature > 38 °C or < 36.5 °C, tachycardia > 200/min, new onset or increased frequency of bradycardias or apneas, hyperglycemia > 140 mg/dl, base excess 1 mg/dl, immature

neutrophil/ total neutrophil ratio > 0.2, white blood cell count < 5/nl, platelet count < 100/nl) and antibiotic treatment for ≥ 5 days, but no proof of causative agent in the blood culture. Blood-culture proven sepsis is defined as clinical sepsis with proof of causative agent in the blood culture. We are particularly aware of the potential risk for sepsis with probiotic bacteria (28). If coagulase negative staphylococci (CoNS) were detected as a single pathogen in the blood culture, a CrP value of > 1 mg/dl will be mandatory to fulfill the definition of CoNS sepsis. Early onset sepsis, clinical or culture-proven, is defined as sepsis occurring within the first 72 h after birth, late onset sepsis was defined as sepsis occurring after 72 h (40,41). Based on our experience in more than 10 000 treated infants born in German Neonatal Network centres, where probiotics similar to the study medication are frequently used and infections with probiotic strains have not been observed, the safety of the intervention can be expected to be very high. We expect a NEC rate of 0.8%, clinical sepsis rate of 17.8% and culture-proven sepsis rate of 7.1% in preterm infants 28-32 weeks of gestation. All gastrointestinal complications such as NEC, FIP and abdominal surgery for meconium obstruction, volvulus or gastrointestinal hemorrhage will be independently reviewed by 3 experienced neonatologists to confirm the diagnoses and the potential relationship to the intervention made by the attending physician. Adverse and serious adverse events will be defined according to CTCAE criteria and ascertained for potential relatedness to the intervention. Any queries will be clarified with the principal investigator at the participating hospital.

Key endpoint(s) after discharge are clinical or laboratory signs related to infection, immunity and metabolism. In particular, these are infectious episodes in the first year of life, for example, otitis media, upper respiratory tract infections, bronchitis, gastrointestinal infections; number of antibiotic courses, as assessed by patient’s diary, telephone interviews and follow-up at 12months of age; wheezing episodes and atopic dermatitis as assessed by patient’s diary, telephone interviews and follow-up at 12 months of age; weight gain, growth and blood pressure.

2.11. Quality assurance and monitoring

During the clinical trial, quality control and assurance is ensured through on-site monitoring (CKS Lübeck), auditing and, if applicable, through supervision by the authorities. All investigators agree that the monitor visits before (pretrial visit) during and after completion of the study in order to ensure that the study is conducted, recorded and reported according to the

protocol, the SOPs, the GCP and the applicable regulatory requirements. The monitor provides each site with a written report, and sites have been required to respond to queries and resolve problems. In addition to these routine monitoring procedures, audits—by the sponsor or by authorities—will be conducted in the framework of the auditing system in accordance with ICH-GCP guidelines. In the context of an audit, the planning, conduction and analysis of a clinical trial will be analysed for compliance with the ICH guidelines. This will address, wh ether data handling, the organisational structure of the study centre, and the original documents are in accordance with the agreement between the sponsor and the study board. It will be the primary goal of the auditing to ensure that all of the data required for the interim and final analysis can be properly extracted from the files. SOPs on the administration of probiotics/placebo are shared among all study centres including at site training before start of enrolment. Central reporting of SAEs is provided. It is the obligation of the DSMB to monitor the course of the study, and if necessary, to give recommendations to the study administration for discontinuation or modification of the study. The primary principles are the ethical and safety aspects concerning the patients. The DSMB examines, whether the continuation of the study can be ethically justified, whether the safety of the patients is ensured, and whether the progress of the study is acceptable. For this, the DSMB has to be informed by the principal investigator about adherence to protocol, patient recruitment and any observed AEs. The DSMB receives the corresponding reports and in due time before the planned interim analysis. The composition and responsibilities of the DSMB, the structure and procedures of its meetings, and its relationship to other key study team members are laid down in a separate DSMB file.

2.12. Stopping rules

Any patient may be withdrawn from the study at any time, at the request of parents, for any reason, specified or unspecified, and without penalty or loss of benefits to which the patient is otherwise entitled. Patients, who are withdrawn from the study, will not be allowed to re-enter later. Infants may have to be excluded from the study during the course of the study in case of any of the following events: an interruption of enteral or oral feeding for more than 72 hours caused by severe gastrointestinal disorders, major gastrointestinal surgery or multiorgan dysfunction. The responsible investigator has the right to discontinue the study in infants that experience one or several of the following incidents (1) AEs which do not allow any further treatment with the study medication, (2) unacceptable study conduction when balancing risk and benefit, (3) technical-logistic problems. The study sites are instructed to prematurely

discontinue the study only, if substantial problems occur. All patients should be followed up and documented after discontinuation of the treatment, in order to record the data that is required in accordance with the intention-to- treat (ITT) principle and tob e able to provide comprehensive safety reports. The coordinating investigator will be informed immediately, should ethical or safety concerns occur at any study site. The trial will be stopped at any time, if this is recommended by the DSMB, based on severe safety concerns. Furthermore, the DSMB will rule on the completion or discontinuation of the study in the interim analysis. The coordinating principal investigator is authorised to exclude single centres in case of (1) inadequate recruitment, (2) insufficient quality of data or (3) other problems making the continuation of the study at that centre impossible.

2.13. Sample size calculation

The sample size calculation is based on the primary endpoint of local assessment of MDRO+ positivity in fecal samples of Day 28-30. In a preliminary evaluation of microbiological screening data, we found a risk of gut dysbiosis of 7.5% on day 28 of life in infants, who received probiotics, as compared with 15% in infants without probiotic prophylaxis. A group sequential plan will be used with interim analysis at 50% information time, a one-sided α=0.01 at the interim, and a futility stop of $\alpha0$=0.7. This corresponds to an acceptance bound of −0.524 and a rejection bound of 2.326 at the interim analysis. The rejection bound at the final analysis has been set at 2.075 (one-sided α=0.019). An adaptation of the number of interim analyses or the total sample size will be investigated using the conditional power approach. Stopping or adaptation will require recommendations by both the DSMB and the steering committee of the trial. In order to achieve a conservative sample size estimate, the additional power obtained from multiple offspring in a pregnancy has been neglected. The interim analysis will be performed with 161 infants per group, that is, 322 infants for the interim analysis, in total. For the final analysis, 327 infants need to be analysed per group to achieve 80% power using the one-sided 0.019 test level (continuity-corrected X2). Accordingly, the final analysis will be performed after randomisation of at least 654 infants, that is, if 327 infants in each group have been recruited. With the effect size used for sample size calculations, the power for stopping the trial for efficacy reasons at the interim analysis is 38%.

2.14. Statistical and bioinformatic analysis

The statistical analysis will be fixed in the statistical analysis plan. In brief, the primary endpoint of MDRO+ gut dysbiosis will be analyzed using the intention to treat (ITT) set as randomized with a generalized linear mixed effects model (GLM) with sex as a fixed factor, and study site and gestational age as random effects in two categories. To test the treatment effect, a Wald test for the log odds ratio (OR) will be used, and corresponding (1-α) confidence intervals will be presented with α chosen corresponding to the interim or final analysis. The total error level α is set to 0.025, and a one-sided test for superiority will be chosen. An interim analysis will be performed for efficacy and futility, as described above. Sensitivity analyses for the primary outcome will be conducted for the as-treated (AT) and per protocol (PP) sets. For the PP set, participants will be excluded from the ITT population if there were protocol violations for inclusion criteria, informed consent, duration/interruption of intervention or data documentation. Safety endpoints are analyzed in the safety analysis (SA) set which exclude participants from the ITT set without any drug administration, and patients will be analyzed as treated. Descriptive statistics will be provided stratified by treatment groups. Secondary outcomes, such as gut dysbiosis based on microbiome composition, will be analyzed in the AT analysis sets. The treatment effect will be modeled by a GLM approach adjusted for sex, study site and gestational age strata and by using a Wald test. As no adjustments for multiple comparisons will be made for secondary outcomes, the findings will be exploratory.

For ancillary studies to further evaluate the potential environmental uptake of verum probiotic *B. infantis* in the placebo group high resolution (SNV-level) detection of probiotic strains will be performed as described in the "metagenomic sequencing" section above. The abundance of verum *B. infantis* will be measured as the mean depth of coverage of the reference genome, normalised by the sequencing depth per sample, which was calculated as the Z score of the number of reads per sample, and compared between verum and placebo using Wilcoxon rank sum test. Probiotic presence will be adjusted for infant parameters (day of life at fecal sample collection, human milk feeding, days of antibiotic treatment, birth mode, sex, gestational age, maternal antibiotic treatment).

Biostatistics for microbiome analysis. The biostatistical analysis of microbiome composition will be conducted on the 16S dataset using the R environment. We will use Non-Metric Dimensional Scaling (NMDS) for multivariate analysis with Bray–Curtis dissimilarities between samples. To assess the impact of various factors on microbiome variability, permutational analysis of variance (PERMANOVA) on distance matrices (Bray-Curtis dissimilarities) will performed in the vegan package utilizing the adonis2 function. Bray-

Curtis dissimilarities are calculated based on relative abundances using the weighted distance function within the phyloseq. Furthermore, alpha-diversity measures (Shannon index, observed richness, Simpson index) based on untransformed counts will be calculated using the phyloseq package. Differences between groups will be compared using linear mixed-effect models. To address potential confounding effects of other microbiome shaping factors from infant clinical metadata on *B. infantis* abundance, a univariate correlation analysis will be conducted using MetadeconfoundR. Specifically, we will examine correlations between each microbial feature and each covariate, utilizing Spearman correlation and Mann-Whitney U test for continuous and binary covariates, respectively, including a post-hoc test. For each pair of correlated features, a mixed-effects model will be fitted, where the rank-transformed variable is used as the response, and the rank of the other feature served as the explanatory variable. Standardized nonparametric effect sizes, represented by Spearman's rho and Cliff's delta metrics, will be reported as the result of this analysis if the false discovery rate is <0.1. The same statistical approach will be used to assess co-correlations of the probiotic-assigned ASVs with other genera. To determine the ASVs that correspond to our probiotic strains, we will extract the 16S rRNA gene regions from the genomic data produced by sequencing the probiotic material and performing multiple sequencing alignments for each probiotic species using according software. Subsequently, the consensus of these alignments will be blasted against the extracted probiotic 16S rRNA gene sequences to pinpoint the precise amplicon region of the isolates. These sequences, once concatenated, will undergo another alignment phase, followed by the construction of a phylogenetic tree. This process will identify the ASVs to which the probiotic strains belong.

3. Parent involvement

The development of the research question and outcome measures and the design of this study were discussed with patient organisations (Bundesverband „Das frühgeborene Kind e.V.“) beforehand. The data safety monitoring involves a parents’ representative. The results will be disseminated in a deidentified fashion to study participants via different media as stated above. A potential burden of intervention will not be measures by patients or parents but by thorough safety measures of the study team as stated below. Families will be followed up in a regular interval and provided with understandable information on the key findings of the study. Children will receive birthday cards and invitations for follow-up for the PRIMAL trial. It is a clear request of parents‘ representatives to perform longitudinal assessments in the

PRIMAL cohort to address long-term outcome which will be subject for future ethical applications and grant proposals.

4. Ethics and dissemination

The dissemination of progress in PRIMAL rests on four columns: (1) The PRIMAL website (www. primal- studie.de), which depicts researchers, projects, publications, webcasts, allows for direct interaction with interested patients, professionals and the media, and is linked to social media activities of patient organisations; (2) a newsletter, which will be sent quarterly to all centres in order to motivate participating physicians and to address topics of discussion for the regular study meetings; (3) presentation at meetings, which will allow for discussions with

the scientific community; (4) publications, that is, reports on methodology and on various outcome variables, i.e. expected rates of safety outcomes as comapred tot he international perspective will be published in peer-reviewed journals. The patient organisations European Foundation for the Care of Newborn Infants and the Bundesverband 'Das frühgeborene Kind' e.V. have also expressed their interest in contributing to dissemination of results (eg, through electronic newsletter and social media channels).

All investigators have agreed on sharing of data and biomaterials. Authorship of resulting manuscripts will be based on guidelines of the International Committee of Medical Journal Editors. All the important modifications and amendments will be communicated to the involved parties. All SIs will have access to the final data set of the trial. The results of our trial will be published in peer-review journals, presented at scientific meetings and disseminated through the website of our PRIMAL consortium (www. primal- study. de) and via social media of parent organisations.

References

1 Pammi M, Cope J, Tarr PI, *et al*. Intestinal dysbiosis in preterm infants preceding necrotizing enterocolitis: a systematic review and meta-analysis. *Microbiome* 2017;5.

2 Salminen S*et al*. Influence of mode of delivery on gut microbiota composition in seven year old children. *Gut* 2004;53:1388–9.

3 Jakobsson HE, Abrahamsson TR, Jenmalm MC, *et al*. Decreased gut microbiota diversity, delayed Bacteroidetes colonisation and reduced Th1 responses in infants delivered by caesarean section. *Gut* 2014;63:559–66.

4 Härtel C, Hartz A, Pagel J, *et al*. NOD2 loss-of-function mutations and risks of necrotizing enterocolitis or focal intestinal perforation in very low-birth-weight infants. *Inflamm Bowel Dis* 2016;22:249–56.

5 Korpela K, Salonen A, Vepsäläinen O, *et al*. Probiotic supplementation restores normal microbiota composition and function in antibiotic-treated and in caesarean-born infants. *Microbiome* 2018;6.

6 Fouhy F, Watkins C, Hill CJ, *et al*. Perinatal factors affect the gut microbiota up to four years after birth. *Nat Commun* 2019;10:1517.

7 Arboleya S, Binetti A, Salazar N, *et al*. Establishment and development of intestinal microbiota in preterm neonates. *FEMS Microbiol Ecol* 2012;79:763–72.

8 Heida FH, van Zoonen A, Hulscher JBF, *et al*. A necrotizing enterocolitis-associated gut microbiota is present in the meconium: results of a prospective study. *Clin Infect Dis* 2016;62:863–70.

9 Mai V, Torrazza RM, Ukhanova M, *et al*. Distortions in development of intestinal microbiota associated with late onset sepsis in preterm infants. *PLoS One* 2013;8:e52876.

10 Warner BB, Deych E, Zhou Y, *et al*. Gut bacteria dysbiosis and necrotising enterocolitis in very low birthweight infants: a prospective case-control study. *The Lancet* 2016;387:1928–36.

11 Douglas-Escobar M, Elliott E, Neu J. Effect of intestinal microbial ecology on the developing brain. *JAMA Pediatr* 2013;167:374–9.

12 Groer MW, Gregory KE, Louis-Jacques A, *et al*. The very low birth weight infant microbiome and childhood health. *Birth Defect Res C* 2015;105:252–64.

13 Strenger V, Gschliesser T, Grisold A, *et al*. Orally administered colistin leads to colistin-resistant intestinal flora and fails to prevent faecal colonisation with extended-spectrum β-lactamase-producing enterobacteria in hospitalised newborns. *Int J Antimicrob Agents* 2011;37:67–9.

14 Narayan NR, Méndez-Lagares G, Ardeshir A, *et al*. Persistent effects of early infant diet and associated microbiota on the juvenile immune system. *Gut Microbes* 2015;6:284–9.

15 Zivkovic AM, German JB, Lebrilla CB, *et al*. Human milk glycobiome and its impact on the infant gastrointestinal microbiota. *Proc Natl Acad Sci U S A* 2011;108:4653–8.

16 Underwood MA, German JB, Lebrilla CB, *et al*. Bifidobacterium longum subspecies infantis: champion colonizer of the infant gut. *Pediatr Res* 2015;77:229–35.

17 Tarr PI, Warner BB. Gut bacteria and late-onset neonatal bloodstream infections in preterm infants. *Semin Fetal Neonatal Med* 2016;21:388–93.

18 AlFaleh K, Anabrees J. Probiotics for prevention of necrotizing enterocolitis in preterm infants. *Cochrane Database Syst Rev* 2014;4.

19 Olsen R, Greisen G, Schrøder M, *et al*. Prophylactic probiotics for preterm infants: a systematic review and meta-analysis of observational studies. *Neonatology* 2016;109:105–12.

20 Härtel C, Pagel J, Rupp J, *et al*. Prophylactic use of Lactobacillus acidophilus/Bifidobacterium infantis probiotics and outcome in very low birth weight infants. *J Pediatr* 2014;165:285–9.

21 Denkel LA, Schwab F, Garten L, *et al*. Protective effect of Dual-Strain probiotics in preterm infants: a multi-center time series analysis. *PLoS One* 2016;11:e0158136.

22 Repa A, Thanhaeuser M, Endress D, *et al*. Probiotics (Lactobacillus acidophilus and Bifidobacterium infantis) prevent NEC in VLBW infants fed breast milk but not formula. *Pediatr Res* 2015;77:381–8.

23 Samuels N, van de Graaf R, Been JV, *et al*. Necrotising enterocolitis and mortality in preterm infants after introduction of probiotics: a quasi-experimental study. *Sci Rep* 2016;6:31643.

24 Costeloe K, Hardy P, Juszczak E, *et al*. Bifidobacterium breve BBG- 001 in very preterm infants: a randomised controlled phase 3 trial. *The Lancet* 2016;387:649–60.

25 Dermyshi E, Wang Y, Yan C, *et al*. The "Golden Age" of Probiotics: A Systematic Review and Meta-Analysis of Randomized and Observational Studies in Preterm Infants. *Neonatology* 2017;112:9–23.

26 Deshpande G, Rao S, Athalye-Jape G, *et al*. Probiotics in very preterm infants: the PiPS trial. *The Lancet* 2016;388.

27 Härtel C, Pagel J, Spiegler J, *et al*. Lactobacillus acidophilus/Bifidobacterium infantis probiotics are associated with increased growth of VLBWI among those exposed to antibiotics. *Sci Rep* 2017;7:5633.

28 Esaiassen E, Cavanagh P, Hjerde E, *et al*. *Bifidobacterium longum* Subspecies *infantis* Bacteremia in 3 Extremely Preterm Infants Receiving Probiotics. *Emerg Infect Dis* 2016;22:1664–6.

29 Gibson MK, Pesesky MW, Dantas G. The Yin and Yang of Bacterial Resilience in the Human Gut Microbiota. *Journal of Molecular Biology* 2014; 426 (23): 3866–76. https://doi.org/10.1016/j.jmb.2014.05.029.

30 Moubareck C, Lecso M, Pinloche E, et al. Inhibitory Impact of Bifidobacteria on the Transfer of Beta-Lactam Resistance among Enterobacteriaceae in the Gnotobiotic Mouse Digestive Tract. *Applied and Environmental Microbiology* 2007; 73 (3): 855–60. https://doi.org/10.1128/AEM.02001-06.

31 Gāliņa D, Valdovska A. *Effect of Probiotics and Herbals on Health and Shedding of Resistant Escherichia Coli in Piglets.* Latvia University of Agriculture 2017 https://doi.org/10.22616/rrd.23.2017.037.

32 Bar-Yoseph H, Hussein K, Braun E, Paul M. Natural History and Decolonization Strategies for ESBL/carbapenem-Resistant Enterobacteriaceae Carriage: Systematic Review and Meta-Analysis. *The Journal of Antimicrobial Chemotherapy* 2016; 71 (10): 2729–39.

33 Baumann-Dudenhoeffer AM, D'Souza AW, Tarr PI, *et al*. Infant diet and maternal gestational weight gain predict early metabolic maturation of gut microbiomes. *Nat Med* 2018;24:1822–9.

34 Elovitz MA, Gajer P, Riis V, *et al*. Cervicovaginal microbiota and local immune response modulate the risk of spontaneous preterm delivery. *Nat Commun* 2019;10:1305.

35 Autran CA, Kellman BP, Kim JH, *et al*. Human milk oligosaccharide composition predicts risk of necrotising enterocolitis in preterm infants. *Gut* 2018;67:1064–70.

36 Moher D, Hopewell S, Schulz KF, *et al*. CONSORT 2010 explanation and elaboration: updated guidelines for reporting parallel group randomised trials. *BMJ* 2010;340:c869.

37 Schulz KF, Altman DG, Moher D, *et al*. CONSORT 2010 statement: updated guidelines for reporting parallel group randomised trials. *BMJ* 2010;340.

38 Christoph J, Dame C, Eckmanns T, *et al*. Practical implementation and hygienic and infection control consequences of the colonization screening of preterm infants and newborns in intensive care: Additional recommendation of the KRINKO at Robert Koch institute, Berlin, for the implementation of recommendations for the prevention of healthcare-

associated infections in neonatal intensive care patients with a birth weight < 1500g from 2007 and 2012 (The article was published in German: Praktische Umsetzung sowie krankenhaushygienische
und infektionspräventive Konsequenzen des mikrobiellen Kolonisationsscreenings bei intensivmedizinisch behandelten Früh- und Neugeborenen: Ergänzende Empfehlung der KRINKO beim Robert Koch-Institut, Berlin, zur Implementierung der Empfehlungen zur Prävention nosokomialer Infektionen bei neonatologischen Intensivpflegepatienten mit einem Geburtsgewicht unter 1.500 g aus dem Jahr 2007 1 und 2012 2). *Epidemiologisches Bulletin* 2013;42:421–33.

39 Seidel J, Haller S, Eckmanns T, Harder T Routine Screening for Colonization by Gram-Negative Bacteria in Neonates at Intensive Care Units for the Prediction of Sepsis: Systematic Review and Meta-Analysis. *The Journal of Hospital Infection* 2018; 99 (4): 367–80. https://doi.org/10.1016/j.jhin.2018.03.017.

40 Vangay P, Ward T, Gerber JS, *et al*. Antibiotics, pediatric dysbiosis, and disease. *Cell Host Microbe* 2015;17:553–64.

41 Pamer EG. Resurrecting the Intestinal Microbiota to Combat Antibiotic-Resistant Pathogens. *Science* 2016; 352 (6285): 535–38. https://doi.org/10.1126/science.aad9382.

42 Geffers C, Baerwolff S, Schwab F, Gastmeier P. Incidence of Healthcare-Associated Infections in High-Risk Neonates: Results from the German Surveillance System for Very-Low-Birthweight Infants. *The Journal of Hospital Infection* 2008; 68 (3): 214–21. https://doi.org/10.1016/j.jhin.2008.01.016.

43 Härtel C, Hartz A, Bahr L, et al. Media Stories on NICU Outbreaks Lead to an Increased Prescription Rate of Third-Line Antibiotics in the Community of Neonatal Care. *Infection Control and Hospital Epidemiology: The Official Journal of the Society of Hospital Epidemiologists of America* 2016; 37 (8): 924–30. https://doi.org/10.1017/ice.2016.95.
