# Supplementary material for: Bifidobacterium and Lactobacillus Probiotics and Gut Dysbiosis in Preterm Infants: The PRIMAL Randomized Clinical Trial
Source: JAMA Pediatr. 2024 Aug 5;178(10):985–95. doi: 10.1001/jamapediatrics.2024.2626 (PMC12549143; doi:10.1001/jamapediatrics.2024.2626)
Supplement: Supplement 2. — Statistical Analysis Plan. [file jamapediatr-e242626-s002.pdf]

Hinweis: *Die kursiv geschriebenen Hinweise dienen als Hilfestellung bei der Erstellung und sind bei der Umsetzung des statistischen Analyseplans zu löschen. Grau unterlegte Passagen sind Textvorschläge, die so übernommen oder ergänzt werden können. Platzhalter sind ebenfalls grau hinterlegt und zusätzlich in eckige Klammern gesetzt. Weder kursiv noch grau hinterlegter Text ist zu übernehmen.*

Statistical Analysis Plan (SAP)

Efficacy of probiotics to prevent gut-dysbiosis in preterm infants of 28+0 to 32+6 weeks of gestation: a randomized, placebo-controlled double-blind trial

Prägung der Immunität am Lebensbeginn

PRIMAL CLINICAL STUDY

Version V01

Gültig ab 20.04.2020

SAP Changes

Hinweis: *Wenn Änderungen am SAP vorgenommen werden müssen, sind diese entsprechend zu dokumentieren. Ebenfalls muss eindeutig angegeben werden, auf welche Version des Prüfplans der vorliegende SAP beruht.*

Nr	Description	Date	Version	Version Protocol
01	*Erstfassung*	*20.04.2020*	*01*	*5*

Unterschriften

Die unterschreibenden Personen haben den vorliegenden SAP gelesen und überarbeitet und erklären sich mit dessen Inhalt einverstanden.

Lübeck, ______________________________

Dr. Maren Vens, Studienstatistikerin

Lübeck, ______________________________

Prof. Dr. Inke R. König, Unabhängige Statistikerin

________, ______________________________

Prof. Dr. med. Christoph Härtel, für den Sponsor und als koordinierender Hauptprüfer

Abbreviations

Abbreviation	Description
AE	Adverse Event
AT	As Treated
AZ	Aktenzeichen (reference number)
BfArM	Bundesinstitut für Arzneimittel und Medizinprodukte (Federal Institute for Drugs and Medical Devices)
CDM	Central Data Management
CI	Confindence Interval
CONSORT	Consolidated Standards of Reporting Trials
cm	Centimeter
CPC	Cerebral Performance Catergory
CRF	Case Report Form
CTCAE	Common Terminology Criteria for Adverse Events
DSGVO	Datenschutzgrundverordnung (general data protection regulation)
DSMB	Data Safety Monitoring Board
DVP	Data Validation Plan
EMBL	European Molecular European Laboratory
FA	Full Analysis
FU	Follow-Up
g	Gramm
GW	Gestational Week
h	hour
H_0	Null hypothesis
H_1	Alternative hypothesis
HLGT	High Level Group Term
HLT	High Level Term
ICH	International Council for Harmonisation of Technical Requirements for Pharmaceuticals for Human Use
ID	Identification
ITT	Intention-To-Treat
kg	Kilogramm
KRINKO	Kommission für Krankenhaushygiene und Infektionsprävention
LLT	Low Level Term
Log	Natural Logarithm
MedDRA	Medical Dictionary for Regulatory Activities
MDRO	Multi-Drug Resistant Organism
NEC	Necrotizing enterocolitis
Neo-KISS	Krankenhaus-Infektions-Surveillance-System für neonatale Intensivstationen
OR	Odds Ratio
PID	Personal Identification
PP	Per Protocol

Abbreviation	Description
PRIMAL	Prägung der Immunität Am Lebensbeginn
PT	Preferred Term
R	R Software
SA	Safety Analysis
SAE	Serious Adverse Event
SAP	Statistical Analysis Plan
SAS	Statistical Analysis System, Business Analytics und Business Intelligence Software
SDM	Study Data Management
SND	Severe neurological deficit
SOC	System Organ Class
SOP	Standard Operating Procedure
spp.	Species
°C	Degrees Celsius
α	Type I error
α_a	Type I error adjusted for analysis a, where a is either interim or main analysis
-imp	Imputed data set

Table of contents

1. Aim 10
2. Responsibilities 10
3. Study Design und Amendments 11
   3.1 Study 11
   3.2 Study Amendments 12
      3.2.1 Ethics committee 12
      3.2.2 BfArM 12
4. Background 12
   4.1 Trial Objective 12
   4.2 Definitions 12
   4.3 Outcome Measures 12
      4.3.1 Primary Outcome Measure 12
      4.3.2 Secondary Outcome Measures 12
         4.3.2.1 Efficacy 12
            4.3.2.1.1 Gut dysbiosis 12
            4.3.2.1.2 Clinical or laboratory signs related to infection and secondary outcomes related to immunity 13
               Clinical sepsis during primary hospital stay 13
               Blood-culture proven sepsis during primary hospital stay 13
               Postnatal exposure to antibiotics during primary hospital stay 13
               Infectious episodes in the first year of life 13
               Wheezing episodes 13
               Atopic dermatitis 13
            4.3.2.1.3 Clinical or laboratory signs related to metabolism 13
               Growth parameters 13
               Nutritional aspects 14
               Metabolism/Vital signs 14
         4.3.2.2 Safety/Tolerability 14
            4.3.2.2.1 Extent of exposure 14
            4.3.2.2.2 Serious and non-serious events 14
            4.3.2.2.3 Laboratory tests 14
            4.3.2.2.4 Vital signs 14
         4.3.2.3 Quality of Life 14
         4.3.2.4 Health economics 14
5. Database 14
   5.1 Incomplete Treatments, Drop-outs and Incomplete Observation 14
   5.2 Protocol Deviations 15
6. Analysis Timing 16
7. Sets of Patients 17

7.1 Safety Analysis (SA) Set 17
7.2 Intention-To-Treat (ITT) Set 17
7.3 Full Analysis (FA) Set 17
7.4 Per Protocol (PP) Set 17
7.5 As Treated (AT) Set 18
7.6 Application of Analysis Sets 18
8. Handling of Missing Values, Missing Data and Outliers 18
8.1 Missing values, Missing Data 18
8.2 Outliers 19
9. Statistical analyses/methods 19
9.1 Subject disposition 19
9.2 Analysis of Intervention Group Comparability 19
9.2.1 Demographics and Baseline Characteristics 20
9.2.2 Prior or concomitant diseases and risk factors 20
9.2.3 Prior or concomitant medication 21
9.3 Exposition to treatment/Compliance 21
9.4 Primary Analyses 21
9.4.1 Analysis assumptions and alternative analyses 22
9.4.2 Imputation 22
9.4.3 Sensitivity analysis 22
9.5 Secondary analyses 22
9.5.1 Efficacy 22
9.5.1.1 Gut Dysbiosis 22
9.5.1.1.1 Analysis assumptions and alternative analyses 23
9.5.1.1.2 Sensitivity analysis 23
9.5.1.2 Clinical or laboratory signs related to infection and secondary endpoints related to immunity 23
9.5.1.2.1 Clinical sepsis during primary hospital stay 23
Analysis assumptions and alternative analyses 23
Sensitivity analysis 23
9.5.1.2.2 Blood-culture proven sepsis during primary hospital stay 23
Analysis assumptions and alternative analyses 24
Sensitivity analysis 24
9.5.1.2.3 Postnatal exposure to antibiotics during primary hospital stay 24
Daily dose per kilogram of body weight and treatment with antibiotics per 1000 patient days 24
Number of antibiotic cycles 24
Sensitivity analysis 25
9.5.1.2.4 Infectious episodes in the first year of life 25
Analysis assumptions and alternative analyses 26

Sensitivity analysis 26
9.5.1.2.5 Wheezing episodes during the first year of life 26
Analysis assumptions and alternative analyses 26
Sensitivity analysis 26
9.5.1.2.6 Atopic dermatitis during the first year of life 26
Analysis assumptions and alternative analyses 27
Sensitivity analysis 27
9.5.1.3 Clinical or laboratory signs related to metabolism 27
9.5.1.3.1 Growth parameters 27
Analyses assumptions and alternative analyses 28
Sensitivity analysis 28
9.5.1.3.2 Nutritional aspects 28
Analysis assumptions and alternative analyses 28
Sensitivity analysis 29
9.5.1.3.3 Metabolism/Vital signs 29
Analyses assumptions and alternative analyses 29
Sensitivity analysis 29
9.5.2 Safety/Tolerability 29
9.5.2.1 Extent of exposure 29
9.5.2.2 Serious and non-serious adverse events 29
9.5.2.3 Laboratory tests 31
9.5.2.4 Vital signs 31
9.5.3 Quality of life 31
9.5.4 Health economics 31
9.6 Planned subgroup analyses 31
9.7 Adjustments for covariates 31
9.7.1 Imputation of covariates 31
9.8 Interim analyses 31
10. Deviations from the protocol 32
10.1 Definition of analysis populations 32
10.2 Timing windows for follow-up visits 32
10.3 Analysis of secondary efficacy endpoints 32
10.3.1 Growth parameters and metabolism/vital signs 32
10.3.2 Gut dysbiosis on day 365 32
10.3.3 Development of immunological parameter during the first year 32
10.3.4 Postnatal exposure to antibiotics during primary hospital stay 32
10.4 Imputation of primary endpoint 32
10.5 Coding of SAEs 32
11. Interpretation of results 33
12. Further Analyses 33

13. Software 33
14. References 33
15. Appendices 34
15.1 Planned tables 34
15.1.1 Protocol deviations 34
15.1.2 Analysis of Intervention Group Comparability 35
15.1.2.1 Demographics and Baseline Characteristics 35
15.1.2.2 Prior or concomitant diseases and risk factors 37
15.1.3 Exposition to Treatment/Compliance 40
15.1.4 Primary Analyses 40
15.1.5 Secondary Analyses 41
15.1.5.1 Efficacy 41
15.1.5.1.1 Compound endpoint gut dysbiosis on day 31 41
15.1.5.1.2 Clinical sepsis during primary hospital stay 42
15.1.5.1.3 Blood-culture proven sepsis during primary hospital stay 42
15.1.5.1.4 Postnatal exposure to antibiotics during primary hospital stay 43
Daily dose per kilogram of body weight and Treatment with antibiotics per 1000 patient days 43
Number of antibiotic cycles 43
15.1.5.1.5 Infectious episodes in the first year of life 44
15.1.5.1.6 Wheezing episodes during the first year of life 44
15.1.5.1.7 Atopic dermatitis during the first year of life 45
15.1.5.1.8 Growth parameters 45
15.1.5.1.9 Nutritional aspects 46
15.1.5.1.10 Metabolism/Vital signs 46
15.1.5.2 Safety/Tolerability 46
15.1.5.2.1 Serious and non-serious events 46
15.2 Planned listings 50
15.2.1 Incomplete Treatments, Drop-Outs, Incomplete Observation, and Protocol Deviations 50
15.2.2 Subject disposition 52
15.3 Exposition to Treatment/Compliance 52
15.3.1 Secondary Analyses 53
15.3.1.1 Serious and non-serious adverse events 53
15.4 Planned figures 63
15.4.1 Subject disposition 63
15.5 Data needed for secondary efficacy endpoint “gut dysbiosis” 65

1. Aim

Central assumptions, methods and procedures for the statistical analysis are described in the study protocol. The statistical analysis plan (SAP) specifies in detail the statistical and biostatistical approaches and procedures to be used.

2. Responsibilities

The central data management (CDM) is carried out through the Institut für Medizinische Biometrie und Statistik and the Zentrum für Klinische Studien Lübeck, Universitätsklinikum Schleswig-Holstein, Campus Lübeck, Universität zu Lübeck.
The study data management (SDM) of the Institut für Medizinische Biometrie und Statistik and the Zentrum für Klinische Studien Lübeck, Universitätsklinikum Schleswig-Holstein, Campus Lübeck, Universität zu Lübeck receives the CRFS for double data entry into a database with audit trail for the data management for the interim and final analysis from the principal investigator according to the SOPs of the Zentrum für klinische Studien. From that point further processing of the data is subject to the SOP system of the Zentrum für Klinische Lübeck and the Institut für Medizinische Biometrie und Statistik, Universitätsklinikum Schleswig-Holstein, Campus Lübeck, Universität zu Lübeck.
Statistical analysis will be performed independently by the Institut für Medizinische Biometrie und Statistik, Universitätsklinikum Schleswig-Holstein, Campus Lübeck, Universität zu Lübeck. The SDM of the Institut für Medizinische Biometrie und Statistik und Zentrums für Klinische Studien, Universitätsklinikum Schleswig-Holstein, Campus Lübeck, Universität zu Lübeck provides the data for the interim and final analysis after query management according to the data validation plan (DVP) is completed. Discrepancies, which emerge retrospectively or are overlooked, complicate or distort the statistical analysis. To achieve high data quality, SDM and biostatisticians cooperate closely. Discrepancies, which may be discovered after database closure during statistical analysis, will be documented in the program code.
To prepare unblinded results in the interim analysis for the DSMB and the steering committee, an unblinded team consisting of a programmer and a study statistician will be installed. The unblinded programmer and study statistician will correspond with the DSMB about unblinded data and analyses. The steering committee will be provided with blinded data and analyses by the blinded team consisting of a programmer and a study statistician. Thus, blindness of all participating actors will be kept.

Contact / Biostatistician and programmer:
Institut für Medizinische Biometrie und Statistik
Universität zu Lübeck
Ratzeburger Allee 160, Haus 24
23562 Lübeck

Study statistician
Dr. Maren Vens
Tel.: +49 451 500 50622
m.vens@uni-luebeck.de

Unblinded study statistician for interim analysis
Nicole Hessler
Tel.: +49 451 500 50622
n.hessler@uni-luebeck.de

Independent statistician
Prof. Dr. rer. hum. biol. Inke. R. König
Tel.: +49 451 500 50610
inke.koenig@uni-luebeck.de

Programmer
Marina Bleskina
Tel.: +49 451 500 50612
marina.bleskina@uni-luebeck.de

Unblinded programmer for interim analysis
Frank Sandig
Tel.: +49 451 500 50612
frank.sandig@uni-luebeck.de

Preparation for analysis is performed by the SDM of the Institut für Medizinische Biometrie und Statistik and Zentrum für Klinische Studien, Universitätsklinikum Schleswig-Holstein, Campus Lübeck, Universität zu Lübeck. For interim analysis, the SDM hands over blinded data to the unblinded team who will prepare unblinded results for the interim analysis.

Contact / SDM:
Institut für Medizinische Biometrie und Statistik
Universität zu Lübeck
Ratzeburger Allee 160, Haus 24
23562 Lübeck

Marina Bleskina
Tel.: +49 451 500 50612
marina.bleskina@uni-luebeck.de

3. Study Design und Amendments

3.1 Study

Study design:	Prospective, randomized, controlled, multicenter, double blind
Study type:	Intervention study
Study phase:	IV
Randomization:	1:1
Number of participating sites:	18
Planned sample size:	Approximately 654
Registration of first patient:	12.04.2018
Planned study end (last patient in):	30.06.2020
Aim:	The primary objective is to define the potential of probiotics for modulating both the intestinal microbiota and the developing immune system in preterm infants. The primary aim of the clinical study is to determine the effect of Bifidobacterium infants/Lactobacillus acidophilus probiotics on the prevention of gut dysbiosis in preterm infants 28-32 weeks of gestation on day 28-30 of life.
Registration of the study:	DRKS00013197 (www.drks.de)

EudraCT-No.:	None

3.2 Study Amendments

3.2.1 Ethics committee

No	Application date	Description	Approval date
1	12.04.2017	Ethics approval multicenter study AZ 17-130	10.05.2017/17.07.17
2	01.02.2018	AZ 17-130 new version5_study protocol Version3_informed consent form	09.02.2018
3	29.09.2018	AZ 17-130 informed consent from german version 4 (+DGSVO_info)	04.10.2018
4	17.01.2019	AZ 17-130 Follow-up 1 year, blood withdrawal	25.01.2019
5	19.03.2019	AZ 17-130 informed consent from English version 4 (+DGSVO_info)	22.03.2019

3.2.2 BfArM

Not submitted to regulatory agencies.

4. Background

4.1 Trial Objective

The primary objective is to define the potential of probiotics for modulating both the intestinal microbiota and the developing immune system in preterm infants.
The primary aim of the clinical study is to determine the effect of Bifidobacterium infants/Lactobacillus acidophilus probiotics on the prevention of gut dysbiosis in preterm infants 28-32 weeks of gestation on day 28-30 of life.

4.2 Definitions

Terms that appear in this SAP and need to be addressed:

Term	Description
***Beispiel**: Richtig positiv*	*Der diagnostische Test ist positiv, und die Pathologie bestätigt den Krankheitsstatus „krank“*

4.3 Outcome Measures

4.3.1 Primary Outcome Measure

The primary efficacy outcome is gut dysbiosis at day 31 of life. The definition of gut dysbiosis is based on the guideline definitions of the KRINKO (Desphande G, 2016): Colonisation with MDRO or bacteria with epidemic potential (Enterobacter spp, Klebsielle spp, Serratia spp, Pseudomonas spp) as detected by microbiological culture. Thus, the primary endpoint is gut dysbiosis at day 31 and is assessed as the binary variable `t31_dysbiose`.

4.3.2 Secondary Outcome Measures

4.3.2.1 Efficacy

4.3.2.1.1 Gut dysbiosis

The main secondary efficacy outcome is gut dysbiosis at day 31. A compound definition of gut dysbiosis is based on

1. the guideline definitions of the KRINKO (Desphande G, 2016): Colonisation with MDRO or bacteria with epidemic potential (Enterobacter spp, Klebsielle spp, Serratia spp, Pseudomonas spp) as detected by microbiological culture or/and
2. significant deviations from the microbiome composition of healthy term infants (proportion of bacterial phyla, reduced diversity or specific features, such as increased virulence capacity and blooms of specific pathogens).

Clinical data as needed by European Molecular Biology Laboratory Heidelberg (Dr. Thea von Rossum, Prof. Dr. Peer Bork; PRIMAL Work Package 3 "Microbiome analysis by sequencing") for preparation of the second part of the compound endpoint (see Section 15.5) will be provided by the SDM.

Further efficacy outcomes are clinical or laboratory signs related to infection, immunity and metabolism.

4.3.2.1.2 Clinical or laboratory signs related to infection and secondary outcomes related to immunity

Clinical sepsis during primary hospital stay
Clinical sepsis is assessed using Neo-KISS criteria, e.g. at least five days of antibiotic therapy.

Blood-culture proven sepsis during primary hospital stay
Blood culture proven sepsis is clinical sepsis as defined above. Additionally, a pathogen has to be proven in blood-culture

Postnatal exposure to antibiotics during primary hospital stay
Postnatal exposure to antibiotics is assessed in a medication diary including course number, name of medication dose per kilogram of body weight and duration in days during primary hospital stay.

Infectious episodes in the first year of life
Infectious episodes are

- Fever episodes > 38°C rectal
- Common cold
- Bronchitis
- Pneumonia
- Gastroenteritis
- Urinary tract infections
- Others (for example otitis, tonsillitis).

and assessed via patient diaries, telephone interviews and personal interviews during follow-up visits.

Wheezing episodes
Wheezing episodes are assessed via patient diaries, telephone interviews and personal interviews during follow-up visits.

Atopic dermatitis
Atopic dermatitis is assessed via patient diaries, telephone interviews and personal interviews during follow-up visits.

4.3.2.1.3 Clinical or laboratory signs related to metabolism

Growth parameters
For analyses of growth parameters weight, head circumference and body length are assessed at baseline, day 28-30, release and 12-months-FU. Weight gain, velocity of growth for head circumference and body length during primary hospital stay and body weight, body length, head circumference at 12-months-FU are used as growth parameters.

Nutritional aspects
As nutritional aspects number of days to achieve full enteral feeding and forms of feeding are assessed during primary hospital stay.

Metabolism/Vital signs
As metabolism parameter blood pressure is assessed at 12-months-FU.

4.3.2.2 Safety/Tolerability

4.3.2.2.1 Extent of exposure

Extent of exposure is measured as days of drug administrations which are documentend by the study sites in the CRF and during drug accountability by the monitoring.

4.3.2.2.2 Serious and non-serious events

Safety outcome measures for this study are serious and non-serious adverse events during the primary hospital stay. SAEs of special interest are

- Blood-culture proven sepsis (for definition, see Chapter 4.3.2.1.2)
- Clinical sepsis (for definition, see Chapter 4.3.2.1.2)
- Necrotizing enterocolitis (NEC) ≥ Bell's stage 2
- Severe gastrointestinal complication (for example, bloody stool, surgery for focal intestinal perforation, volvulus, meconium obstruction syndrome)
- Death

4.3.2.2.3 Laboratory tests

Not assessed.

4.3.2.2.4 Vital signs

Not assessed.

4.3.2.3 Quality of Life

Not assessed.

4.3.2.4 Health economics

Not assessed.

5. Database

Database are the data double-entered into an ACCESS database according to SOPs of the Zentrum für klinische Studien Lübeck. In addition, attachments of monitoring reports containing information on protocol deviations and informed consents which are not GCP compliant will be attached to the database. Drug accountabilities prepared by the monitoring will be attached as a list to the database. Protocol deviations recorded as note to files will be handed over by the monitoring to the SDM group.
Database for statistical analyses are the data sets which are prepared and provided by the SDM group. These data sets have already completed the plausibility checks and query management according to the DVP. Data sets are corrected accordingly.
Every attribute, which is collected in the case report form (CRF), is declared as a variable. All variables are quoted in the annotated CRF. Data have to be extended, e.g. calculations, recodings or development of new variables which are necessary for the analyses.
The necessary extensions are described below in a data preparation and coding plan.

5.1 Incomplete Treatments, Drop-outs and Incomplete Observation

Information is documented in the CRF "Studienende"

- Lost to follow-up
- Patient withdrawal

As the primary endpoint of gut dysbiosis will be assessed and documented at the end of the initial hospital stay, it is not expected that there will be a relevant portion of patients with missing primary endpoint data.
Although all efforts are undertaken to follow up all patients who met at least one exclusion criterion and were still included in the study, it might not be possible to get complete observations from these patients. Project management, SDM and study statistician will list patients included in the study who meet at least one exclusion criterion prior to interim and final analyses. In each publication and DSMB it will be clearly stated
the number of patients not included in the primary analysis of data,
the circumstances under which patients were enrolled but excluded from the analysis.
For this purpose, a list will be provided by the SDM and study the statistician (see Listing 1, Listing 2 and Listing 3).
Reporting and visualization complies with the CONSORT guidelines.

5.2 Protocol Deviations

Protocol deviations will be recorded as note-to-files and the attachments of the monitoring reports and reviewed prior to database lock.

Deviations will be categorized into the following categories

- Randomization
- Inclusion/Exclusion criteria
- Informed consent form
- Performance of study procedure or visit
- Timing of window procedure or visit
- Compliance
- Other

Further protocol deviation categories may be identified during the study. Protocol deviations will be recorded and referenced to determine subjects to be excluded from the populations described in Chapter 7. The final decision regarding inclusion and exclusion of subjects from the analysis populations will be based on a listing of protocol deviations. This will be determined during a blinded data review meeting before database lock with input from Clinical and Biostatistics team members and approval from the sponsor (see Listing 1).
Protocol deviations will be summarized by type, major or minor, by site and by category for all enrolled subjects (see Table 4 and Table 5).

Time windows for data acquisition at each study visit are shown in Table 1.

Table 1 Time windows for data acquisition at each study visit

Visit	Acceptable time window
Baseline & Randomization	+ 48 hours
31 days follow-up (after randomization, Primärer Endpunkt)	+ 14 days to + 35 days after randomization
6 and 12 months follow-up	1) Until 04.04.2019 (date of 6- and 12-months FU, respectively) time between randomization and 6- and 12-months FU: 6 months± 2 weeks and 12 months ± 2 weeks, respectively<br>2) Between 05.04.2019 and 01.03.2020 (date of 6- and 12-months FU, respectively) time between randomization and 6- and 12-months FU: (corrected age = biological age – (40 weeks – gestational weeks) ± 2 weeks

Visit	Acceptable time window
	and (corrected age = biological age – (40 weeks – gestational weeks) ± 2 weeks, respectively<br>3) Since 02.03.2020 (date of 6- and 12-months FU, respectively) time between randomization and 6- and 12-months FU: (corrected age = biological age – (40 weeks – gestational weeks) - 2 weeks until 6 months + 8 weeks and (corrected age = biological age – (40 weeks – gestational weeks) - 2 weeks until 12 months + 8 weeks, respectively
SAE	+ 24 hours after detection

Protocol deviations will be listed as treated in Group 1: Verum, in Group 2: Placebo (see Chapter 7.5)

6. Analysis Timing

The interim analyses will be performed after 322 preterm infants reached the day 28-30 visit. The cut-off date will be the date of the 322th preterm infant's day 28-30 visit. Thus, the interim analysis will be performed on the data collected until the cut-off date. All data after the cut-off date will be excluded from the analysis. All tables, listings and figures detailed in Chapters 9.1, 9.2, 9.3, 9.4, 9.5.2, 9.7 and 9.8 will be produced for this analysis. The analysis cut-off date will be applied to remove from the analysis any of the following records:

- Any individual record for patients who have an informed consent signature date after the cut-off date.
- Any protocol deviation that occurred after the cut-off date.
- Any assessment or evaluation that is performed after the cut-off date.
- Any adverse event, medication, procedure and treatment administration that is started after the cut-off date.

In all domains (in particular for adverse events and medications) where a start date and an end date are collected, any end date after the cut-off date will not be imputed (i.e. an AE that ended after the cut-off date but started before will be kept as it is). Interim analysis will only be performed for the primary endpoint as described in sections 9.4 and 9.8 on the ITT population without any sensitivity analysis or imputation.

DSMB will be scheduled after the 322th preterm infant's day 31 visit for the review of the safety data and the results of the interim analysis.

Main analysis will be performed after the day 31 visit of the all patients included in the study. Thus, the main analysis will be performed on the data collected until the 654th preterm infant's day 31 visit. All data after the cut-off date will be excluded from the analysis. All tables, listings and figures detailed Chapter 9 will be produced for this analysis. The analysis cut-off date will be applied to remove from the analysis any of the following records:

- Any protocol deviation that occurred after the cut-off date.
- Any assessment or evaluation that is performed after the cut-off date.
- Any adverse event, medication, procedure and treatment administration that is started after the cut-off date.

In all domains (in particular for adverse events and medications) where a start date and an end date are collected, any end date after the cut-off date will not be imputed (i.e. an AE that ended after the cut-off date but started before will be kept as it is).
Main analysis of primary and secondary endpoints will only be performed on day 31 visit data.

Final analysis will be performed after the end of study, i.e.

- when all patients are followed up for at least 12 months or
- the DSMB gives a formal recommendation to stop the trial, e.g. if
  - a difference in the primary outcome measure is observed between treatment groups in the interim analysis according to predefined rules (see Chapter 9.8)
  - a difference in serious events is observed between treatment groups in the interim analysis,
  - the evaluation of safety reports shows that the risk-benefit ratio is no longer considered acceptable,
  - results from other studies show benefit or harm with any of the interventions and the risk-benefit ratio is no longer considered acceptable.

All tables, listings and figures detailed in the next sections and according to Chapter 9 provided for earlier analyses will be repeated for final analysis.

7. Sets of Patients

7.1 Safety Analysis (SA) Set

Implementation: no ○ yes ●

This analysis set is used for the analysis of adverse events and every variable presenting safety and tolerability of the examined interventions.

Patients

- with invalid written informed consent
- who withdrew their informed consent by a legally authorized representative and demanded the complete deletion of the patient's data
- who were not randomized
- without any drug administration

will be excluded from this data set. Patients will be analyzed as treated.

7.2 Intention-To-Treat (ITT) Set

Implementation: no ○ yes ●

This analysis set comprises all randomized patients including all patients with minor and major protocol deviations (see Chapter 5.2) and all patients with missing values.

Patients

- with invalid written informed consent
- who withdrew their informed consent by a legally authorized representative and thereby demanded the complete deletion of the patient's data
- who were not randomized

will be excluded from this data set for the respective analysis. Patients will be analyzed as randomized.

7.3 Full Analysis (FA) Set

Implementation: no ● yes ○

Patients in the ITT set will be excluded from the analysis, if at least one of the following criteria is met:

- Violation of at least one inclusion or exclusion criterion
- No data available after randomization

7.4 Per Protocol (PP) Set

Implementation: no ○ yes ●

This data set includes all patients who were treated as described in the protocol.

Patients

- with invalid written informed consent
- with violation of at least one inclusion or exclusion criterion
- with major protocol violations except for major violations concerning the written informed consent which were solved during the course of the study

- who withdrew their informed consent by a legally authorized representative
- who were not randomized
- without any data after randomization
- with less than 14 days of uninterrupted intervention or with more than 72h not administered intervention

will be excluded from this data set for the respective analysis.

7.5 As Treated (AT) Set

Implementation: no ◯ yes ⬤

The AT set consists of the same patients as in the ITT set. Patients will be analyzed as treated.

7.6 Application of Analysis Sets

Table 2 Application of Analysis Sets. I: Interim Analysis, M: Main Analysis, F: Final analysis, SM: Sensitivity Analysis at main analysis, SF: Sensitivity analysis at final analysis

	Analysis Set				
**Outcome**	**ITT**	**PP**	**FA**	**AT**	**SA**
**Primary Outcome**<br>Gut dysbiosis at day 31	I, M, F	SM, FM		SM, FM	
**Secondary Outcomes**					
**Efficacy**					
Gut dysbiosis at day 31	M, F	SM, FM		SM, FM	
Clinical or laboratory signs related to Infection and secondary outcomes related to immunity	M, F	SM, FM		SM, FM	
Clinical or laboratory signs related to metabolism	M, F	SM, FM		SM, FM	
**Safety/Tolerability**					
AEs/SAEs					I, M, F
**Quality of life**	Not assessed				
**Health economics**	Not assessed				

8. Handling of Missing Values, Missing Data and Outliers

8.1 Missing values, Missing Data

Imputation will be carried out in the following variables which are needed for calculation of the primary endpoint or corresponding analyses:

- `t31_dysbiose`

If variables for calculation of endpoints or covariates contain >25% missing values, these variables and all subsequently calculated variables are used in univariate description only without imputation.

Imputed data sets will be labelled with suffix “-imp”, e.g. ITT-imp and used for sensitivity analyses. Imputation will be carried out on the ITT data set and subsequent data sets will be built depending on the ITT data set.

Summary of imputation is given in Table 3. More detailed description for every imputed variable can be found in the imputation sections in Chapter 9,

Table 3 Summary of imputation in this study

Imputation method	Condition	Relevant response variables
■ None*	Interim analysis	All variables needed for primary analysis and covariates
	Main and final analyses	All variables needed for primary analyses and covariates with > 25% missing values in the complete data set
☐ Mean		
☐ Median		
☐ Worst Observation Carried Forward (WOCF)		
☐ Best Observation Carried Forward (BOCF)		
☐ Last-Observation-Carried-Forward (LOCF)		
☐ Baseline Observation Carried Forward (BOCF)		
☐ Regression methods		
☐ Multiple Imputation using mice (multivariate imputation by chain equations)		
■ Other	Main and final analysis	`t31_dysbiose`

*Es wird von einem vollständigen zufälligen Fehlen (missing completely at random) von Messwerten ausgegangen und dieses wird vernachlässigt.
**Genaue Methode der multiplen Imputation muss spezifiziert werden.

8.2 Outliers

Outliers in metric variables, especially those influencing the primary and secondary endpoints, will be avoided using warnings if values out of range are entered in the CRF. Additionally, query management is performed by the SDM group.

9. Statistical analyses/methods

9.1 Subject disposition

There will be a clear accounting of all patients who entered the study using tables and figures. The numbers of patients who were randomized, and who entered and completed each phase of the study will be provided as well as the reasons for all post-randomization discontinuations, grouped by treatment and by major reason (e.g. lost-to-follow-up, adverse event, poor compliance, withdrawals).

A flow chart according to CONSORT statement will be prepared (see Figure 1 and a blinded version for interim analysis see Figure 2). Whether patients were followed up for the duration of the study even after discontinuation, should be made clear.

A listing of all patients discontinued from the study after randomization, broken down by centre and treatment group, giving a patient identifier, the specific reason for discontinuation, as well as last visit will be given (see Listing 3).

9.2 Analysis of Intervention Group Comparability

The following information will be summarized by intervention groups on ITT population at interim analysis and on ITT, PP and AT populations at main and final analyses.

9.2.1 Demographics and Baseline Characteristics

- Birth characteristics (see Table 6)
    - Multiple birth
    - Delivery
        - Spontaneous
        - Caesarean section
        - Emergency caesarean section
            - If caesarean or emergency caesarean section
                - Uterus without labor
    - Antenatal steroids
        - If yes, completed cycle (2 doses, 12 h after 2nd dose)
    - Rupture of membranes before labor
- Demographics of preterm infants (see Table 7 and Table 8)
    - Sex
    - Birth weight (in g)
    - Body length (in cm)
    - Temperature (in °C)
    - Head circumference (in cm)
    - Gestational age (in GW)
- Early Bonding/Microbiome (see Table 9 and Table 10)
    - Bonding in delivery room
        - If yes, mouth to breast contact, non-nutritive sucking
    - First kangorooing
        - If yes, day of life
    - Seeding
    - Mothers hospital stay > 1 week before delivery*
      * Only in mothers with informed consent version 4 or higher

9.2.2 Prior or concomitant diseases and risk factors

Known comorbidities from clinical history, medication or other treatment will be summarized as follows:

- Cause of preterm delivery (see Table 11)
    - Preterm labor
    - Pathological cardiotocography
    - amniotic infection syndrome (suspected or proven)
    - Intrauterine growth restriction, pathological doppler
    - Pre-eclampsia
    - Placenta abruption
    - HELLP-syndrome
    - Rupture of membranes > 5 days of delivery, anhydramnios
    - Prolapse of membranes
    - Rupture of membranes without anhydramnios
    - Other
- Mothers data (only for mothers with written informed consent version 04 or higher, see Table 12 and Table 13)
    - Age (in years)
    - Body weight at diagnosis of pregnancy (in kg)
    - Body length (in cm)
    - Blood group (in categories: A, B, AB, O)
    - Former pregnancies/deliveries
        - If yes
            - Number of pregnancies (without actual delivery)
            - Number of deliveries (without actual delivery)
            - Preterm deliveries before 37+0 GW (without actual delivery)

9.2.3 Prior or concomitant medication

Antenatal antibiotics will be summarized as follows (see also Table 14):

- Antenatal antibiotics (only for mothers with written informed consent version 04 or higher)
    - If yes until 2 months before delivery
        - Ampicillin or other penicillins
        - Cephalosorins
        - Carbapenems
        - Metronidazol
        - Macrolides
        - Other

9.3 Exposition to treatment/Compliance

Exposition to intervention and treatment compliance will be listed by patient displaying the length of primary hospital stay, the number of days without medication between randomization and primary endpoint as documented in the CRF and the drug accountability performed during the monitoring visit (see Listing 4). Additionally, descriptives will be tabulated as in Table 15.

All protocol deviations concerning delayed or missing medication as well as drug accountability will be listed as described in Section 5.2. There will be one listing summarizing protocol deviations concerning exposition to intervention and treatment compliance (see Listing 5).

9.4 Primary Analyses

Analysis of primary endpoint will be conducted on ITT population and presented by treatment arms, unless otherwise stated. Significance levels and stopping boundaries for interim analysis as well as significance level for mail analysis will be calculated as described in Chapter 9.8. There will be no multiplicity adjustments for testing of other secondary endpoints, unless otherwise stated.

The primary aim of the clinical study is to determine the effect of Bifidobacterium infants/Lactobacillus acidophilus probiotics on the prevention of gut dysbiosis in preterm infants 28-32 weeks of gestation on day 31 of life.

The null and alternative hypotheses for no-difference between verum and placebo are as follows:
H_0: OR=1 vs. H_1: OR ≠ 1,
in which OR is the odds ratio gut dysbiosis at day 31 of the verum group over the placebo group.

The main analysis of gut dysbiosis on day 31 will be a generalized linear mixed effects model. Sex is used as a fixed, and study site and gestational age in two categories as used for randomization as random effects. To test the treatment effect, a Wald test for the log OR will be used. Corresponding ($1-\alpha_a$)- CI, with a is either interim or final analysis, and 95%-CI will be given.

The primary endpoint is the binary gut dysbiosis on day 31, the corresponding variable is `t31_dysbiose` from the discharge CRF. A 2x2 table between treatment and gut dysbiosis on day 31 and results from the generalized linear mixed effect will be reported as shown in Table 16 and Table 17.

The following SAS Code will be used for the generalized linear mixed model:

```
PROC GLIMMIX data = data;
      class zid week bl_geschlecht Treatment;
```

```
      model endpoint (event = LAST) = Treatment bl_geschlecht /dist binary
link = logit oddsratio solution;
      random intercept /subject = zid;
      random intercept / subject = week(zid);
      estimate 'Verum vs. Placebo' treatment -1 1 /alpha=.05 exp;
      estimate 'Verum vs. Placebo' treatment -1 1 /alpha =.012 exp;
      * select alpha depending on analysis
      ods select estimates;
run;
```

9.4.1 Analysis assumptions and alternative analyses

In case of stability problems because number of cases or controls is too small (less than 30), study site will be eliminated from the model.

9.4.2 Imputation

Missing values of the primary endpoint will be imputed using the values provided by the EMBL (`t31_devmicro`), see Chapter 9.5.1.1.

9.4.3 Sensitivity analysis

As sensitivity analyses, the analyses of gut dysbiosis as described above will be performed using the ITT-imp, PP and AT data sets.

9.5 Secondary analyses

Except for the primary analyses there are no explicit or implied hypotheses in the protocol. Changes in the secondary outcomes are of interest as they will relate to the efficacy as measured by the primary outcome variable. All analyses are descriptive and are intended to document the changes in these important outcomes. There will be no multiplicity adjustments for testing of secondary endpoints, unless otherwise stated. P-Values from secondary endpoints will only be estimated for exploratory analysis. Secondary outcomes will be used to support the decision for efficacy of probiotics but are not intended to be used explicitly for making a decision for the efficacy probiotics in this population.

Analysis of secondary efficacy endpoints will be conducted on ITT population and presented by treatment arms, unless otherwise stated. Analysis of safety/tolerability endpoints will be conducted on the SA data set and presented by treatment arms, unless otherwise stated.

9.5.1 Efficacy

9.5.1.1 Gut Dysbiosis

The main secondary efficacy endpoint is gut dysbiosis at day 31, which is assessed as a compound endpoint (`sect31_dysbiose`). This compound endpoint is either yes (1) if `t31_dysbiose` has the value 1 (yes) or there are significant deviations from the microbiome of healthy term infants (variable `t31_devmicro` provided by the EMBL Heidelberg has value 1 (yes)).

Analysis of this endpoint will be a generalized linear mixed effects model. Sex is used as a fixed, and study site and gestational age in two categories as used for randomization as random effects. To test the treatment effect, a Wald test for the log OR will be used. The corresponding 95%-CI will be given.

A 2x2 table between treatment and gut dysbiosis on day 31 and results from the generalized linear mixed effect will be reported as shown in Table 18 and Table 19.

The following SAS Code will be used for the generalized linear mixed model:

```
PROC GLIMMIX data = data;
      class zid week bl_geschlecht Treatment;
```

```
      model sect31_dysbiose (event = LAST) = Treatment bl_geschlecht /dist
binary link = logit oddsratio solution;
      random intercept /subject = zid;
      random intercept / subject = week(zid);
      estimate 'Verum vs. Placebo' treatment -1 1 /alpha=.05 exp;
      estimate 'Verum vs. Placebo' treatment -1 1 /alpha =.012 exp;
      * select alpha depending on analysis
      ods select estimates;
run;
```

9.5.1.1.1 Analysis assumptions and alternative analyses

In case of stability problems because number of cases or controls is too small (less than 30), study site will be eliminated from the model.

9.5.1.1.2 Sensitivity analysis

As sensitivity analyses, the analyses of gut dysbiosis as described above will be performed using the ITT-imp, PP and AT data sets.

9.5.1.2 Clinical or laboratory signs related to infection and secondary endpoints related to immunity

9.5.1.2.1 Clinical sepsis during primary hospital stay

Clinical sepsis is assessed using Neo-KISS criteria, e.g. at least five days of antibiotic therapy. The corresponding binary endpoint is assessed on discharge CRF as variable `ent_sepsis`. Analysis of this endpoint will be a generalized linear mixed effects model. Sex is used as a fixed, and study site and gestational age in two categories as used for randomization as random effects. To test the treatment effect, a Wald test for the log OR will be used. The corresponding 95%-CI will be given.

A 2x2 table between treatment and clinical sepsis during primary hospital stay and results from the generalized linear mixed effect will be reported as shown in Table 20 and Table 21.
The following SAS Code will be used for the generalized linear mixed model:

```
PROC GLIMMIX data = data;
      class zid week bl_geschlecht Treatment;
      model endpoint (event = LAST) = Treatment bl_geschlecht /dist binary
link = logit oddsratio solution;
      random intercept /subject = zid;
      random intercept / subject = week(zid);
      estimate 'Verum vs. Placebo' treatment -1 1 /alpha=.05 exp;
      ods select estimates;
run;
```

Analysis assumptions and alternative analyses
In case of stability problems because number of cases or controls is too small (less than 30), study site will be eliminated from the model.

Sensitivity analysis
As sensitivity analyses, the analyses of clinical sepsis during primary hospital stay as described above will be performed using the ITT-imp, PP and AT data sets.

9.5.1.2.2 Blood-culture proven sepsis during primary hospital stay

Blood culture proven sepsis is clinical sepsis as defined above. Additionally, a pathogen has to be proven in blood-culture. This is documented on the discharge CRF. The endpoint to be evaluated is `sec_sepsis_blut_err` and is calculated using variables `ent_sepsis` and `ent_sepsis_blut_err`.

Analysis of this endpoint will be a generalized linear mixed effects model. Sex is used as a fixed, and study site and gestational age in two categories as used for randomization as random effects. To test the treatment effect, a Wald test for the log OR will be used. The corresponding 95%-CI will be given.

A 2x2 table between treatment and blood-culture proven sepsis during primary hospital stay and results from the generalized linear mixed effect will be reported as shown in Table 22 and 23.

The following SAS Code will be used for the generalized linear mixed model:
```
PROC GLIMMIX data = data;
      class zid week bl_geschlecht Treatment;
      model endpoint (event = LAST) = Treatment bl_geschlecht /dist binary
link = logit oddsratio solution;
      random intercept /subject = zid;
      random intercept / subject = week(zid);
      estimate 'Verum vs. Placebo' treatment -1 1 /alpha=.05 exp;
      estimate 'Verum vs. Placebo' treatment -1 1 /alpha =.012 exp;
      * select alpha depending on analysis
      ods select estimates;
run;
```

Analysis assumptions and alternative analyses
In case of stability problems because number of cases or controls is too small (less than 30), study site will be eliminated from the model.

Sensitivity analysis
As sensitivity analyses, the analyses of blood culture proven sepsis as described above will be performed using the ITT-imp, PP and AT data sets.

9.5.1.2.3 Postnatal exposure to antibiotics during primary hospital stay

Postnatal exposure to antibiotics is assessed in a medication diary including course number, name of medication dose per kilogram of body weight and duration in days during primary hospital stay.
This outcome is splitted into three endpoints of those each is analyzed separately:

- Daily dose per kilogram of body weight
- Treatment with antibiotics per 1000 patient days
- Number of antibiotic cycles

Daily dose per kilogram of body weight and treatment with antibiotics per 1000 patient days
Daily dose per kilogram of body weight and treatment with antibiotics per 1000 patient days will both be analyzed by type of antibiotic and treatment group. Descriptive statistics for daily dose per kilogram body weight will be given as in Table 24. Treatment with antibiotics per 1000 patient days will be calculated as
Sum of treatment days with antibiotics/(sum of patient days * 1000) per group and reported as in Table 24.

Number of antibiotic cycles
Number of antibiotic cycles are counted using course numbers in the day 31 CRF. The maximum course number is stored in the new variable `sec_no_anti`.
Analysis of this endpoint will be a generalized linear mixed effects model using a log linear linear poisson model. Sex is used as a fixed, and study site and gestational age in two categories as used for randomization as random effects. To test the treatment effect, a Wald test for the log OR will be used. The corresponding 95%-CI will be given.
Results from the generalized linear mixed effect will be reported as shown in Table 25.
The following SAS Code will be used for the generalized linear mixed model:

```
proc glimmix data=counts method=quad;
      class zid week bl_geschlecht Treatment;
      model sec_no_anti = Treatment bl_geschlecht / link=log s
dist=poisson;
      random intercept /subject = zid;
      random intercept / subject = week(zid);
run;
```

Sensitivity analysis
As sensitivity analyses, the analyses of all endpoints described above will be performed using the ITT-imp, PP and AT data sets.

9.5.1.2.4 Infectious episodes in the first year of life

Fever episodes > 38°C rectal are assessed in follow-ups at 6 and 12 months, variables are `fu6_fieber` and `fu12_fieber`. These variables will be summarized to one variable `sec_fieber` which has value 1 if either `fu6_fieber` or `fu12_fieber` is 1 and else 0.

Common cold is assessed in follow-ups at 6 and 12 months, variables are `fu6_gripp` and `fu12_gripp`. These variables will be summarized to one variable `sec_gripp` which has value 1 if either `fu6_gripp` or `fu12_gripp` is 1 and else 0.

Bronchitis is assessed in follow-ups at 6 and 12 months, variables are `fu6_bronchitis` and `fu12_bronchitis`. These variables will be summarized to one variable `sec_bronchitis` which has value 1 if either `fu6_bronchitis` or `fu12_bronchitis` is 1 and else 0.

Pneumonia is assessed in follow-ups at 6 and 12 months, variables are `fu6_lungenentz` and `fu12_lungenentz`. These variables will be summarized to one variable `sec_lungenentz` which has value 1 if either `fu6_lungenentz` or `fu12_lungenentz` is 1 and else 0.

Gastroenteritis is assessed in follow-ups at 6 and 12 months, variables are `fu6_magen_darm` and `fu12_magen_darm`. These variables will be summarized to one variable `sec_magen_darm` which has value 1 if either `fu6_magen_darm` or `fu12_magen_darm` is 1 and else 0.

Urinary tract infections are assessed in follow-ups at 6 and 12 months, variables are `fu6_harnweg` and `fu12_harnweg`. These variables will be summarized to one variable `sec_harnweg` which has value 1 if either `fu6_harnweg` or `fu12_harnweg` is 1 and else 0.

Other infections (for example otitis, tonsillitis) are assessed in follow-ups at 6 and 12 months, variables are `fu6_weitere_infekt` and `fu12_weitere_infekt`. These variables will be summarized to one variable `sec_weitere_infekt` which has value 1 if either `fu6_weitere_infekt` or `fu12_weitere_infekt` is 1 and else 0.

Analysis of all of the endpoints described above will be a generalized linear mixed effects model. Sex is used as a fixed, and study site and gestational age in two categories as used for randomization as random effects. To test the treatment effect, a Wald test for the log OR will be used. The corresponding 95%-CI will be given.

For all of the endpoints described above crosstables between treatment and the respective endpoint and results from the generalized linear mixed effect will be reported as shown in Table 26 and Table 27.

The following SAS Code will be used for the generalized linear mixed model:

```
PROC GLIMMIX data = data;
      class zid week bl_geschlecht Treatment;
      model endpoint (event = LAST) = Treatment bl_geschlecht /dist binary
link = logit oddsratio solution;
      random intercept /subject = zid;
      random intercept / subject = week(zid);
      estimate 'Verum vs. Placebo' treatment -1 1 /alpha=.05 exp;
      ods select estimates;
run;
```

Analysis assumptions and alternative analyses
In case of stability problems because number of cases or controls is too small (less than 30), study site will be eliminated from the model.

Sensitivity analysis
As sensitivity analyses, the analyses of all endpoints from this chapter as described above will be performed using the ITT-imp, PP and AT data sets.

9.5.1.2.5 Wheezing episodes during the first year of life

Wheezing episodes are assessed in follow-ups at 6 and 12 months, variables are `fu6_pfeifend` and `fu12_pfeifend`. These variables will be summarized to one variable `sec_pfeifend` which has value 1 if either `fu6_pfeifend` or `fu12_pfeifend` is 1 and else 0.

Analysis of this endpoint will be a generalized linear mixed effects model. Sex is used as a fixed, and study site and gestational age in two categories as used for randomization as random effects. To test the treatment effect, a Wald test for the log OR will be used. The corresponding 95%-CI will be given.

A 2x2 table between treatment and wheezing episodes and results from the generalized linear mixed effect model will be reported as shown in Table 28 and Table 29.

The following SAS Code will be used for the generalized linear mixed model:

```
PROC GLIMMIX data = data;
      class zid week bl_geschlecht Treatment;
      model endpoint (event = LAST) = Treatment bl_geschlecht /dist binary
link = logit oddsratio solution;
      random intercept /subject = zid;
      random intercept / subject = week(zid);
      estimate 'Verum vs. Placebo' treatment -1 1 /alpha=.05 exp;
      ods select estimates;
run;
```

Analysis assumptions and alternative analyses
In case of stability problems because number of cases or controls is too small (less than 30), study site will be eliminated from the model.

Sensitivity analysis
As sensitivity analyses, the analyses of wheezing episodes as described above will be performed using the ITT-imp, PP and AT data sets.

9.5.1.2.6 Atopic dermatitis during the first year of life

Atopic dermatitis is assessed in follow-ups at 6 and 12 months, variables are `fu6_haut` and `fu12_haut`. These variables will be summarized to one variable `sec_haut` which has value 1 if either `fu6_haut` or `fu12_haut` is 1 and else 0.

Analysis of this endpoint will be a generalized linear mixed effects model. Sex is used as a fixed, and study site and gestational age in two categories as used for randomization as random effects. To test the treatment effect, a Wald test for the log OR will be used. The corresponding 95%-CI will be given.

A 2x2 table between treatment and wheezing episodes and results from the generalized linear mixed effect model will be reported as shown in Table 30 and Table 31.

The following SAS Code will be used for the generalized linear mixed model:

```
PROC GLIMMIX data = data;
      class zid week bl_geschlecht Treatment;
      model endpoint (event = LAST) = Treatment bl_geschlecht /dist binary
link = logit oddsratio solution;
      random intercept /subject = zid;
      random intercept / subject = week(zid);
      estimate 'Verum vs. Placebo' treatment -1 1 /alpha=.05 exp;
      ods select estimates;
run;
```

Analysis assumptions and alternative analyses
In case of stability problems because number of cases or controls is too small (less than 30), study site will be eliminated from the model.

Sensitivity analysis
As sensitivity analyses, the analyses of wheezing episodes as described above will be performed using the ITT-imp, PP and AT data sets.

9.5.1.3 Clinical or laboratory signs related to metabolism

9.5.1.3.1 Growth parameters

Weight gain during primary hospital stay (`sec_wg`) is calculated using the difference of weight at day 31 (`t31_gewicht`) and baseline (`bl_gewicht`) devided by length of hospital stay (`t31_dat-bl_gebdat`).

Velocity of growth for head circumference during primary hospital stay (`sec_velhead`) is defined as difference in head circumference between day 31 (`t31_kopf`) and baseline (`bl_kopf`) devided by length of hospital stay (`t31_dat - bl_gebdat`).

Velocity of body length during primary hospital stay (`sec_vellength`) is defined as difference in body length between day 31 (`t31_laenge`) and baseline (`bl_laenge`) devided by length of hospital stay (`t31_dat - bl_gebdat`).

Endpoints `sec_wg`, `sec_velhead` and `sec_vellength` will be analyzed using a linear mixed model. Sex is used as a fixed, and study site and gestational age in two categories as used for randomization as random effects. The respective models will be adjusted for baseline values. Results will be summarized as shown in Table 32.

The following SAS Code will be used for these endpoints:

```
PROC MIXED
      class zid week bl_geschlecht Treatment;
      model sec_ep = bl_value Treatment bl_geschlecht
      random zid week;
run;
* Additional parameters may be added;
```

In 12-months FU endpoints body weight (`fu12_heute_gewicht`), body length (`fu12_heute_laenge`) and head circumference (`fu12_heute_kopf`) are assessed.

Endpoints fu12_heute_gewicht, fu12_heute_laenge and fu12_heute_kopf will be analyzed using linear mixed models. Sex is used as a fixed, and study site and gestational age in two categories as used for randomization as random effects. Results will be summarized as shown in Table 32. The following SAS Code will be used for every endpoint:

```
PROC MIXED
      class zid week bl_geschlecht Treatment;
      model sec_ep = Treatment bl_geschlecht lifetime
lifetime*treatment;
      random zid week;
run;
* Additional parameters may be added;
* lifetime is time between birth and 12- months FU in days;
```

Analyses assumptions and alternative analyses
Assumptions are normality of residuals, which is evaluated using a quantile-quantile plot for the residuals, collinearity of independent variables, which is evaluated using the covariance structure of the independent variables, homoscedasticity, which is evaluated in a scale-location plot. Furthermore, a Tuckey-Anscombe-Plot is used to evaluate the propriety of the model. Also, the plot shows whether there is a systematic error.
If normality of residuals is violated or errors are heteroscedastic, the outcome will be transformed e.g. by using the logarithm or squared outcome. If collinearity is shown, independent variables should be summarized or left out.

Sensitivity analysis
As sensitivity analyses, the analyses for all endpoints frim this chapter as described above will be performed using the ITT-imp, PP and AT data sets.

9.5.1.3.2 Nutritional aspects
Forms of feeding during primary hospital stay are assessed as binary variables in the discharge CRF. Forms of feeding are breast milk (ent_milch) and formula nutrition (ent_forumula).

Analysis of ent_milch and ent_formula will be a generalized linear mixed effects model each. Sex is used as a fixed, and study site and gestational age in two categories as used for randomization as random effects. To test the treatment effect, a Wald test for the log OR will be used. The corresponding 95%-CI will be given.

Crosstables between treatment and the respective form of feeding during primary hospital stay and results from the generalized linear mixed effect will be reported as shown in Table 33 and Table 34.

The following SAS Code will be used for the generalized linear mixed model:

```
PROC GLIMMIX data = data;
      class zid week bl_geschlecht Treatment;
      model endpoint (event = LAST) = Treatment bl_geschlecht /dist binary
link = logit oddsratio solution;
      random intercept /subject = zid;
      random intercept / subject = week(zid);
      estimate 'Verum vs. Placebo' treatment -1 1 /alpha=.05 exp;
      ods select estimates;
run;
```

Analysis assumptions and alternative analyses
In case of stability problems because number of cases or controls is too small (less than 30), study site will be eliminated from the model.

Sensitivity analysis
As sensitivity analyses, the analyses of all endpoints from this chapter as described above will be performed using the ITT-imp, PP and AT data sets.

9.5.1.3.3 Metabolism/Vital signs

Diastolic and systolic blood pressure are assessed at 12-months-FU in variables `fu12_ blutdruck_diast` and `fu_12_ blutdruck_sys`. Endpoints `fu12_ blutdruck_diast` and `fu_12_ blutdruck_sys` will be analyzed using linear mixed models. Sex is used as a fixed, and study site and gestational age in two categories as used for randomization as random effects. Results will be summarized as shown in Table 35.

The following SAS Code will be used for every endpoint:

```
PROC MIXED
      class zid week bl_geschlecht Treatment;
      model sec_ep = Treatment bl_geschlecht lifetime
lifetime*treatment;
      random zid week;
run;
* Additional parameters may be added;
* lifetime is time between birth and 12- months FU in days;
```

Analyses assumptions and alternative analyses
Assumptions are normality of residuals, which is evaluated using a quantile-quantile plot for the residuals, collinearity of independent variables, which is evaluated using the covariance structure of the independent variables, homoscedasticity, which is evaluated in a scale-location plot. Furthermore, a Tuckey-Anscombe-Plot is used to evaluate the propriety of the model. Also, the plot shows whether there is a systematic error.
If normality of residuals is violated or errors are heteroscedastic, the outcome will be transformed e.g. by using the logarithm or squared outcome. If collinearity is shown, independent variables should be summarized or left out.

Sensitivity analysis
As sensitivity analyses, the analyses for all endpoints frim this chapter as described above will be performed using the ITT-imp, PP and AT data sets.

9.5.2 Safety/Tolerability

Safety and tolerability will be analyzed on the SA data set.

9.5.2.1 Extent of exposure

Extent of exposure is measured as days with drug administration and documented by the study sites on the CRF and during drug accountability by the monitoring and analyzed together with compliance (for details see Chapter 9.3).

9.5.2.2 Serious and non-serious adverse events

All adverse events occurring after randomization will be displayed in summary tables.

AEs and SAEs will be coded by the SDM group using MedDRA.
The following characteristics will be presented in the safety report:

- Number of AEs
- Median time of AEs in days
- Number of SAEs as documented in the AE CRF
- Median time of SAEs in days as documented in the AE CRF
- Number of SAEs as documented in the SAE CRF
- Median time of SAEs in days as documented in the SAE CRF

Characteristics documented in the AE CRF will be presented for each treatment group and the complete safety data set together with severities, causal relationships and status/outcome of serious and non-serious events (see Table 36). Relationship was assessed as definite, probable, possible, unlikely, no and unknown relationship. According to ICH Guidelines, relationship will be summarized into two categories: Related (definite, probable, possible and unknown relationship) and not related (unlikely and no relationship).

Characteristics documented in the SAE CRF will be presented for each treatment group and the complete safety data set (see Table 37). Here, relationship is summarized into the two categories as follows: Not related (no relationship) and related (all other possibilities).

All adverse events for each patient should be listed giving both preferred term and the original term used by the investigator. The listing should be by center and by treatment and should include (see Listing 6):

- Patient ID
- Age at onset of AE, sex, weight, height
- Description of adverse event (SOC, HLGT, HLT, PT, LLT, reported term)
- Date of onset or date of clinic visit at which the event was discovered
- Duration of verum/placebo treatment until date of onset or date of clinic visit at which the event was discovered
- Duration of the AE
- Severity (mild, moderate, severe, life-threatening consequences, Death related to AE)
- Causal relationship to the treatment (no relationship, unlikely relationship, possible relationship, probable relationship, definite relationship, unknown)
- Action taken
- Outcome (Recovered; Recovering; Not yet recovered, further treatment not necessary; Not yet recovered, further treatment not necessary; Fatal; unknown)
- Seriousness (serious/non-serious)

For all serious events the following will be listed additionally (see Listing 7):

- Patient ID
- Age at onset of SAE, sex, weight, height
- Description of serious adverse event (SOC, HLGT, HLT, PT, LLT, reported term)
- Type of SAE of special interest (Blood-culture proven sepsis, clinical sepsis, necrotizing enterocolitis (NEC) ≥ Bell's stage 2, severe gastrointestinal complication)
- Date of onset or date of clinic visit at which the event was discovered
- Duration of verum/placebo treatment until date of onset or date of clinic visit at which the event was discovered
- Duration of the SAE
- CTCAE severity (mild, moderate, severe, life-threatening consequences, Death related to AE)
- Causal relationship to the treatment (yes, no, possible, unknown)
- Expected event (yes, no)
- Action taken
- Outcome (Recovered, Recovering, Ongoing, Fatal, Unknown)

Ongoing adverse events and serious adverse events for each patient should be listed giving both preferred term and the original term used by the investigator. The listing should be by center and by treatment and should include the same variables as the listing of all adverse events (see Listing 8 and Listing 9).

All adverse events and serious adverse events with definite relationship for each patient should be listed giving both preferred term and the original term used by the investigator. The

listing should be by center and by treatment and should include the same variables as the listing of all adverse events (see Listing 10 and Listing 11).

A summary according to ICH E3 12.2.2 will be given for each treatment group and the complete safety data set (see Table 38 and Table 39). Additionally, a summary according to E2F and ENTR/CT 3 Revision 2 using SOC of MedDRA coding will be given. Only relatively common adverse events will be shown, e.g. those in at least 1% of the safety data set (see Table 40 and Table 41).

Listings, containing the same variables as called for above will be provided for deaths. All deaths during the study, including the post-treatment follow-up-period, and deaths that resulted from a process that began during the study, will be listed by patient (see Listing 12).

All serious adverse events of special interest will be listed containing the same variables as called for above (see Listing 13, Listing 14, Listing 15 and Listing 16).

Furthermore, the following patient data will be listed by center and by treatment containing the same variables as called for above:

- Patients with at least one AE (see Listing 17).
- Patients with at least one SAE (see Listing 18).

9.5.2.3 Laboratory tests
Not assessed.

9.5.2.4 Vital signs
Not assessed.

9.5.3 Quality of life
Not assessed.

9.5.4 Health economics
Not assessed.

9.6 Planned subgroup analyses
No subgroup analysis is planned.

9.7 Adjustments for covariates
All analysis will be adjusted for age, sex and study site. If the analyzed endpoint is a difference to baseline the analysis will be adjusted for baseline additionally.

9.7.1 Imputation of covariates
There will be no need for imputation in all covariates defined in the protocol (sex, age, site, see Chapter 9.7) since all covariates are part of the patient's ID (PID). Thus, if sex is missing the respective part of the PID will be used, if age is missing it will be set to respective part of the PID. Site as part of the PID cannot be missing.
Imputation of baseline values in case of endpoints being differences to baseline is described in the respective sections of Chapters 9.4 and 9.5.

9.8 Interim analyses
One planned interim analysis for efficacy and futility will be performed after the day 31 visit of the 322th preterm infant (see Chapter 6). Stopping boundaries for efficacy and futility will be re-estimated using the true sample size of the population to be analyzed in the interim analysis. Re-estimation will be performed using ADDPLAN10. Information rate will be calculated using preterm infants who can be used for analysis of primary endpoint divided by total sample size

of 644. Dysbiosis rate are assumed to be 7.5 and 15 % in verum and placebo group, respectively. Total α will be 0.025 and one sided test for superiority will be chosen. For calculation of α errors for interim analysis, the same setting as during planning of the study will be taken. Futility boundary is binding and will be set to α0 = 0.7 which corresponds to a boundary of -0.524 on the z-scale. Delta is chosen as 0.35.

An adaption of the number of interim analyses or the planned sample size will be performed using the conditional power approach during this interim analysis.

10. Deviations from the protocol

10.1 Definition of analysis populations

For the per protocol population, the number of days the drug has to be administered was lowered to 14 days instead of 28 days as stated in the protocol.

10.2 Timing windows for follow-up visits

Timing windows of follow-up visits is stated more precisely than in the study protocol and set to ± 2 weeks (see Chapter 5.2). After 05.04.2019 time between randomization and follow-up is additionally adjusted by gestational week. After March 2020 the timing window of follow-up visits was enlarged to – 2 weeks up to + 8 weeks.

10.3 Analysis of secondary efficacy endpoints

10.3.1 Growth parameters and metabolism/vital signs

Growth parameters (weight gain, velocity of growth for head circumference and velocity of body length during primary hospital stay, 12-months body weight, body length and head circumference) and metabolism/vital signs at 12- months FU (diastolic and systolic blood pressure) as continuous parameters are not analyzed using a generalized linear mixed model but a linear mixed model.

10.3.2 Gut dysbiosis on day 365

For the analysis of gut dysbiosis on day 365 microbiome data of the PRIMAL preterm infants is compared to microbiome data available from databases of healthy infants aged 12 months. This analysis is prepared by the Bork lab as stated in the project proposal.

10.3.3 Development of immunological parameter during the first year

This endpoint is not subject of this SAP. This parameter will be defined based on the experimental cell marker analysis and is not part of this SAP.

10.3.4 Postnatal exposure to antibiotics during primary hospital stay

Daily dose per kilogram of body weight and treatment with antibiotics per 1000 patient days will both be analyzed descriptively by type of antibiotic and treatment group. No GLMM will be estimated. No adjustment or stratification will be performed.

10.4 Imputation of primary endpoint

Imputation of primary endpoint will not be performed using multiple imputation methods but using the model using the microbiome of the preterm infants done by the EMBL Heidelberg.

10.5 Coding of SAEs

Severity was assessed on CTCTAE severity scale, however coding was not done using CTCAE terms but MedDRA coding.

11. Interpretation of results

Decision making will only be performed on primary endpoint. All other p-values are only descriptive. Analyses of secondary endpoints support analysis of primary endpoint.
An interim for efficacy and futility was planned. Futility binding was chosen to be mandatory. The DSMB is forced to stop the trial in case of $\alpha a > \alpha 0$. Type I error is only protected if fultility boundary is adhered to.

12. Further Analyses

Further analyses on the data of PRIMAL clinical study are not subject to this SAP and p-values are only used in a descriptive manner. Primal clinical study data can be used for statistical methodological research after publication of the result.

13. Software

For analyses, SAS version 9.4 and R Version 3.5.1 or higher and ADDPLAN10 will be used.

14. References

15. Appendices

15.1 Planned tables

15.1.1 Protocol deviations

Table 4 Protocol deviations by centre, treatment group and category

Centre	Intervention	Category	Number of Minor protocol deviations	Number of major protocol deviations
Centre A	Verum	Randomization		
		Inclusion/Exclusion		
	Placebo	Randomization		
		Other		
Centre B	Verum	Randomization		
		Other		
	Placebo	Performance of study procedure		
		Compliance		

Table 5 Crosstable: Intervention and protocol deviations

Intervention	Minor Protocol deviations	Major Protocol deviations
Verum		

Placebo		

15.1.2 Analysis of Intervention Group Comparability

15.1.2.1 Demographics and Baseline Characteristics

Table 6 Birth characteristics.

	Total		Verum		Placebo	
	**n (%)**	**n****	**n (%)**	**n****	**n (%)**	**n****
Multiple birth	X (X.XX%)	X	X (X.XX%)	X	X (X.XX%)	X
Delivery		X		X		X
Spontaneous	X (X.XX%)		X (X.XX%)		X (X.XX%)	
Caesarean section	X (X.XX%)		X (X.XX%)		X (X.XX%)	
Emergency caesarean section	X (X.XX%)		X (X.XX%)		X (X.XX%)	
If caesarean or emergency caesarean section: Uterus without labour	X (X.XX%)	X	X (X.XX%)	X	X (X.XX%)	X
Antenatal steroids	X (X.XX%)	X	X (X.XX%)	X	X (X.XX%)	X
completed cycle (2 doses, 12 h after 2nd dose)	X (X.XX%)	X	X (X.XX%)	X	X (X.XX%)	X
Rupture of membranes before labour*	X (X.XX%)	X	X (X.XX%)	X	X (X.XX%)	X

* Only mothers with given informed consent version 04 and higher, **Number of non-missing values

Table 7 Demographics of preterm infants.

	Total		Verum		Placebo	
	**n (%)<br>Median (IQR)<br>Mean (SD)***	**n****	**n (%)<br>Median (IQR)<br>Mean (SD)***	**n****	**n (%)<br>Median (IQR)<br>Mean (SD)***	**n****
Sex: female	X (X.XX%)	X	X (X.XX%)	X	X (X.XX%)	X
Birth weight (in g)	X.XX (X.XX)	X	X.XX (X.XX)	X	X.XX (X.XX)	X
Body length (in cm)	X.XX (X.XX)	X	X.XX (X.XX)	X	X.XX (X.XX)	X
Temperature (in °C)	X.XX (X.XX)	X	X.XX (X.XX)	X	X.XX (X.XX)	X
Head circumference (in cm)	X.XX (X.XX)	X	X.XX (X.XX)	X	X.XX (X.XX)	X
Gestational age (in GW)	X.XX (X.XX-X.XX)	X	X.XX (X.XX-X.XX)	X	X.XX (X.XX-X.XX)	X

SD: Standard deviation: IQR: Inter quartile range; *For qualitative characteristics n (%); for quantitative characteristics mean (SD) or median (IQR), respectively; **Number of non-missing values

Table 8 Descriptive statistics for quantitative characteristics of demographics of preterm infants.

Group	Statistics	Birth weight (in g)	Body length (in cm)	Temperature (in °C)	Head circumference (in cm)	Gestational Age (in GW)
**Total (N=XXX)**	n*	X	X	X	X	X
	Mean	X.X	X.X	X.X	X.X	XX+X
	Standard deviation	X.X	X.X	X.X	X.X	XX+X
	Minimum	X	X.X	X.X	X.X	XX+X
	1. Quartile	X.X	X.X	X.X	X.X	XX+X
	Median	X.X	X.X	X.X	X.X	XX+X
	3. Quartile	X.X	X.X	X.X	X.X	XX+X
	Maximum	X	X.X	X.X	X.X	XX+X
**Verum (N_1=XXX)**	n*	X	X	X	X	X
	Mean	X.XX	X.X	X.X	X.X	XX+X
	Standard deviation	X:XX	X.X	X.X	X.X	XX+X
	Minimum	X	X.X	X.X	X.X	XX+X
	1. Quartile	X.X	X.X	X.X	X.X	XX+X
	Median	X.X	X.X	X.X	X.X	XX+X
	3. Quartile	X.X	X.X	X.X	X.X	XX+X
	Maximum	X	X.X	X.X	X.X	XX+X
**Placebo (N_2=XXX)**	n*	X	X	X	X	X
	Mean	X.XX	X.X	X.X	X.X	XX+X
	Standard deviation	X:XX	X.X	X.X	X.X	XX+X
	Minimum	X	X.X	X.X	X.X	XX+X
	1. Quartile	X.X	X.X	X.X	X.X	XX+X
	Median	X.X	X.X	X.X	X.X	XX+X
	3. Quartile	X.X	X.X	X.X	X.X	XX+X
	Maximum	X	X.X	X.X	X.X	XX+X

n*: Number of non-missing values

Table 9 Early Bonding/Microbiome.

	**Total**		**Verum**		**Placebo**	
	**n (%) Median (IQR)**	**n****	**n (%)**	**n****	**n (%)**	**n****
Bonding in delivery room	X (X.XX%)	X	X (X.XX%)	X	X (X.XX%)	X
mouth to breast contact, non-nutritive sucking”	X (X.XX%)	X	X (X.XX%)	X	X (X.XX%)	X
First kangorooing	X (X.XX%)	X	X (X.XX%)	X	X (X.XX%)	X
Day of life	X (X - X)	X	X (X - X)	X	X (X - X)	X
Seeding	X (X.XX%)	X	X (X.XX%)	X	X (X.XX%)	X
Mothers hospital stay (> 1 week before delivery)*	X (X.XX%)	X	X (X.XX%)	X	X (X.XX%)	X

* Only in mothers with informed consent version 4 or higher; **Number of non-missing values; IQR: Inter Quartile Range

Table 10 Descriptive statistics for quantitative characteristics of Early Bonding/Microbiome.

Group	Statistics	First kangorooing: Day of life
**Total (N=XXX)**	n*	X
	Mean	X.X
	Standard deviation	X.X
	Minimum	X
	1. Quartile	X
	Median	X
	3. Quartile	X
	Maximum	X
**Verum (N_1=XXX)**	n*	X
	Mean	X.X
	Standard deviation	X.X
	Minimum	X
	1. Quartile	X
	Median	X
	3. Quartile	X
	Maximum	X
**Placebo (N_2=XXX)**	n*	X
	Mean	X.X
	Standard deviation	X.X
	Minimum	X
	1. Quartile	X
	Median	X
	3. Quartile	X
	Maximum	X

n*: Number of non-missing values

15.1.2.2 Prior or concomitant diseases and risk factors

Table 11 Cause of preterm delivery.

	**Total**		**Verum**		**Placebo**	
	**n (%)**	**n****	**n (%)**	**n****	**n (%)**	**n****
Preterm labour	X (X.XX%)	X	X (X.XX%)	X	X (X.XX%)	X
Pathological cardiotocography	X (X.XX%)	X	X (X.XX%)	X	X (X.XX%)	X
Amniotic infection syndrome (suspected or proven)	X (X.XX%)	X	X (X.XX%)	X	X (X.XX%)	X
Intrauterine growth restrictions, pathological doppler	X (X.XX%)	X	X (X.XX%)	X	X (X.XX%)	X
Pre-eclampsia	X (X.XX%)	X	X (X.XX%)	X	X (X.XX%)	X
Placenta abruption	X (X.XX%)	X	X (X.XX%)	X	X (X.XX%)	X

	Total		Verum		Placebo	
	**n (%)**	**n****	**n (%)**	**n****	**n (%)**	**n****
HELLP-syndrome	X (X.XX%)	X	X (X.XX%)	X	X (X.XX%)	X
Rupture of membranes > 5 days of delivery, anhydramnios	X (X.XX%)	X	X (X.XX%)	X	X (X.XX%)	X
Prolaps of mebranes	X (X.XX%)	X	X (X.XX%)	X	X (X.XX%)	X
Rupture of mebranes without anhydramnios	X (X.XX%)	X	X (X.XX%)	X	X (X.XX%)	X
Other	X (X.XX%)	X	X (X.XX%)	X	X (X.XX%)	X

**Number of non-missing values

*Table 12 Mothers data**

	Total		Verum		Placebo	
	**n (%)<br>Mean (SD)<br>Median (IQR)**	**n****	**n (%)<br>Mean (SD)<br>Median (IQR)**	**n****	**n (%)<br>Mean (SD)<br>Median (IQR)**	**n****
Age (in years)	X.XX (X.XX)	X	X.XX (X.XX)	X	X.XX (X.XX)	X
Body weight at diagnosis of pregnancy (in kg)	X.XX (X.XX)	X	X.XX (X.XX)	X	X.XX (X.XX)	X
Body length (in cm)	X.XX (X.XX)	X	X.XX (X.XX)	X	X.XX (X.XX)	X
Blood group		X		X		X
A	X (X.XX%)	X	X (X.XX%)	X	X (X.XX%)	X
B	X (X.XX%)	X	X (X.XX%)	X	X (X.XX%)	X
AB	X (X.XX%)	X	X (X.XX%)	X	X (X.XX%)	X
O	X (X.XX%)	X	X (X.XX%)	X	X (X.XX%)	X
Former pregnancies/deliveries	X (X.XX%)	X	X (X.XX%)	X	X (X.XX%)	X
Number of pregnancies (without actual delivery)	X.X (X.X – X.X)	X	X.X (X.X – X.X)	X	X.X (X.X – X.X)	X
Number of deliveries (without actual delivery)	X.X (X.X – X.X)	X	X.X (X.X – X.X)	X	X.X (X.X – X.X)	X
Preterm deliveries before 37+0 GW (without actual delivery)	X (X.XX%)	X	X (X.XX%)	X	X (X.XX%)	X

* Only mothers with written informed consent version 04 and higher; **Number of non-missing values; SD: Standard Deviation; IQR: Inter-Quartile Range

Table 13 Descriptive statistics for quantitative characteristics of Mothers data.

Group	Statistics	Age (in years)	Body weight at diagnosis of pregnancy (in kg)	Body length (in cm)	Number of pregnancies (without actual delivery)	Number of deliveries(without actual delivery)
**Total (N=XXX)**	n*	X	X	X	X	X
	Mean	X.XX	X.XX	X.XX	X.XX	X.XX
	Standard deviation	X.XX	X.XX	X.XX	X.XX	X.XX
	Minimum	X.XX	X.XX	X.XX	X.XX	X.XX
	1. Quartile	X.XX	X.XX	X.XX	X.XX	X.XX
	Median	X.XX	X.XX	X.XX	X.XX	X.XX
	3. Quartile	X.XX	X.XX	X.XX	X.XX	X.XX

Group	Statistics	Age (in years)	Body weight at diagnosis of pregnancy (in kg)	Body length (in cm)	Number of pregnancies (without actual delivery)	Number of deliveries(without actual delivery)
	Maximum	X.XX	X.XX	X.XX	X.XX	X.XX
**Verum (N_1=XXX)**	n*	X	X	X	X	X
	Mean	X.XX	X.XX	X.XX	X.XX	X.XX
	Standard deviation	X.XX	X.XX	X.XX	X.XX	X.XX
	Minimum	X.XX	X.XX	X.XX	X.XX	X.XX
	1. Quartile	X.XX	X.XX	X.XX	X.XX	X.XX
	Median	X.XX	X.XX	X.XX	X.XX	X.XX
	3. Quartile	X.XX	X.XX	X.XX	X.XX	X.XX
	Maximum	X.XX	X.XX	X.XX	X.XX	X.XX
**Placebo (N_2=XXX)**	n*	X	X	X	X	X
	Mean	X.XX	X.XX	X.XX	X.XX	X.XX
	Standard deviation	X.XX	X.XX	X.XX	X.XX	X.XX
	Minimum	X.XX	X.XX	X.XX	X.XX	X.XX
	1. Quartile	X.XX	X.XX	X.XX	X.XX	X.XX
	Median	X.XX	X.XX	X.XX	X.XX	X.XX
	3. Quartile	X.XX	X.XX	X.XX	X.XX	X.XX
	Maximum	X.XX	X.XX	X.XX	X.XX	X.XX

n*: Number of non-missing values

*Table 14 Antenatal antibiotics**

	**Total**		**Verum**		**Placebo**	
	**n (%)**	**n****	**n (%)**	**n****	**n (%)**	**n****
Antenatal antibiotics	X (X.XX%)	X	X (X.XX%)	X	X (X.XX%)	X
Ampicillin/andere Penicilline	X (X.XX%)	X	X (X.XX%)	X	X (X.XX%)	X
Cephalosporine	X (X.XX%)	X	X (X.XX%)	X	X (X.XX%)	X
Imiprenem/Meropenem	X (X.XX%)	X	X (X.XX%)	X	X (X.XX%)	X
Metronidazol	X (X.XX%)	X	X (X.XX%)	X	X (X.XX%)	X
Makrolide (Erythromycin, Azithromycin)	X (X.XX%)	X	X (X.XX%)	X	X (X.XX%)	X
Other	X (X.XX%)	X	X (X.XX%)	X	X (X.XX%)	X

* Only mothers with written informed consent version 04 and higher, **Number of non-missing values

15.1.3 Exposition to Treatment/Compliance

Table 15 Descriptive statistics for exposition to treatment/compliance (As Treated/Safety analysis population).

Group	Statistics	Duration of start and stop of medication (in days)	Days without medication between start and stop of medication (in days)	Drug accountability (Number of pills left)
	n*	X	X	X
	Mean	X.X	X.X	X.X
	Standard deviation	X.X	X.X	X.X
**Total (N=XXX)**	Minimum	X	X	X
	1. Quartile	X	X	X
	Median	X	X	X
	3. Quartile	X	X	X
	Maximum	X	X	X
	n*	X	X	X
	Mean	X.X	X.X	X.X
	Standard deviation	X.X	X.X	X.X
**Verum (N_1=XXX)**	Minimum	X	X	X
	1. Quartile	X	X	X
	Median	X	X	X
	3. Quartile	X	X	X
	Maximum	X	X	X
	n*	X	X	X
	Mean	X.X	X.X	X.X
	Standard deviation	X.X	X.X	X.X
**Placebo (N_2=XXX)**	Minimum	X	X	X
	1. Quartile	X	X	X
	Median	X	X	X
	3. Quartile	X	X	X
	Maximum	X	X	X

n*: Number of non-missing values, ** If primary hospital was longer than 31 days (time of primary endpoint) truncated to 31 days

15.1.4 Primary Analyses

Table 16 Crosstable of treatment and gut dysbiosis on day 31

		Gut dysbiosis on day 31		
		no	yes	Total*

Treatment	Verum	XXX (X.XX%)	XXX (X.XX%)	N_1
	Placebo	XXX (X.XX%)	XXX (X.XX%)	N_2
	Total	XXX (X.XX%)	XXX (X.XX%)	N

* N_1: Number of preterm infants treated with verum, N_2: Number of preterm infants treted with placebo

Table 17 Results from generalized linear mixed model for gut dysbiosis on day 31

			95 % CI of		$(1-\alpha_a) \cdot 100$ % CI of		
Effect	Estimate	SE	Estimate	OR	Estimate	OR	p-value
Verum vs. Placebo							

CI: confidence interval, OR: odds ratio, α_a: adjusted alpha for analysis a (either interim or main analysis, see Chapter 9.8)

15.1.5 Secondary Analyses

15.1.5.1 Efficacy

15.1.5.1.1 Compound endpoint gut dysbiosis on day 31

Table 18 Crosstable of treatment and compound endpoint gut dysbiosis on day 31

		Gut dysbiosis on day 31		
		no	yes	Total*
Treatment	Verum	XXX (X.XX%)	XXX (X.XX%)	N_1
	Placebo	XXX (X.XX%)	XXX (X.XX%)	N_2
	Total	XXX (X.XX%)	XXX (X.XX%)	N

* N_1: Number of preterm infants treated with verum, N_2: Number of preterm infants treted with placebo

Table 19 Results from generalized linear mixed model for compound endpoint gut dysbiosis on day 31

			95 % CI of		
Effect	Estimate	SE	Estimate	OR	p-value

CI: confidence interval, OR: odds ratio

15.1.5.1.2 Clinical sepsis during primary hospital stay

Table 20 Crosstable of treatment and clinical sepsis during primary hospital stay

		Clinical sepsis during primary hospital stay		
		no	yes	Total*
Treatment	Verum	XXX (X.XX%)	XXX (X.XX%)	N_1
	Placebo	XXX (X.XX%)	XXX (X.XX%)	N_2
	Total	XXX (X.XX%)	XXX (X.XX%)	N

* N_1: Number of preterm infants treated with verum, N_2: Number of preterm infants treted with placebo

Table 21 Results from generalized linear mixed model for clinical sepsis during primary hospital stay

			95 % CI of		
Effect	Estimate	SE	Estimate	OR	p-value

CI: confidence interval, OR: odds ratio

15.1.5.1.3 Blood-culture proven sepsis during primary hospital stay

Table 22 Crosstable of treatment and blood-culture proven sepsis during primary hospital stay

		Blood-culture proven sepsis during primary hospital stay		
		no	yes	Total*
Treatment	Verum	XXX (X.XX%)	XXX (X.XX%)	N_1
	Placebo	XXX (X.XX%)	XXX (X.XX%)	N_2
	Total	XXX (X.XX%)	XXX (X.XX%)	N

* N_1: Number of preterm infants treated with verum, N_2: Number of preterm infants treted with placebo

Table 23 Results from generalized linear mixed model for blood-culture proven sepsis during primary hospital stay

			95 % CI of		
Effect	Estimate	SE	Estimate	OR	p-value

CI: confidence interval, OR: odds ratio

15.1.5.1.4 Postnatal exposure to antibiotics during primary hospital stay

Daily dose per kilogram of body weight and Treatment with antibiotics per 1000 patient days

Table 24 Daily dose per kilogram body weight treatment with antibiotics per 1000 patient days

Group	Type of Antibiotic	Number of treated preterm infants	Patient days	Daily dose per kilogram body weight Median (IQR)*	Treatment with antibiotics per 100 patient days
**Total (N=XXX)**	Antibiotic 1	X	X	X (X – X)	X.XX
	Antibiotic 2	X	X	X (X – X)	X.XX
	Antibiotic n	X	X	X (X – X)	X.XX
**Verum (N_1=XXX)**	Antibiotic 1	X	X	X (X – X)	X.XX
	Antibiotic 2	X	X	X (X – X)	X.XX
	Antibiotic n	X	X	X (X – X)	X.XX
**Placebo (N_2=XXX)**	Antibiotic 1	X	X	X (X – X)	X.XX
	Antibiotic 2	X	X	X (X – X)	X.XX
	Antibiotic n	X	X	X (X – X)	X.XX

* Only in treated preterm infants

Number of antibiotic cycles

Table 25 Results from generalized linear mixed model for number of antibiotic cycles during primary hospital stay

			95 % CI of	
Effect	Estimate	SE	Estimate	p-value

CI: confidence interval, OR: odds ratio

15.1.5.1.5 Infectious episodes in the first year of life

Table 26 Crosstable of treatment and [infection] during first year of life

		Blood-culture proven sepsis during primary hospital stay		
		no	yes	Total*
Treatment	Verum	XXX (X.XX%)	XXX (X.XX%)	N_1
	Placebo	XXX (X.XX%)	XXX (X.XX%)	N_2
	Total	XXX (X.XX%)	XXX (X.XX%)	N

* N_1: Number of preterm infants treated with verum, N_2: Number of preterm infants treted with placebo

Table 27 Results from generalized linear mixed model for [infection] during first year of life

			95 % CI of		
Effect	Estimate	SE	Estimate	OR	p-value

CI: confidence interval, OR: odds ratio

15.1.5.1.6 Wheezing episodes during the first year of life

Table 28 Crosstable of treatment and kind of wheezing episodes during first year of life

		Blood-culture proven sepsis during primary hospital stay		
		no	yes	Total*
Treatment	Verum	XXX (X.XX%)	XXX (X.XX%)	N_1
	Placebo	XXX (X.XX%)	XXX (X.XX%)	N_2
	Total	XXX (X.XX%)	XXX (X.XX%)	N

* N_1: Number of preterm infants treated with verum, N_2: Number of preterm infants treted with placebo

Table 29 Results from generalized linear mixed model for wheezing episodes during first year of life

			95 % CI of		
Effect	Estimate	SE	Estimate	OR	p-value

CI: confidence interval, OR: odds ratio

15.1.5.1.7 Atopic dermatitis during the first year of life

Table 30 Crosstable of treatment and kind of atopic dermatitis during first year of life

		Blood-culture proven sepsis during primary hospital stay		
		no	yes	Total*
Treatment	Verum	XXX (X.XX%)	XXX (X.XX%)	N_1
	Placebo	XXX (X.XX%)	XXX (X.XX%)	N_2
	Total	XXX (X.XX%)	XXX (X.XX%)	N

* N_1: Number of preterm infants treated with verum, N_2: Number of preterm infants treted with placebo

Table 31 Results from generalized linear mixed model for atopic dermatitis during first year of life

			95 % CI of		
Effect	Estimate	SE	Estimate	OR	p-value

CI: confidence interval, OR: odds ratio

15.1.5.1.8 Growth parameters

Table 32 Results from linear mixed model for [growth parameter]

			95 % CI of	
Effect	Estimate	SE	Estimate	p-value

CI: confidence interval, OR: odds ratio

15.1.5.1.9 Nutritional aspects

Table 33 Crosstable of treatment and [nutritional aspect]

		Blood-culture proven sepsis during primary hospital stay		
		no	yes	Total*
Treatment	Verum	XXX (X.XX%)	XXX (X.XX%)	N_1
	Placebo	XXX (X.XX%)	XXX (X.XX%)	N_2
	Total	XXX (X.XX%)	XXX (X.XX%)	N

* N_1: Number of preterm infants treated with verum, N_2: Number of preterm infants treted with placebo

Table 34 Results from generalized linear mixed model for [nutritional aspect]

			95 % CI of		
Effect	Estimate	SE	Estimate	OR	p-value

CI: confidence interval, OR: odds ratio

15.1.5.1.10 Metabolism/Vital signs

Table 35 Results from linear mixed model for [vital sign]

			95 % CI of	
Effect	Estimate	SE	Estimate	p-value

CI: confidence interval, OR: odds ratio

15.1.5.2 Safety/Tolerability

15.1.5.2.1 Serious and non-serious events

Table 36 Characteristics of Adverse Events and Serious Adverse Events as documented in the AE CRF

		Complete SA data set (N=XXX)	Verum (N_1=XXX)	Placebo (N_2=XXX)
Serious and non-serious AEs (N=XXX)	Total Number	XX	XX	XX

		Complete SA data set (N=XXX)	Verum (N_1=XXX)	Placebo (N_2=XXX)
	Median time in d (IQR)	XX (XX – XX)	XX (XX – XX)	XX (XX – XX)
	Severity			
	Mild	X	X	X
	Moderate	X	X	X
	Severe	X	X	X
	Life threatening consequences	X	X	X
	Death related to AE	X	X	X
	Causal relationship to the treatment (verum or placebo)			
	Related	X	X	X
	Not related	X	X	X
	Status/outcome of event			
	Recovered	X	X	X
	Recovered with sequel	X	X	X
	Not yet recovered, further treatment not necessary	X	X	X
	Not yet recovered, further treatment necessary	X	X	X
	Fatal	X	X	X
	Unknown	X	X	X
Non-serious AEs (N=XXX)	Number	XX	XX	XX
	Median time in d (IQR)	XX (XX – XX)	XX (XX – XX)	XX (XX – XX)
	Severity			
	Mild	X	X	X
	Moderate	X	X	X
	Severe	X	X	X
	Life threatening consequences	X	X	X
	Death related to AE	X	X	X
	Causal relationship to the treatment (verum or placebo)			
	Related	X	X	X
	Not related	X	X	X
	Status/outcome of event			
	Recovered	X	X	X
	Recovered with sequel	X	X	X
	Anhaltend, keine Nachbehandlung erforderlich	X	X	X
	Anhaltend, Nachbehandlung erforderlich	X	X	X
	Fatal	X	X	X
	Unknown	X	X	X
Serious AEs (N=XXX)	Number	XX	XX	XX
	Median time in d (IQR)	XX (XX – XX)	XX (XX – XX)	XX (XX – XX)
	Severity			
	Mild	X	X	X

		Complete SA data set (N=XXX)	Verum (N_1=XXX)	Placebo (N_2=XXX)
	Moderate<br>Severe<br>Life threatening consequences<br>Death related to AE	X<br>X<br>X<br>X	X<br>X<br>X<br>X	X<br>X<br>X<br>X
	Causal relationship to the treatment (verum or placebo)<br>Related<br>Not related	 X<br>X	 X<br>X	 X<br>X
	Status/outcome of event<br>Recovered<br>Recovered with sequel<br>Anhaltend, keine Nachbehandlung erforderlich<br>Anhaltend, Nachbehandlung erforderlich<br>Fatal<br>Unknown	 X<br>X<br>X<br>X<br>X<br>X	 X<br>X<br>X<br>X<br>X<br>X	 X<br>X<br>X<br>X<br>X<br>X

IQR: Inter-quartile range; d: days

Table 37 Characteristics of Adverse Events and Serious Adverse Events as documented in the SAE CRF

	Complete SA data set (N=XXX)	Verum (N_1=XXX)	Placebo (N_2=XXX)
Number<br>Median time in d (IQR)<br>Ongoing<br>Expected event<br>Unexpected event	XX<br>XX (XX – XX)<br>XX (XX.XX %)<br>XX (XX.XX %)<br>XX (XX.XX %)	XX<br>XX (XX – XX)<br>XX (XX.XX %)<br>XX (XX.XX %)<br>XX (XX.XX %)	XX<br>XX (XX – XX)<br>XX (XX.XX %)<br>XX (XX.XX %)<br>XX (XX.XX %)
Serious criterium<br>Reaults in death<br>Is life-threatening<br>Results in persistent or significant disability/incapacity<br>Requires inpatient hospitalization or causes prolongation of existing hospitalization<br>Requires intervention to prevent permanent impairment or damage<br>May have caused a congenital anormaly/birth defect	 X<br>X<br>X<br>X<br>X<br>X	 X<br>X<br>X<br>X<br>X<br>X	 X<br>X<br>X<br>X<br>X<br>X
SAE of special interest<br>Blood-culture proven sepsis<br>Clinical sepsis<br>Necrotizing enterocolitis (NEC) ≥ Bell's stage 2<br>Severe gastrointestinal complication	X<br>X<br>X<br>X<br>X	X<br>X<br>X<br>X<br>X	X<br>X<br>X<br>X<br>X
CTCAE severity scale<br>Mild<br>Moderate	X<br>X<br>X	X<br>X<br>X	X<br>X<br>X

	Complete SA data set (N=XXX)	Verum (N_1=XXX)	Placebo (N_2=XXX)
Severe<br>Life threatening consequences<br>Death related to AE	X<br>X<br>X	X<br>X<br>X	X<br>X<br>X
Causal relationship to the treatment (verum or placebo)<br>Related<br>Not related	X<br>X<br>X	X<br>X<br>X	X<br>X<br>X
Status/Outcome of event<br>Recovered<br>Recovering<br>Ongoing<br>Fatal<br>Unknown			

IQR: Inter-quartile range; d: days

*Table 38 Adverse Events: Preferred Term Number Observed and Rate, with Patient Identifications for complete safety data set/ Verum Group/ Placebo Group (N=XXX)**

Severity	Mild			Moderate			Severe			Life-threatening consequences			Death related to AE			Total			Total
Relationship	NR	R	T	NR	R	T	NR	R	T	NR	R	T	NR	R	T	NR	R	T	
Body System A<br>Event 1**<br><br><br><br>Event 2**	X (X.XX)<br>ID1<br>ID2<br>ID3	X (X.XX)<br>ID1<br>ID2<br>ID3	X (X.XX)	X (X.XX)<br>ID1<br>ID2<br>ID3	X (X.XX)<br>ID1<br>ID2<br>ID3	X (X.XX)	X (X.XX)<br>ID1<br>ID2<br>ID3	X (X.XX)<br>ID1<br>ID2<br>ID3	X (X.XX)	X (X.XX)<br>ID1<br>ID2<br>ID3	X (X.XX)<br>ID1<br>ID2<br>ID3	X (X.XX)	X (X.XX)<br>ID1<br>ID2<br>ID3	X (X.XX)<br>ID1<br>ID2<br>ID3	X (X.XX)	X (X.XX)	X (X.XX)	X (X.XX)	X (X.XX)
Body System B<br>Event 1**<br>Event 2**																			
Total	X (X.XX)	X (X.XX)	X (X.XX)	X (X.XX)	X (X.XX)	X (X.XX)	X (X.XX)	X (X.XX)	X (X.XX)	X (X.XX)	X (X.XX)	X (X.XX)	X (X.XX)	X (X.XX)	X (X.XX)	X (X.XX)	X (X.XX)	X (X.XX)	X (X.XX)

*one table for each group **Preferred Term, NR: not related (no relationship or unlikey relationship), R: related (possible, probable, definite unknown relationship), T: Total

*Table 39 Serious Adverse Events: Preferred Term Number Observed and Rate, with Patient Identifications for complete safety data set/ Verum Group/ Placebo Group (N=XXX)**

Severity	Mild			Moderate			Severe			Life-threatening consequences			Death related to AE			Total			Total
Relationship	NR	R	T	NR	R	T	NR	R	T	NR	R	T	NR	R	T	NR	R	T	
Body System A<br>Event 1**<br><br><br><br>Event 2**	X (X.XX)<br>ID1<br>ID2<br>ID3	X (X.XX)<br>ID1<br>ID2<br>ID3	X (X.XX)	X (X.XX)<br>ID1<br>ID2<br>ID3	X (X.XX)<br>ID1<br>ID2<br>ID3	X (X.XX)	X (X.XX)<br>ID1<br>ID2<br>ID3	X (X.XX)<br>ID1<br>ID2<br>ID3	X (X.XX)	X (X.XX)<br>ID1<br>ID2<br>ID3	X (X.XX)<br>ID1<br>ID2<br>ID3	X (X.XX)	X (X.XX)<br>ID1<br>ID2<br>ID3	X (X.XX)<br>ID1<br>ID2<br>ID3	X (X.XX)	X (X.XX)	X (X.XX)	X (X.XX)	X (X.XX)
Body System B<br>Event 1**<br>Event 2**																			
Total	X (X.XX)	X (X.XX)	X (X.XX)	X (X.XX)	X (X.XX)	X (X.XX)	X (X.XX)	X (X.XX)	X (X.XX)	X (X.XX)	X (X.XX)	X (X.XX)	X (X.XX)	X (X.XX)	X (X.XX)	X (X.XX)	X (X.XX)	X (X.XX)	X (X.XX)

*one table for each group **Preferred Term, NR: not related (no relationship or unlikey relationship), R: related (possible, probable, definite unknown relationship), T: Total

Table 40 Summary and Comparison between Verum (N_1=XXX) and Placebo (N_2=XXX) Most Common Adverse events: Preferred Terms

Severity		Mild			Moderate			Severe			Life-threatening consequences			Death related to AE			Total			Total***
Relationship		NR	R	T	NR	R	T	NR	R	T	NR	R	T	NR	R	T	NR	R	T	
Body System A																				
Event 1**	Verum	X (X.XX)	X (X.XX)	X (X.XX)	X (X.XX)	X (X.XX)	X (X.XX)	X (X.XX)	X (X.XX)	X (X.XX)	X (X.XX)	X (X.XX)	X (X.XX)	X (X.XX)	X (X.XX)	X (X.XX)	X (X.XX)	X (X.XX)	X (X.XX)	X (X.XX)
	Placebo	X (X.XX)	X (X.XX)	X (X.XX)	X (X.XX)	X (X.XX)	X (X.XX)	X (X.XX)	X (X.XX)	X (X.XX)	X (X.XX)	X (X.XX)	X (X.XX)	X (X.XX)	X (X.XX)	X (X.XX)	X (X.XX)	X (X.XX)	X (X.XX)	X (X.XX)
Event 2**	Verum	X (X.XX)	X (X.XX)	X (X.XX)	X (X.XX)	X (X.XX)	X (X.XX)	X (X.XX)	X (X.XX)	X (X.XX)	X (X.XX)	X (X.XX)	X (X.XX)	X (X.XX)	X (X.XX)	X (X.XX)	X (X.XX)	X (X.XX)	X (X.XX)	X (X.XX)
	Placebo	X (X.XX)	X (X.XX)	X (X.XX)	X (X.XX)	X (X.XX)	X (X.XX)	X (X.XX)	X (X.XX)	X (X.XX)	X (X.XX)	X (X.XX)	X (X.XX)	X (X.XX)	X (X.XX)	X (X.XX)	X (X.XX)	X (X.XX)	X (X.XX)	X (X.XX)
Body System B<br>Event 1**<br>Event 2**																				
Total	Verum	X (X.XX)	X (X.XX)	X (X.XX)	X (X.XX)	X (X.XX)	X (X.XX)	X (X.XX)	X (X.XX)	X (X.XX)	X (X.XX)	X (X.XX)	X (X.XX)	X (X.XX)	X (X.XX)	X (X.XX)	X (X.XX)	X (X.XX)	X (X.XX)	X (X.XX)
	Placebo	X (X.XX)	X (X.XX)	X (X.XX)	X (X.XX)	X (X.XX)	X (X.XX)	X (X.XX)	X (X.XX)	X (X.XX)	X (X.XX)	X (X.XX)	X (X.XX)	X (X.XX)	X (X.XX)	X (X.XX)	X (X.XX)	X (X.XX)	X (X.XX)	X (X.XX)

*one table for each group **Preferred Term ***all events with at least 1% in the safety data set, NR: not related (no relationship or unlikey relationship), R: related (possible, probable, definite unknown relationship), T: Total

Table 41 Summary and Comparison between Verum (N_1=XXX) and Placebo (N_2=XXX) Most Common Adverse events: Preferred Terms

Severity		Mild			Moderate			Severe			Life-threatening consequences			Death related to AE			Total			Total***
Relationship		NR	R	T	NR	R	T	NR	R	T	NR	R	T	NR	R	T	NR	R	T	
Body System A																				
Event 1**	Verum	X (X.XX)	X (X.XX)	X (X.XX)	X (X.XX)	X (X.XX)	X (X.XX)	X (X.XX)	X (X.XX)	X (X.XX)	X (X.XX)	X (X.XX)	X (X.XX)	X (X.XX)	X (X.XX)	X (X.XX)	X (X.XX)	X (X.XX)	X (X.XX)	X (X.XX)
	Placebo	X (X.XX)	X (X.XX)	X (X.XX)	X (X.XX)	X (X.XX)	X (X.XX)	X (X.XX)	X (X.XX)	X (X.XX)	X (X.XX)	X (X.XX)	X (X.XX)	X (X.XX)	X (X.XX)	X (X.XX)	X (X.XX)	X (X.XX)	X (X.XX)	X (X.XX)
Event 2**	Verum	X (X.XX)	X (X.XX)	X (X.XX)	X (X.XX)	X (X.XX)	X (X.XX)	X (X.XX)	X (X.XX)	X (X.XX)	X (X.XX)	X (X.XX)	X (X.XX)	X (X.XX)	X (X.XX)	X (X.XX)	X (X.XX)	X (X.XX)	X (X.XX)	X (X.XX)
	Placebo	X (X.XX)	X (X.XX)	X (X.XX)	X (X.XX)	X (X.XX)	X (X.XX)	X (X.XX)	X (X.XX)	X (X.XX)	X (X.XX)	X (X.XX)	X (X.XX)	X (X.XX)	X (X.XX)	X (X.XX)	X (X.XX)	X (X.XX)	X (X.XX)	X (X.XX)
Body System B<br>Event 1**<br>Event 2**																				
Total	Verum	X (X.XX)	X (X.XX)	X (X.XX)	X (X.XX)	X (X.XX)	X (X.XX)	X (X.XX)	X (X.XX)	X (X.XX)	X (X.XX)	X (X.XX)	X (X.XX)	X (X.XX)	X (X.XX)	X (X.XX)	X (X.XX)	X (X.XX)	X (X.XX)	X (X.XX)
	Placebo	X (X.XX)	X (X.XX)	X (X.XX)	X (X.XX)	X (X.XX)	X (X.XX)	X (X.XX)	X (X.XX)	X (X.XX)	X (X.XX)	X (X.XX)	X (X.XX)	X (X.XX)	X (X.XX)	X (X.XX)	X (X.XX)	X (X.XX)	X (X.XX)	X (X.XX)

*one table for each group **Preferred Term ***all events with at least 1% in the safety data set, NR: not related (no relationship or unlikey relationship), R: related (possible, probable, definite unknown relationship), T: Total

15.2 Planned listings

15.2.1 Incomplete Treatments, Drop-Outs, Incomplete Observation, and Protocol Deviations

Listing 1 Protocol deviations

Centre	Randomization Result	Actual Treatment	PID	Category	Visit	Date of Monitoring Visit	Date of PD	Description	Procedure	Minor/Major Protocol deviation
Centre A	Verum	Verum								
		Placebo								
		Verum								
	Placebo	Placebo								
		Placebo								
		Verum								
Centre B	Verum	Verum								
		Verum								
		Verum								
	Placebo	Verum								
		Placebo								
		Placebo								

Listing 2 Protocol deviations – Category Y

Centre	Randomization Result	Actual Treatment		PID*	Visit	Date of Monitoring Visit	Date of PD	Description	Procedure	Minor/Major Protocol deviation
Centre A	Verum	Verum								
		Placebo								
		Verum								
	Placebo	Placebo								
		Placebo								
		Verum								
Centre B	Verum	Verum								
		Verum								
		Verum								
	Placebo	Verum								
		Placebo								
		Placebo								

*Either pre-study ID or pheno-number

Listing 3 Listing of patients who discontinued study after randomization

Centre	Randomization result	Actual treatment	PID	Last visit	Reason for discontinuation
Centre A	Verum	Verum			
		Placebo			
		Verum			

Centre	Randomization result	Actual treatment	PID	Last visit	Reason for discontinuation
	Placebo	Placebo			
		Placebo			
		Verum			
Centre B	Verum	Verum			
		Verum			
		Verum			
	Placebo	Verum			
		Placebo			
		Placebo			

15.2.2 Subject disposition

See Listing 3

15.3 Exposition to Treatment/Compliance

Listing 4 Exposition to Treatment and Compliance

**Center**	**Randomization Result**	**Actual Treatment**	**PID**	**Duration of start and stop of medication (in days)**	**Days without medication between start and stop of medication (in days)**	**Drug accountability (Number of pills left)**
Center A	Verum	Verum				
		Verum				
	Placebo	Verum				
		Placebo				
Center B	Verum	Placebo				
		Verum				
	Placebo	Placebo				
		Placebo				

* If primary hospital was longer than 31 days (time of primary endpoint) truncated to 31 days

Listing 5 Protocol deviations – Exposition to treatment/Compliance/Drug Accountability

Centre	Randomization Result	Actual Treatment		PID*	Category	Visit	Date of Monitoring Visit	Date of PD	Description	Procedure	Minor/Major Protocol deviation
Centre A	Verum	Verum									
		Placebo									
		Verum									
	Placebo	Placebo									
		Placebo									
		Verum									
Centre B	Verum	Verum									
		Verum									
		Verum									
	Placebo	Verum									
		Placebo									
		Placebo									

15.3.1 Secondary Analyses

15.3.1.1 Serious and non-serious adverse events

Listing 6 Adverse Events

Centre	Treatment	Patient	Age in days	Sex	Weight*	Height	Description						Date of onset or date of clinic visit	Duration of treatment until date of onset	Duration of AE (in days)	CTCAE Severity	Causal relationship	Action taken	Outcome	Seriousness
							SOC	HLGT	HLT	PT	LLT	Reported Term								
Centre1	Verum	Patient 1																		
		Patient 2																		
	Placebo	Patient 3																		
. . .																				
Centre n	Verum	Patient 4																		
		. . .																		
		Patient 5																		
	Placebo	Patient 6																		

		. . .																		
		Patient 7																		

*on hospital admission

Listing 7 Serious Adverse Events

Centre	Treatment	Patient	Age in days	Sex	Weight*	Height	Description							Date of onset or date of clinic visit	Duration of treatment until date of onset	Duration of AE (in days)	CTCAE Severity	Causal relationship	Expected SAE	Action taken	Outcome
							SOC	HLGT	HLT	PT	LLT	Reported Term	AE of special interest								
Centre1	Verum	Patient 1																			
		Patient 2																			
	Placebo	Patient 3																			
. . .																					
Centre n	Verum	Patient 4																			
		. . .																			
		Patient 5																			
	Placebo	Patient 6																			
		. . .																			
		Patient 7																			

*on hospital admission

Listing 8 Ongoing Adverse Events

Centre	Treatment	Patient	Age in days	Sex	Weight*	Height	Description						Date of onset or date of clinic visit	Duration of treatment until date of onset	Duration of AE (in days)	CTCAE Severity	Causal relationship	Action taken	Outcome	Seriousness
							SOC	HLGT	HLT	PT	LLT	Reported Term								
Centre1	Verum	Patient 1																		
		Patient 2																		
	Placebo	Patient 3																		
. . .																				
Centre n	Verum	Patient 4																		
		. . .																		
		Patient 5																		
	Placebo	Patient 6																		
		. . .																		
		Patient 7																		

Listing 9 Ongoing Serious Adverse Events

Centre	Treatment	Patient	Age in days	Sex	Weight*	Height	Description							Date of onset or date of clinic visit	Duration of treatment until date of onset	Duration of AE (in days)	CTCAE Severity	Causal relationship	Expected SAE	Action taken	Outcome
							SOC	HLGT	HLT	PT	LLT	Reported Term	AE of special interest								
Centre1	Verum	Patient 1																			
		Patient 2																			
	Placebo	Patient 3																			
.																					

Centre	Treatment	Patient	Age in days	Sex	Weight*	Height	Description							Date of onset or date of clinic visit	Duration of treatment until date of onset	Duration of AE (in days)	CTCAE Severity	Causal relationship	Expected SAE	Action taken	Outcome
							SOC	HLGT	HLT	PT	LLT	Reported Term	AE of special interest								
. .																					
Centre n	Verum	Patient 4																			
		. . .																			
		Patient 5																			
	Placebo	Patient 6																			
		. . .																			
		Patient 7																			

Listing 10 Adverse Events with definite relationship

Centre	Treatment	Patient	Age in days	Sex	Weight*	Height	Description						Date of onset or date of clinic visit	Duration of treatment until date of onset	Duration of AE (in days)	CTCAE Severity	Action taken	Outcome	Seriousness
							SOC	HLGT	HLT	PT	LLT	Reported Term							
Centre1	Verum	Patient 1																	
		Patient 2																	
	Placebo	Patient 3																	
. . .																			
Centre n	Verum	Patient 4																	
		.																	

		. .																	
		Patient 5																	
	Placebo	Patient 6																	
		. . .																	
		Patient 7																	

Listing 11 Serious Adverse Events with definite relationship

Centre	Treatment	Patient	Age in days	Sex	Weight*	Height	Description							Date of onset or date of clinic visit	Duration of treatment until date of onset	Duration of AE (in days)	CTCAE Severity	Expected SAE	Action taken	Outcome
							SOC	HLGT	HLT	PT	LLT	Reported Term	AE of special interest							
Centre1	Verum	Patient 1																		
		Patient 2																		
	Placebo	Patient 3																		
. . .																				
Centre n	Verum	Patient 4																		
		. . .																		
		Patient 5																		
	Placebo	Patient 6																		
		. . .																		
		Patient 7																		

Listing 12 Deaths

Centre	Treatment	Patient	Age in days	Sex	Weight*	Height	Description							Date of onset or date of clinic visit	Duration of treatment until date of onset	Duration of AE (in days)	CTCAE Severity	Causal relationship	Expected SAE	Action taken	Date of Death
							SOC	HLGT	HLT	PT	LLT	Reported Term	AE of special interest								
Centre1	Verum	Patient 1																			
		Patient 2																			
	Placebo	Patient 3																			
. . .																					
Centre n	Verum	Patient 4																			
		. . .																			
		Patient 5																			
	Placebo	Patient 6																			
		. . .																			
		Patient 7																			

Listing 13 Serious Adverse Events Blood-culture proven sepsis

Centre	Treatment	Patient	Age in days	Sex	Weight*	Height	Description							Date of onset or date of clinic visit	Duration of treatment until date of onset	Duration of AE (in days)	CTCAE Severity	Causal relationship	Expected SAE	Action taken	Outcome
							SOC	HLGT	HLT	PT	LLT	Reported Term	AE of special interest								
Centre1	Verum	Patient 1																			

Centre	Treatment	Patient	Age in days	Sex	Weight*	Height	Description							Date of onset or date of clinic visit	Duration of treatment until date of onset	Duration of AE (in days)	CTCAE Severity	Causal relationship	Expected SAE	Action taken	Outcome
							SOC	HLGT	HLT	PT	LLT	Reported Term	AE of special interest								
		Patient 2																			
	Placebo	Patient 3																			
. . .																					
Centre n	Verum	Patient 4																			
		. . .																			
		Patient 5																			
	Placebo	Patient 6																			
		. . .																			
		Patient 7																			

Listing 14 Serious Adverse Events Clinical sepsis

Centre	Treatment	Patient	Age in days	Sex	Weight*	Height	Description							Date of onset or date of clinic visit	Duration of treatment until date of onset	Duration of AE (in days)	CTCAE Severity	Causal relationship	Expected SAE	Action taken	Outcome
							SOC	HLGT	HLT	PT	LLT	Reported Term	AE of special interest								
Centre1	Verum	Patient 1																			
		Patient 2																			
	Placebo	Patient 3																			
. . .																					
Centre n	Verum	Patient 4																			

Centre	Treatment	Patient	Age in days	Sex	Weight*	Height	Description							Date of onset or date of clinic visit	Duration of treatment until date of onset	Duration of AE (in days)	CTCAE Severity	Causal relationship	Expected SAE	Action taken	Outcome
							SOC	HLGT	HLT	PT	LLT	Reported Term	AE of special interest								
		. . .																			
		Patient 5																			
	Placebo	Patient 6																			
		. . .																			
		Patient 7																			

Listing 15 Serious Adverse Events Necrotizing enterocolitis

Centre	Treatment	Patient	Age in days	Sex	Weight*	Height	Description							Date of onset or date of clinic visit	Duration of treatment until date of onset	Duration of AE (in days)	CTCAE Severity	Causal relationship	Expected SAE	Action taken	Outcome
							SOC	HLGT	HLT	PT	LLT	Reported Term	AE of special interest								
Centre1	Verum	Patient 1																			
		Patient 2																			
	Placebo	Patient 3																			
. . .																					
Centre n	Verum	Patient 4																			
		. . .																			
		Patient 5																			
	Placebo	Patient 6																			
		.																			

		. .																			
		Patient 7																			

Listing 16 Serious Adverse Events Severe gastrointestinal complication

Centre	Treatment	Patient	Age in days	Sex	Weight*	Height	Description							Date of onset or date of clinic visit	Duration of treatment until date of onset	Duration of AE (in days)	CTCAE Severity	Causal relationship	Expected SAE	Action taken	Outcome
							SOC	HLGT	HLT	PT	LLT	Reported Term	AE of special interest								
Centre1	Verum	Patient 1																			
		Patient 2																			
	Placebo	Patient 3																			
. . .																					
Centre n	Verum	Patient 4																			
		. . .																			
		Patient 5																			
	Placebo	Patient 6																			
		. . .																			
		Patient 7																			

Listing 17 Patients with at least one Adverse Event

Centre	Treatment	Patient	Age in days	Sex	Weight*	Height	Description						Date of onset or date of clinic visit	Duration of treatment until date of onset	Duration of AE (in days)	CTCAE Severity	Causal relationship	Action taken	Outcome	Seriousness
							SOC	HLGT	HLT	PT	LLT	Reported Term								
Centre1	Verum	Patient 1																		
		Patient 2																		
	Placebo	Patient 3																		
. . .																				
Centre n	Verum	Patient 4																		
		. . .																		
		Patient 5																		
	Placebo	Patient 6																		
		. . .																		
		Patient 7																		

Listing 18 Patients with at least one Serious Adverse Event

Centre	Treatment	Patient	Age in days	Sex	Weight*	Height	Description							Date of onset or date of clinic visit	Duration of treatment until date of onset	Duration of AE (in days)	CTCAE Severity	Causal relationship	Expected SAE	Action taken	Outcome
							SOC	HLGT	HLT	PT	LLT	Reported Term	AE of special interest								
Centre1	Verum	Patient 1																			
		Patient 2																			
	Placebo	Patient 3																			
. . .																					
	Verum																				

Centre	Treatment	Patient	Age in days	Sex	Weight*	Height	Description							Date of onset or date of clinic visit	Duration of treatment until date of onset	Duration of AE (in days)	CTCAE Severity	Causal relationship	Expected SAE	Action taken	Outcome
							SOC	HLGT	HLT	PT	LLT	Reported Term	AE of special interest								
Centre n		Patient 4																			
		. . .																			
		Patient 5																			
	Placebo	Patient 6																			
		. . .																			
		Patient 7																			

15.4 Planned figures

15.4.1 Subject disposition

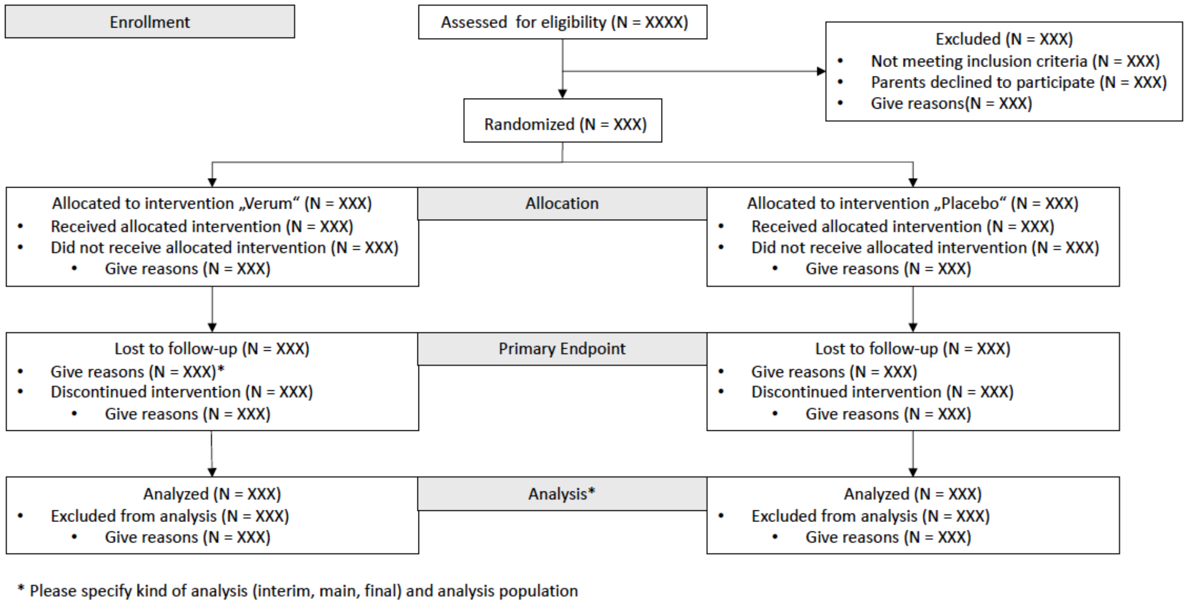

Figure 1 Disposition of patients according to CONSORT statement and ICH E3 Annex IVa and IVb

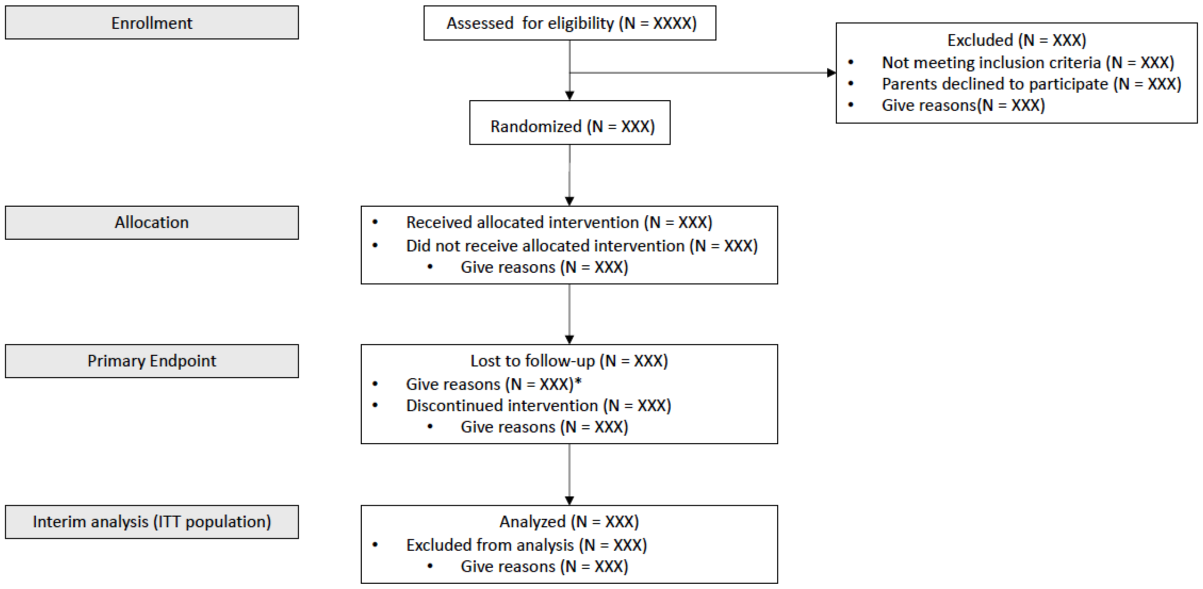

Figure 2 Disposition of patients according to CONSORT statement and ICH E3 Annex IVa and IVb: Blinded version for interim analysis

15.5 Data needed for secondary efficacy endpoint “gut dysbiosis”

Table 42 Variables needed for secondary efficacy endpoint gut dysbiosis. Mother data will only be provided fpor Mothers having signed informed consent version 4 and higher and data that is documented in the “Mutterpass”.

Variable name	Description
bl_geschlecht	Geschlecht (BL)
bl_einschluss_ssw	GA 28+ 0 bis 32 +6 SSW (BL)
bl_einschluss_gastro	Schwere gastrointestinale Fehlbildung (BL)
bl_nr_mehrling	Nummer der Mehrlings (BL)

Variable name	Description
bl_anzahl_mehrling	Anzahl der Mehrlings (BL)
bl_mehrling	Mehrling (BL)
bl_geburtsmodus	Geburtsmodus (BL)
bl_gewicht	Gewicht am Geburtstag (BL)
bl_laenge	Körperlänge am Geburtstag (BL)
bl_kopf	Kopfumfang am Geburtstag (BL)
bl_mutter_stat	Stationärer Aufenthalt der Mutter (BL)
bl_galter_ssw	GA (SSW) (BL)
bl_galter_tag	GA (Tag) (BL)
bl_mutter_antib	Antenatale Antibiotische Therapie (BL)
bl_mutter_einschr_andere	Ernährungeinschränkung andere (BL)
bl_mutter_herkunft_sonstige	Herkunft der Mutter sonstige (BL)
bl_mutter_einschr	Ernährung: Einschränkung (BL)
bl_mutter_einschr_art	Ernährung: Einschränkung welche (BL)
bl_mutter_raucher	Raucherin? (BL)
bl_mutter_exraucher	Exraucherin? (BL)
bl_mutter_herkunft	Herkunft der Mutter (BL)
t31_gewicht	Gewicht (T31)
t31_laenge	Körperlänge (T31)
t31_kopf	Kopfumfang (T31)
t31_dysbiose	Darm-Dysbiose (T31)
t31_antib	Antibiotische Therapie (T31)
t31_enteral_nahrung	Enteraler Nahrunsaufbau (T31)
t31_colostrum_oral	Colostrum oral (T31)
t31_colostrum_nahr	Colostrum als Nahrung (T31)
t31_erster_still	Erster Stillversuch (T31)
t31_eisen	Nahrungszusatz Eisen (T31)
t31_nahr_weitere	weiterer Nahrungszusatz (T31)
t31_gabe_abbruch_tag	Gabeabbruch an welchen Tag (1-28) (T31)
t31_colostrum_milch_1_30	Colostrum / eigene Muttermilch 1-30 LT (T31)
t31_colostrum_milch_1_30_pasteur	Colostrum / eigene Muttermilch pasteurisiert 1-30 LT (T31)

Variable name	Description
t31_sp_milch_1_30	Spenderinnenmilch 1-30 LT (T31)
t31_sp_milch_1_30_pasteur	Spenderinnenmilch 1-30 LT pasteurisiert? (T31)
t31_formula_1_30	Formula-Nahrung 1-30 T (T31)
t31_gabe_abbruch	Gabeabbruch (T31)
ent_sepsis1_blut_err_dat	Sepsis / SIRS: 1 Erregerdatum (ENT)
ent_sepsis2_blut_err_dat	Sepsis / SIRS: 2 Erregerdatum (ENT)
ent_sepsis3_blut_err_dat	Sepsis / SIRS: 3 Erregerdatum (ENT)
ent_sepsis4_blut_err_dat	Sepsis / SIRS: 4 Erregerdatum (ENT)
ent_sepsis5_blut_err_dat	Sepsis / SIRS: 5 Erregerdatum (ENT)
ent_sepsis6_blut_err_dat	Sepsis / SIRS: 6 Erregerdatum (ENT)
ent_sepsis_andere1_err_txt	Sepsis / SIRS: anderer Erreger1 (ENT)
ent_sepsis_andere1_err_dat	Sepsis / SIRS: anderes Erregerdatum1 (ENT)
ent_sepsis_andere2_err_txt	Sepsis / SIRS: anderer Erreger2 (ENT)
ent_sepsis_andere2_err_dat	Sepsis / SIRS: anderes Erregerdatum2 (ENT)
ent_sepsis_andere3_err_txt	Sepsis / SIRS: anderer Erreger3 (ENT)
ent_sepsis_andere3_err_dat	Sepsis / SIRS: anderes Erregerdatum3 (ENT)
ent_sepsis	Sepsis / SIRS (Kriterien NEO-KISS) (ENT)
ent_sepsis_72	Sepsis / SIRS: Beginn innerhalb 72h (ENT)
ent_sepsis_blut_err	Erreger in Blutkultur? (ENT)
ent_sepsis1_blut_err_nr	Sepsis / SIRS: 1 Erregernummer (ENT)
ent_sepsis2_blut_err_nr	Sepsis / SIRS: 2 Erregernummer (ENT)
ent_sepsis3_blut_err_nr	Sepsis / SIRS: 3 Erregernummer (ENT)
ent_sepsis4_blut_err_nr	Sepsis / SIRS: 4 Erregernummer (ENT)
ent_sepsis5_blut_err_nr	Sepsis / SIRS: 5 Erregernummer (ENT)
ent_sepsis6_blut_err_nr	Sepsis / SIRS: 6 Erregernummer (ENT)
ent_sepsis_andere_err	Sepsis / SIRS: anderer Erreger (ENT)
ent_kompl	Komplikationen / Operationen (ENT)
ent_pneumonie	Pneumonie (ENT)
ent_nec_op	NEC OP (ENT)
ent_fip_op	FIP OP (ENT)
ent_ivh	IVH (ENT)

Variable name	Description
ent_PVL	PVL (ENT)
ent_milch	Eigene Muttermilch (ENT)
ent_haus	Nach Hause entlassen? (ENT)
ent_tod	Tod in Klinik (ENT)
