# Supplementary material for: Bifidobacterium and Lactobacillus Probiotics and Gut Dysbiosis in Preterm Infants: The PRIMAL Randomized Clinical Trial
Source: JAMA Pediatr. 2024 Aug 5;178(10):985–95. doi: 10.1001/jamapediatrics.2024.2626 (PMC12549143; doi:10.1001/jamapediatrics.2024.2626)
Supplement: Supplement 3. — eTable 1. Probiotic Bacteria Reference Genomes eTable 2. MDRO+ Pathogens Reporting Criteria eTable 3. PERMANOVA 16S BC-Distance eTable 4. Variables Tested in MetadeconfoundR eTable 5. Software and Databases eTable 6. ProTection SNVs for B. Infantis Probiotic Strain eTable 7. Screened Pathogens by mOTU ID eTable 8. Data for Modeling eTable 9. Maternal Data eTable 10. Treatments and Continuous Outcomes of the Study Population eTable 11. Interim Analysis eTable 12. Reporting of Adverse Events eTable 13. Probiotic Strain (PS) Presence at Day 30 by Treatment Group eTable 14. Logistic Regression Model With Hospital as a Random Effect eTable 15. Secondary Analysis of Outcomes by Presence of Probiotic B. Infantis at Day 30 eTable 16. Number of Enrolled Infants per Site eFigure 1. Study Concept of the Multicentre Phase 3 PRIMAL Trial eFigure 2. Model of Eubiosis Based on Published Metagenomes eFigure 3. Enrollment, Randomization, and ITT Population of the Interim Analysis eFigure 4. Central MDRO+ Assessment of Day30 Samples According to Intervention eFigure 5. Environmental Uptake of Probiotic Strains eFigure 6. Microbiome Variability and Impact of Verum Treatment eAppendix 1. Study Efficacy Outcomes eAppendix 2. Statistical Analysis and Biostatistics for Microbiome Analysis eAppendix 3. Microbiome Composition Analysis eAppendix 4. Eubiosis Score Modeling eAppendix 5. EMMA Full-Term Cohort eAppendix 6. Environmental Uptake of Probiotic Strains eAppendix 7. Bifidobacterium Relative Abundance eReferences [file jamapediatr-e242626-s003.pdf]

Supplementary Online Content

Van Rossum T, Haiß A, Knoll RL, et al. *Bifidobacterium* and *Lactobacillus* probiotics and gut dysbiosis in preterm infants: the PRIMAL randomized clinical trial. *JAMA Pediatr*. Published online August 5, 2024. doi:10.1001/jamapediatrics.2024.2626

eTable 1. Probiotic Bacteria Reference Genomes

eTable 2. MDRO+ Pathogens Reporting Criteria

eTable 3. PERMANOVA 16S BC-Distance

eTable 4. Variables Tested in MetadeconfoundR

eTable 5. Software and Databases

eTable 6. ProTection SNVs for *B. Infantis* Probiotic Strain

eTable 7. Screened Pathogens by mOTU ID

eTable 8. Data for Modeling

eTable 9. Maternal Data

eTable 10. Treatments and Continuous Outcomes of the Study Population

eTable 11. Interim Analysis

eTable 12. Reporting of Adverse Events

eTable 13. Probiotic Strain (PS) Presence at Day 30 by Treatment Group

eTable 14. Logistic Regression Model With Hospital as a Random Effect

eTable 15. Secondary Analysis of Outcomes by Presence of Probiotic *B. Infantis* at Day 30

© 2024 Van Rossum T et al. *JAMA Pediatrics.*

eTable 16. Number of Enrolled Infants per Site

eFigure 1. Study Concept of the Multicentre Phase 3 PRIMAL Trial

eFigure 2. Model of Eubiosis Based on Published Metagenomes

eFigure 3. Enrollment, Randomization, and ITT Population of the Interim Analysis

eFigure 4. Central MDRO+ Assessment of Day30 Samples According to Intervention

eFigure 5. Environmental Uptake of Probiotic Strains

eFigure 6. Microbiome Variability and Impact of Verum Treatment

eAppendix 1. Study Efficacy Outcomes

eAppendix 2. Statistical Analysis and Biostatistics for Microbiome Analysis

eAppendix 3. Microbiome Composition Analysis

eAppendix 4. Eubiosis Score Modeling

eAppendix 5. EMMA Full-Term Cohort

eAppendix 6. Environmental Uptake of Probiotic Strains

eAppendix 7. Bifidobacterium Relative Abundance

eReferences

This supplementary material has been provided by the authors to give readers additional information about their work.

© 2024 Van Rossum T et al. *JAMA Pediatrics.*

eTable 1: Probiotic bacteria reference genomes

Genome name abbreviation	Genus	Species	Subspecies or Strain Name	Closest reference genome (nucleotide differences)
PI.Binf<br>*B. infantis* PS	*Bifidobacterium*	*B. longum*	infantis	NZ_LR134354.1<br>(203/2832748)"
PI.Bani<br>*B. animalis* PS	*Bifidobacterium*	*B. animalis*	BB-12	NZ_CP031703.1<br>(172/1944141)"
PI.Laci<br>*L. acidophilus* PS	*Lactobacillus*	*L. acidophilus*	LA-5	NZ_CP010432.1<br>(1/1991969)"

Abbrevations: PI Probiotic Intervention, PS Probiotic strain.

© 2024 Van Rossum T et al. *JAMA Pediatrics.*

eTable 2: MDRO+ pathogens reporting criteria of the central laboratory[a]

Genus	Species		Reporting Criteria
Staphylococcus	*aureus*		always
Serratia	*marcescens*		always
Morganella	*morganii*		if >= 2MRGN
Klebsiella	*pneumoniae*		always
Klebsiella	*oxytoca*		always
Escherichia	*coli*		if >= 2MRGN
Enterobacter	*cloacae*		if >= 2MRGN
Enterobacter	*aerogenes*		if >= 2MRGN
Citrobacter	*freundii*		if >= 2MRGN
Acinetobacter	*baumannii*		if >= 2MRGN

[a] Institute for Medical Microbiology and Hygiene of the University Medical Center Mainz, Germany.
Abbrevations: MDRO+ MultiDrug-Resistant Organisms or highly epidemic bacteria, MRGN Multidrug-Resistant Gram-Negative bacteria.

© 2024 Van Rossum T et al. *JAMA Pediatrics.*

eTable 3: PERMANOVA 16S Bray-Curtis-distance

**1. PERMANOVA without confounders Day 30**					
	Df	SumOfSqs	R2	F	Pr(>F)
**a) Treatment group (AT)**					
treatmentGroup_AT	1	5.293.285	0.04026473	2.248.734	0.001
Residual	536	126.168.781	0.95973527	NA	NA
Total	537	131.462.066	1	NA	NA
**b) Treatment or treatment of sibling (AT)**					
infantOrSiblingHadProbiotic	1	6.931.801	0.05390201	3.008.173	0.001
Residual	528	121.668.241	0.94609799	NA	NA
Total	529	128.600.041	1	NA	NA
**c) Bifidobacterium abundance**					
Bifidobacterium_relab	1	2.162.715	0.1645124	1.055.416	0.001
Residual	536	10.983.492	0.8354876	NA	NA
**2. PERMANOVA including potential confounders Day 30 (eFigure 5 D)**					
	Df	SumOfSqs	R2	F	Pr(>F)
infantOrSiblingHadProbiotic	1	6.447.933	0.0516623	30.256.883	0.001
hospitalID	17	78.270.782	0.062712324	2.160.501	0.001
sex	1	0.4993783	0.004001132	2.343.329	0.018
birth_deliveryMode	1	0.5713541	0.004577819	2.681.076	0.004
birth_ga_inDays	1	17.385.741	0.013929849	8.158.247	0.001
abx_mother_yn	1	0.2186702	0.001752035	1.026.108	0.402
abx_d31_nDays_DOL15toCollection	1	0.6004063	0.004810591	2.817.403	0.005
feeding_breastmilkAny_d1to31_yn	1	0.4377421	0.003507289	2.054.102	0.032
collection_DOL	1	0.589832	0.004725867	2.767.783	0.006
weight_d31_gain	1	0.382989	0.003068595	1.797.173	0.061
Residual	490	1.044.220.954	0.836653493	NA	NA
Total	516	1.248.092.505	1	NA	NA

Legend: Abbrevations: Df Degrees of freedom, SumOfSqa Sum Of Squares, R multivariate correlation coefficient of determination, F ratio of variances in Permanova, Pr p value of F, ga gestational age, abx antibiotics, yn yes/no, DOL Day Of Life

To address potential confounding effects of other microbiome shaping factors from infant clinical metadata on *B. infantis* abundance, a univariate correlation analysis was conducted using MetadeconfoundR (Version 0.2.8., for details see **Supplement 1**). The clinical metadata variables considered as potential confounders and tested are summarized in **eTable 4**. The same statistical approach was used to assess co-correlations of the probiotic-assigned amplicon sequence variants (ASVs) with other genera. To determine the ASVs that

© 2024 Van Rossum T et al. *JAMA Pediatrics.*

correspond to our probiotic insstrains, we extracted the 16S rRNA gene regions from the genomic data produced by sequencing the probiotic material using barrnap (v0.9) and bedtools (v2.27.1).

eTable 4: Variables tested in MetadeconfoundR

**Variables tested in MetadeconfoundR**		
treatmentGroup_AT	medication_end_DOL	abx_d31_yn
dna_ng_per_mg_stool	medication_notGiven_nDays	abx_mother_yn
hospitalID	medication_notGiven_nDays15to31	abx_d31_nDays_DOL0toCollection
sex	dysbiosis_krinko_d31_mainz	abx_d31_nDays_DOL15toCollection
birth_deliveryMode	dysbiosis_krinko_d31_local	collection_nDaysPostMedication
birth_bonding_yn	feeding_enteral_startDayOfLife	collection_DOL
birth_bonding_mouthChestContact_yn	feeding_enteral_d31_numDays	collection_nDaysPostAbx
birth_kangarooing_yn	feeding_supplements_d31_yn	mother_smoker_yn
birth_kangarooing_dayOfLife	feeding_breastmilkAny_d1to31_yn	mother_smokerFormer_yn
ageCat	feeding_breastmilkScore_d1to31	EOS
multipleBirth_yn	breathing_ventilation_d31_yn	LOS
weight_birth	breathing_cpap_d31_numDays	infantOrSiblingHadProbiotic
weight_d31	breathing_cpap_d31_yn	staffing_propShifts_d0to31
weight_d31_gain	breathing_highflow_d31_numDays	roommates_meanNum_1infantPerNurse_d0to31
medication_terminatedEarly_yn	breathing_highflow_d31_yn	roommates_meanNum_2infantsPerNurse_d0to31

Abbrevations: AT As Treated population, yn yes/no, DOL Day Of Life, cpap continuous positive airway pressure, abx antibiotics, EOS Early-Onset Sepsis, LOS Late-Onset Sepsis, dysbiosis_krinko means microbiological culture and identification of multidrug-resistant strains (multiresistant gram-negative bacteria (MRGN); 2 MRGN; 3 MRGN; 4 MRGN; methicillin-resistant *Staphylococcus aureus)* and/or pathogens without resistance characteristics but highly epidemic potential for outbreaks (*Serratia* spp; *Pseudomonas* spp; *Klebsiella* spp; *Enterobacter* spp) according to German screening recommendations of the Kommission für Krankenhaushygiene und Infektionsprävention (KRINKO).

Legend: For each group, namely Bifidobacteriales and Lactobacillales, we conducted a multiple sequence alignment of amplicon sequence variants (ASVs) using muscle (v5.1). Subsequently, the consensus of these alignments, derived from cons (emboss v6.6.0), was blasted against the extracted probiotic 16S rRNA gene sequences via blastn (v2.12.0) to pinpoint the precise amplicon region of the isolates. These sequences, once concatenated, underwent another alignment phase, followed by the construction of a phylogenetic tree through raxml-ng (v1.2.0) (GTR+G model). The resultant tree was visualized using iTOL (v5). This process identified the ASVs to which the probiotic strains belong. Other related species and strains maybe also be represented by these ASVs. During calculations of correlations between the probiotic-corresonding ASVs and the other taxa, ASVs from Lactobacillus and Bifidobacterium were removed from the tested genera to avoid self-correlation. All softwares, databases and platforms are referenced in eTable 5.

© 2024 Van Rossum T et al. *JAMA Pediatrics.*

eTable 5: Software and databases used for microbiome sequencing and analysis

Software/Platform/Database	Reference
Blastn	Boratyn GM, Schäffer AA, Agarwala R, et al. 2012. Domain Enhanced Lookup Time Accelerated BLAST. Biology Direct. 2012;7:12. https://doi.org/10.1186/1745-6150-7-12.
barrnap (v0.9)	Seemann T. 2013. BAsic Rapid Ribosomal RNA Predictor (barrnap). https://github.com/tseemann/barrnap. ———. Last accessed October 1st 2023. ABRicate: Mass Screening of Contigs for Antimicrobial and Virulence Genes. https://github.com/tseemann/abricate.
bedtools (v2.27.1)	Seemann T. 2013. BAsic Rapid Ribosomal RNA Predictor (barrnap). https://github.com/tseemann/barrnap. ———. Last accessed October 1st 2023. ABRicate: Mass Screening of Contigs for Antimicrobial and Virulence Genes. https://github.com/tseemann/abricate.
cons	Rice P, Longden I, Bleasby A. EMBOSS: The European Molecular Biology Open Software Suite. Trends in Genetics 2000; TIG16(6):276–277. https://doi.org/10.1016/s0168-9525(00)02024-2."
DADA2	Callahan BJ, McMurdie PJ, Rosen MJ, et al. DADA2: High-Resolution Sample Inference from Illumina Amplicon Data. Nature Methods 2016;13(7):581–583. https://doi.org/10.1038/NMETH.3869.
eggNOG mapper	Huerta-Cepas J, Szklarczyk D, Forslund K, et al. 2016. eggNOG 4.5: A Hierarchical Orthology Framework with Improved Functional Annotations for Eukaryotic, Prokaryotic and Viral Sequences. Nucleic Acids Research 2016;44. https://doi.org/10.1093/nar/gkv1248.
GMGC	Coelho LP, Alves R, Rodríguez del Río A, et al. Towards the Biogeography of Prokaryotic Genes. Nature. 2022;601(7892):252–256. https://doi.org/10.1038/s41586-021-04233-4
iTOL	Kozlov AM, Darriba D, Flouri T, Morel B, Stamatakis A. RAxML-NG: A Fast, Scalable and User-Friendly Tool for Maximum Likelihood Phylogenetic Inference. Bioinformatics 2019;35(21):4453–4455. https://doi.org/10.1093/bioinformatics/btz305<br>Letunic I, Bork P. Interactive Tree Of Life (iTOL) v5: An Online Tool for Phylogenetic Tree Display and Annotation. Nucleic Acids Research 2021;49(W1):W293–296. https://doi.org/10.1093/nar/gkab301.
MetadeconfoundR (Version 0.2.8.)	Forslund SK, Chakaroun R, Zimmermann-Kogadeeva M,et al. Combinatorial, Additive and Dose-Dependent Drug–microbiome Associations. Nature 2021;600(7889):500–505. https://doi.org/10.1038/s41586-021-04177-9.<br>Birkner T. metadeconfoundR (version 0.2.8). 2021 https://github.com/TillBirkner/metadeconfoundR.
MetaPhlAn4mpa_vJan21_ CHOCOPhlAnSGB_202103) using	Blanco-Míguez A, Beghini F, Cumbo F, et al. Extending and Improving Metagenomic Taxonomic Profiling with Uncharacterized Species Using MetaPhlAn 4. Nature Biotechnology. 2023 https://doi.org/10.1038/s41587-023-01688-w.
Muscle	Edgar RC. MUSCLE: Multiple Sequence Alignment with High Accuracy and High Throughput. Nucleic Acids Research.2004;32(5):1792–1797. https://doi.org/10.1093/nar/gkh340.
ngless v1.1	Coelho LP, Alves R, Monteiro P, Huerta-Cepas J, Freitas AT, Bork P. NG-Meta-Profiler: Fast Processing of Metagenomes Using NGLess, a Domain-Specific Language. Microbiome. 2019;7(1):1–10. https://doi.org/10.1186/s40168-019-0684-8.
PATRIC	Wattam AR, Davis JJ, Assaf R, et al. Improvements to PATRIC, the All-Bacterial Bioinformatics Database and Analysis Resource Center. Nucleic Acids Research. 2017;45(D1):D535–542. https://doi.org/10.1093/nar/gkw1017.
ProTection metaSNV2	Van Rossum T, Costea PI, Paoli L, Alves R, Thielemann R, Sunagawa S, Bork P. metaSNV v2: Detection of SNVs and Subspecies in Prokaryotic Metagenomes. Bioinformatics. 2022;38(4):1162–1164. https://doi.org/10.1093/bioinformatics/btab789."

© 2024 Van Rossum T et al. *JAMA Pediatrics.*

Software/Platform/Database	Reference
Resfinder	Kozlov AM, Darriba D, Flouri T, Morel B, Stamatakis A. RAxML-NG: A Fast, Scalable and User-Friendly Tool for Maximum Likelihood Phylogenetic Inference. Bioinformatics. 2019;35(21):4453–4455. https://doi.org/10.1093/bioinformatics/btz305.
Resfinder	Zankari E, Hasman H, Cosentino S, et al. Identification of Acquired Antimicrobial Resistance Genes. The Journal of Antimicrobial Chemotherapy. 2012;67(11): 2640–2444. https://doi.org/10.1093/jac/dks261
SameStr	Podlesny D, Arze C, Dörner E, et al. Metagenomic Strain Detection with SameStr: Identification of a Persisting Core Gut Microbiota Transferable by Fecal Transplantation. Microbiome. 2021. https://doi.org/10.1186/s40168-022-01251-w.
SILVA	Quast C, Pruesse E, Yilmaz P, et al. The SILVA Ribosomal RNA Gene Database Project: Improved Data Processing and Web-Based Tools. Nucleic Acids Research. 2013;41(D1):590–596. https://doi.org/10.1093/nar/gks1219.
SPAdes	Prjibelski A, Antipov D, Meleshko D, Lapidus A, Korobeynikov A. Using SPAdes De Novo Assembler. Current Protocols in Bioinformatics / Editoral Board, Andreas D. Baxevanis ... [et Al.] 2020;70(1). https://doi.org/10.1002/cpbi.102."
Virulence factor database VFDB	Chen L, Zheng D, Liu B, Yang J, Jin Q. VFDB 2016: Hierarchical and Refined Dataset for Big Data analysis—10 Years on. Nucleic Acids Research. 2015;44(D1):D694–697. https://doi.org/10.1093/nar/gkv1239.

© 2024 Van Rossum T et al. *JAMA Pediatrics.*

eTable 6: ProTection SNVs for *B. infantis* probiotic strain

SNV position in reference genome (NZ_LR134354.1)	Nucleotide change	Coverage depth (99% identity mapping)
642221	G -> A	97
1154752	G -> A	57
1861310	C -> A	91
2114171	T -> C	70
2148691	G -> C	66
2781000	G -> A	98

Filtered single nucleotide variants (SNVs) with potential for the strain tracking approach. SNVs were called for the probiotic reads mapped against the closest reference genome. Strain-identifying SNVs had to have a coverage depth of at least 10 and a SNV allele frequency of 100%. The ID of the reference genome (NCBI database) is NZ_LR134354.1 for *B. infantis*.

Legend eTable6: ProTection was used to reliably detect the probiotic strain of *B. infantis* due to its high abundance in many samples and distinctive SNVs collected across the entire genome. The results agreed well with a parallel analysis using SameStr, which is limited to SNV-detection in species-specific marker genes but additionally reliably detected the probiotic *B. animalis* and *L. acidophilus* strains. For the SameStr analysis, sequences from each sample were first mapped to the MetaPhlAn clade-specific marker gene database (mpa_vJan21_CHOCOPhlAnSGB_202103) using MetaPhlAn4. The resulting alignments were then processed with SameStr, converting them into single nucleotide variant (SNV) profiles. These profiles were merged and filtered with default settings to identify strains when species alignments overlapped by ≥ 5 kb and had an MVS of ≥ 99.9%.
HMO genes were detected by mapping the metagenomic reads against the GMGC database and annotating the resulting hits with functional groups using eggNOG mapper. The relevant functional groups were previously identified.[4] Potential pathogens relevant to preterm infants to be detected by metagenomic species profiles were compiled from guidelines recommendations for MDRO+ screening and from the literature (**eTable 7**). Presence and relative abundance of these pathogens were detected from the mOTUs species profiles. Each species' abundances were normalised across infants (Z score) and summed per infant to give a single pathogen abundance measure per sample, which was not dominated by any one species with a higher level of background abundance. Bacterial-gene mediated antimicrobial drug resistances and virulence factors were detected using abricate (https://github.com/tseemann/abricate) and the resfinder and the virulence factor (VFDB) databases, respectively.

© 2024 Van Rossum T et al. *JAMA Pediatrics.*

eTable 7: Screened pathogens by mOTU ID

Species name	mOTU ID
Citrobacter sp.	ref_mOTU_v25_00103
Citrobacter sp.	ref_mOTU_v25_00096
Citrobacter sp. MGH106	ref_mOTU_v25_00097
Escherichia coli	ref_mOTU_v25_00095
Klebsiella michiganensis/oxytoca	ref_mOTU_v25_00086
Klebsiella pneumoniae	ref_mOTU_v25_00085
Serratia marcescens	ref_mOTU_v25_00830
Staphylococcus aureus	ref_mOTU_v25_00340
Staphylococcus aureus/haemolyticus	ref_mOTU_v25_00343
Staphylococcus epidermidis	ref_mOTU_v25_00346
Staphylococcus lugdunensis	ref_mOTU_v25_01063
Staphylococcus warneri/capitis	ref_mOTU_v25_00348
Streptococcus agalactiae	ref_mOTU_v25_01860
Streptococcus agalactiae	ref_mOTU_v25_01861
Streptococcus agalactiae	ref_mOTU_v25_01862

Abbrevation: mOTU Microbial Operational Taxonomic Unit.

© 2024 Van Rossum T et al. *JAMA Pediatrics.*

eTable 8: Data for modeling[a]

Study Accession	PubMed ID	Model Group	Sample Accession
PRJNA473126	30374198	full-term	SAMN09259845
PRJNA473126	30374198	full-term	SAMN09259852
PRJNA473126	30374198	full-term	SAMN09259884
PRJNA473126	30374198	full-term	SAMN09259891

[a]This table introduces the first 4 out of 492 samples used in model building, presenting the study accession number along with their unique sample identifiers.The full list is available at the following link: https://docs.google.com/spreadsheets/d/1QBIafKZoJbRVG3f-sRXvWbHtsPIjf7zqx9TDBl66sng/edit?usp=sharing.

Legend eTable 8: The model produces a score, from 0 to 1, with higher values reflecting a higher probability of a eubiotic (healthy) state. The model was made with species and genus profilesBoth models easily distinguished preterm infant microbiomes from healthy, full-term microbiomes, with an accuracy of >90% and an AUC of >0.93 under cross validation grouped by study. The most important predictors in the species model were an unnamed Streptococcus species, *Bifidobacterium longum* (the species to which *B. infantis* belongs), *Bacteroides dorei/vulgatus, Clostridioides difficile, Streptococcus salivarius, Rothia mucilaginosa,* and *Bacteroides rodentium/uniformis*. The most important predictors in the genus model were *Bacteroides, Rothia* and *Bifidobacterium* (**eFigure 2**). These models were applied to the PRIMAL Day 30 fecal microbiomes to assess how similar those microbiomes were to typical preterm and full-term microbiomes. The genus-resolution model was applied to the 16S data, and the species-resolution model was applied to the subset of samples with metagenomic data.

To assess whether differences in eubiosis scores were due to the presence of the probiotic bacteria themselves or due to other community composition differences, a second set of models was trained. Here, the modeling described above was repeated, but the probiotic taxa (*B. longum*, *B. animalis*, and *L. acidophilus*) were excluded from model building. The resulting species and genus models distinguish appropriately between the "preterm" and "full-term" groups (accuracy > 88%, AUC > 0.92). The most important taxa for these models are the same as the previous models, without *Bifidobacterium*. These models were then also applied to the PRIMAL Day 30 fecal microbiomes.

© 2024 Van Rossum T et al. *JAMA Pediatrics.*

eTable 9: Maternal data

Demographic characteristics	**Total**		**Verum**		**Control**	
	n<br>%<br>Mean (SD)	n[a]	n<br>%<br>Mean (SD)	n[a]	n<br>%<br>Mean (SD)	n[a]
Maternal data						
Origin of mother						
Germany (%)	480 (78.7)	610	243 (80.20%)	303	237 (77.20%)	307
Asia (%)	9 (1.5)	610	6 (1.98%)	303	3 (0.98%)	307
Africa (%)	14 (2.3)	610	6 (1.98%)	303	8 (2.61%)	307
Rest of Europe including Russia (%)	60 (9.9)	610	23 (7.59%)	303	37 (12.05%)	307
East, Turkey and North Africa (%)	35 (5.7)	610	18 (5.94%)	303	17 (5.54%)	307
Other (%)	12 (2.0)	610	7 (2.31%)	303	5 (1.6%)	307
Age (years, median/SD)	32.4 (±5.5)	615	32.6 (±5.5)	304	32.2 (±5.6)	311
Body mass index (kg/m^2, median/SD)	25.5 (±5.7)	570	25.7 (±5.7)	280	25.3 (±5.7)	290
Nutritional restriction (%)	46 (7.8)	592	25 (8.6)	291	21 (7.0)	301
Diet vegetarian (%)	14 (36.8)	38	7 (35.0)	20	7 (38.9)	18
Diet vegan (%)	1 (2.6)	38	-	20	1 (5.6)	18
Other nutritional restriction (%)	23 (60.5)	38	13 (65.0)	20	10 (55.6)	18
Smoking (%)	61 (10.3)	593	32 (11.0)	292	29 (9.6)	301
If non-smoker, ex-smoker (%)	146 (33.6)	434	72 (34.8)	207	74 (32.6)	227
Highest educational level						
High school degree (%)	262 (47.9)	547	122 (45.5)	268	140 (50.2)	279
Secondary school degree (%)	206 (37.7)	547	97 (36.2)	268	109 (39.1)	279
Other/No school degree (%)	79 (14.4)	547	49 (18.3)	268	30 (10.8)	279
Antenatal antibiotics	286 (46.8)	611	143 (47.2)	303	143 (46.4)	308
Ampicillin/other Penicillins	115 (40.5)	284	56 (39.4)	142	59 (41.6)	142
Cephalosporins	155 (54.6)	284	77 (54.2)	142	78 (54.9)	142
Macrolides	63 (22.2)	284	32 (22.5)	142	31 (21.8)	142
Carbapenems	14 (4.9)	284	8 (5.6)	142	6 (4.2)	142
Metronidazol	69 (24.3)	284	32 (22.5)	142	37 (26.1)	142
Other	68 (23.9)	284	34 (23.9)	142	34 (23.9)	142

[a]number of mothers with available data.
Abbrevation: SD Standard Deviation.

© 2024 Van Rossum T et al. *JAMA Pediatrics.*

eTable 10: Treatments and continuous outcomes of the study population

		Total		Verum		Placebo	
		n (%) Median (IQR)	n[a]	n (%) Median (IQR)	n[a]	n (%) Median (IQR)	n[a]
**Antiinfective treatment**		397 (64.2)	618	201 (65.7)	306	196 (62.8)	312
Total days of antiinfectives		5 (3-7)	395	5 (3-7)	201	5 (3-7)	194
Number of antiinfective courses		1 (1-1)	395	1 (1-1)	201	1 (1-1)	194
Antiinfective[b]	Ampicillin/Amoxicillin	217 (54.8)	396	110 (55.0)	200	107 (54.6)	196
	Ampicillin/Sulbactam	131 (33.1)	396	65 (32.5)	200	66 (33.6)	196
	Cefotaxim	55 (13.9)	397	32 (15.9)	201	23 (11.7)	196
	Ceftazidim	5 (1.3)	397	3 (1.5)	201	2 (1.0)	196
	Cefuroxim	5 (1.3)	397	3 (1.5)	201	2 (1.0)	196
	Clarithromycin	34 (8.6)	397	17 (8.5)	201	17 (8.7)	196
	Erythromycin	6 (1.5)	397	5 (2.5)	201	1 (0.5)	196
	Fluconazol	13 (3.3)	397	8 (4.0)	201	5 (2.6)	196
	Gentamicin	287 (72.3)	397	147 (73.1)	201	140 (71.4)	196
	Meropenem	27 (6.8)	397	13 (6.5)	201	14 (7.1)	196
	Piperacillin/Tazobact.	27 (6.8)	397	12 (6.0)	201	15 (7.7)	196
	Tobramycin	71 (17.9)	397	36 (17.9)	201	35 (17.9)	196
	Vancomycin	20 (5.0)	397	9 (4.5)	201	11 (5.6)	196
	Teicoplanin	39 (9.8)	397	18 (9.0)	201	21 (10.7)	196
**Nutrition**							
Start of enteral feeding on day 1		618 (100)	618	306 (100)	306	312 (100)	312
Time to full enteral feeding in days (up to 150 ml/kg/d)		10 (7.5)	592	10 (8)	292	10 (7)	300
Colostrum oral		420 (68.2)	616	203 (66.3)	306	217 (70.0)	310
Colostrum / mother's own milk (MoM) until Day 30		560 (90.8)	617	283 (92.8)	305	277 (88.8)	312
	Pasteurized MoM	71 (12.8)	555	37 (13.2)	280	34 (12.4)	275
Donor human milk (DHM)		107 (17.3)	617	56 (18.4)	305	51 (16.4)	312
	Pasteurized DHM	50 (47.6)	105	24 (42.9)	56	26 (53.1)	49
Formula		479 (77.8)	616	242 (79.6)	304	237 (76.0)	312
Exclusive human milk feeding until Day 30		136 (22.4)	616	62 (20.3)	304	74 (23.7)	312
Umbilical venous or central line		286 (46.3)	618	146 (47.7)	306	140 (44.9)	312
Days of central line		10 (6)	286	10 (7)	146	10 (6)	140
Days of peripheral line		6 (5)	115	6 (4)	58	6 (5)	57
**Respiratory support**							
invasive mechanical ventilation[c]		103 (18.5)	557	49 (17.4)	281	54 (19.6)	276
non invasive support (CPAP, HFNC)		454 (81.5)	557	227 (80.8)	281	227 (82.3)	276
Days on CPAP		7 (10)	547	7 (10)	270	6 (11)	277
Days on HFNC		7 (10)	221	6 (10)	113	9.5 (10)	108
Supplemental oxygen		557 (90.1)	618	276 (90.2)	306	281 (90.1)	312
days on supplemental oxygen[d]		9 (17)	556	9 (15)	276	9 (18)	280
Duration of hospital stay		41(24)	617	41(24)	306	41(25)	311

[a]number of non-missing values; [b]Amikacin, Cefazolin, Azithromycin, Metronidazol each n=1, Flucloxacillin n=2; [c]ventilated infants with missing values on CRF for 'invasive mechanical ventilation' were treated as "non invasively ventilated" (n=55); [d]calculated based on the entire period of oxygen supply, interim breaks were not considered;
Abbrevations: IQR InterQuartile Range, CPAP Continuous Positive Airway Pressure, HFNC High Flow Nasal Cannula.

© 2024 Van Rossum T et al. *JAMA Pediatrics.*

eTable 11: Interim analysis

Demographic characteristics		Total<br>n<br>%<br>Median (IQR)<br>Mean (SD)	n[a]	Verum<br>n<br>%<br>Median (IQR)<br>Mean (SD)	n[a]	Control<br>n<br>%<br>Median (IQR)<br>Mean (SD)	n[a]
Gender	Female	106 (47.3)	224	58 (50.0)	116	48 (44.4)	108
Multiple birth		95 (42.4)	224	48 (41.)	116	47 (43.5)	108
Gestational age (weeks)		31 (29.6 - 32.1)	224	31.1 (30.1 - 32.1)	116	31 (29.1 - 32)	108
Birth weight (g)		1487.6 (±361.1)	224	1494.7 (±347.1)	116	1480.1 (±377.0)	108
Length (cm)		40.4 (±3.5)	221	40.7 (±3.6)	115	40.0 (±3.5)	106
Head circumference (cm)		28.3 (±1.9)	223	28.5 (±2.0)	115	28.2 (±1.9)	108
Mode of birth	Spontaneous	39 (17.4)	224	19 (16.4)	116	20 (18.5)	108
	Caesarean section, elective	163 (72.8)	224	83 (71.5)	116	80 (74.1)	108
	Caesarean section, emergency	22 (9.8)	224	14 (12.1)	116	8 (7.4)	108
Cause of preterm birth	Preterm labour	109 (48.7)	224	57 (49.1)	116	52 (48.1)	108
	Preterm rupture of membranes	13 (5.8)	224	5 (4.3)	116	8 (7.4)	108
	Amniotic infection syndrome	23 (10.3)	224	9 (7.8)	116	14 (13.0)	108
	Pre-eclampsia	19 (8.5)	224	9 (7.8)	116	10 (9.3)	108
	HELPP-syndrome	14 (6.2)	224	9 (7.8)	116	5 (4.6)	108
	Pathological Doppler						
	Growth restriction	25 (11.2)	224	13 (11.2)	116	12 (11.1)	108
	Placental abruption	15 (6.7)	224	7 (6.0)	116	8 (7.4)	108
	Pathological cardiotocography	38 (17.0)	224	22 (19.0)	116	16 (14.8)	108
	Prolaps of membranes	8 (3.6)	224	4 (3.4)	116	4 (3.7)	108
	Rupture of membranes without anhydramnions	46 (20.5)	224	23 (19.9)	116	23 (21.3)	108
	Other	30 (13.4)	224	14 (12.1)	116	16 (14.8)	108
Antenatal steroids administered		192 (86.1)	223	94 (81.7)	115	98 (90.7)	108
	If yes completed cycle (2 doses, 12 h after 2. dose)	143 (69.8)	205	70 (68.6)	102	73 (70.9)	103

[a] number of infants with available data;
Abbrevations: SD Standard Deviation, IQR InterQuartile Range.

© 2024 Van Rossum T et al. *JAMA Pediatrics.*

eTable 12: Reporting of adverse events

			Total (n=638)	Verum[a] (n=316)	Placebo (n=322)
Serious and non-serious AEs	Number		197	115	82
	Median time in d (IQR)		8 (9)	8 (10)	7 (7)
	Severity	Mild	46	24	22
		Moderate	91	52	39
		Severe	41	27	14
		Life threatening consequences	19	12	7
		Death related to AE	1	1	-
	Potentially causal relationship to treatment	Related	10	6	4
		Not related	187	109	78
	Status/outcome of event	Recovered	163	95	68
		Recovered with sequel	7	2	5
		Not yet recovered, further treatment not necessary	12	7	5
		Not yet recovered, further treatment necessary	14	10	4
		Fatal	1	1	-
		Unknown	-	-	-
	Entities[b]	Blood disorders	4	3	1
		Cardiac problems	9	3	6
		Respiratory events	23	17	6
		Gastrointestinal events	24	15	9
		Infections	92	53	39
		Endocrine/metabolic	3	2	1
		Eye disorder	12	6	6
		Neurological event	20	12	8
		Renal complications	3	2	1
		Skin abnormalities	7	2	5
Non-serious AEs (n=85)	Number		88	49	39
	Median time in d (IQR)		7 (19)	9 (17)	6 (26)
	Severity	Mild	37	19	21
		Moderate	48	30	18
		Sever	-	-	-
		Life threatening consequences	-	-	-
		Death related to AE	-	-	-
	Potentially causal relationship to treatment	Related	6	4	2
		Not related	82	45	37
	Status / outcome of event	Recovered	65	36	29
		Recovered with sequel	3	1	2
		Not yet recovered, further treatment not necessary	12	7	5
		Not yet recovered, further treatment necessary	8	5	3
		Fatal	-	-	-
		Unknown	-	-	-
Serious AEs (n=114)	Number		109	66	43
	Median time in d (IQR)		8 (5)	8 (6)	7 (4)
	Severity	Mild	5	2	3
		Moderate	43	24	19
		Severe	41	27	14

© 2024 Van Rossum T et al. *JAMA Pediatrics.*

			Total (n=638)	Verum[a] (n=316)	Placebo (n=322)
SAE		Life threatening consequences	19	12	7
		Death related to AE	1	1	-
	Potentially causal relationship to treatment	Related	4	2	2
		Not related	105	64	41
	Status / outcome of event	Recovered	98	59	39
		Recovered with sequel	4	1	3
		Not yet recovered, further treatment not necessary	-	-	-
		Not yet recovered, further treatment necessary	6	5	1
		Fatal	1	1	-
		Unknown	-	-	-

[a]including an observation (12.1.8.004) with double treatment; [b] Entities: Blood disorder: anemia, low platelets, feto-fetal transfusion syndrome, coagulation disorder. Cardiac disorder: arrhythmia, patent Ductus arteriosus, edema. Respiratory events: pulmonary hypoplasia, bronchopulmonary dysplasia, pneumothorax (there were 9 verum-treated infants as compared to 2 placebo-treated infants with pneumothorax which was interpreted as chance observation), pneumonia, respiratory failure. Gastrointestinal events: pyloric stenosis, abdominal distension, gastric perforation, reflux, meconium plug, hematochezia, intestinal hemorrhage, inguinal hernia, necrotizing enterocolitis, focal intestinal perforation, volvulus, cholestasis. Infections: local infections, systemic infections (clinical or culture-proven), abnormal investigations (inflammation markers increased). Endocrine and metabolic events: hyperthyroidism, metabolic disorder. Eye disorders: retinopathy of prematurity, eye discharge. Neurological disorder: intraventricular hemorrhage, periventricular leukomalazia, hydrocephalus. Renal disorder: Nephrocalcinosis, renal impairment. Skin abnormalities: exfoliative dermatitis, necrosis, hemangioma.
Abbrevations: IQR InterQuartile Range, AE Adverse Event, SAE Serious Adverse Event.

© 2024 Van Rossum T et al. *JAMA Pediatrics.*

eTable 13: Probiotic strain (PS) presence at Day 30 by treatment group

Treatment Group (AT)	*B. infantis* PS detected	*B. animalis* PS detected	*L. acidophilus* PS detected	n
Placebo	NA	FALSE	FALSE	2
Placebo	FALSE	FALSE	FALSE	42
Placebo	FALSE	TRUE	FALSE	1
Placebo	TRUE	FALSE	FALSE	38
Placebo	TRUE	TRUE	FALSE	1
Placebo	TRUE	TRUE	TRUE	2
Probiotic	NA	TRUE	FALSE	2
Probiotic	TRUE	FALSE	FALSE	29
Probiotic	TRUE	FALSE	TRUE	7
Probiotic	TRUE	TRUE	FALSE	21
Probiotic	TRUE	TRUE	TRUE	43

Abbrevations: PS Probiotic Strain, NA Not Available.

© 2024 Van Rossum T et al. *JAMA Pediatrics.*

eTable 14: Logistic regression model with hospital as a random effect on presence of probiotic *B. infantis* at Day 30 group within the control group

Effect	group	term	esti-mate	std. error	statistic	p.value	conf. low	conf. high	estimate OR	conf. low_OR	conf. high_OR	Df residual	nObs	q.value _BH
fixed	NA	(Intercept)	0.53	1.64	0.32	0.75	-2.68	3.74	1.70	0.07	42.23	63	73	0.84
**fixed**	**NA**	**probioticExposureUnits**	**2.67**	**0.89**	**3.01**	**0.0026**	**0.94**	**4.41**	**14.49**	**2.55**	**82.40**	**63**	**73**	**0.02**
fixed	NA	collection_DOL	0.31	0.49	0.63	0.53	-0.65	1.27	1.36	0.52	3.55	63	73	0.75
fixed	NA	feeding_breastmilkAny_d1to31_ynTRUE	0.67	1.23	0.55	0.58	-1.74	3.09	1.96	0.18	21.89	63	73	0.75
fixed	NA	abx_d31_nDays_DOL0toCollection	-0.03	0.42	-0.06	0.95	-0.85	0.80	0.97	0.43	2.23	63	73	0.95
fixed	NA	birth_deliveryModeVaginal	0.94	1.49	0.63	0.53	-1.97	3.85	2.56	0.14	47.22	63	73	0.75
**fixed**	**NA**	**birth_ga_inDays**	**1.42**	**0.62**	**2.31**	**0.021**	**0.21**	**2.63**	**4.14**	**1.24**	**13.83**	**63**	**73**	**0.09**
fixed	NA	abx_mother_ynTRUE	-0.99	0.90	-1.10	0.27	-2.76	0.77	0.37	0.06	2.17	63	73	0.61
fixed	NA	sexMale	-1.05	0.92	-1.15	0.25	-2.85	0.74	0.35	0.06	2.11	63	73	0.61
ran _pars	Hospital ID	sd_(Intercept)	2.25	NA	NA	NA	NA	NA	2.25	NA	NA	63	73	NA

Abbrevations: NA Not Avaiable, OR Odds Ratio, Df degrees of freedom, nOBs number of Observations, BH Benjamini-Hochberg procedure.

© 2024 Van Rossum T et al. *JAMA Pediatrics.*

eTable 15: Secondary analysis of outcomes by presence of probiotic *B. infantis* at Day 30.

	Measure	Total N	*B. infantis* PS +	*B. infantis* PS -	Adjusted risk ratio (95% CI, exploratory p)[a]
Primary Outcome	Primary outcome: MDRO+ colonization Day 30, n (%)	184	49/141 (34.8)	16/43 (37.2)	0.95 (0.42-2.13, p=0.89)
Secondary Outcome	Secondary outcome Central MDRO+ colonization Day 30 n (%)	179	72/138 (52.2)	18/41 (43.9)	1.52 (0.73-3.14, p=0.39)
	Eubiosis model classification at bacterial genus resolution n (%)	179	81/137 (59.1)	8/42 (19.0)	5.94 (2.54-13.9, p<0.01)
	Eubiosis model score at bacterial genus resolution N, Median, IQR	179	137, 0.7, 0.47	42, 0.13, 0.52	1.43 (1.29-1.58, p<0.001)
	Eubiosis model classification at bacterial species resolution n (%)	179	83/137 (60.6)	6/42 (14.3)	9.35 (3.66-23.88, p<0.01)
	Eubiosis model score at bacterial species resolution N, Median, IQR	179	137, 0.84, 0.28	42, 0.37, 0.56	1.52 (1.41-1.63, p<0.001)
Additional measures	Abundance of *B. longum* species N, Median, IQR	179	137, 0.644, 0.294	42, 0, <0.0001	1.7 (1.59-1.84, p<0.001),
	Abundance of HMO metabolising genes (Z-score) N, Median, IQR	179	137, 0.49, 1.03	42, -1.37, 0.019	4.75 (3.75-6.01, p<0.001)
	Abundance of potential pathogens[b] (Z-score) N, Median, IQR	179	137, -1.4, 2.53	42, 0.6, 3.3	0.07 (0.02-0.26, p<0.001)
	Any antimicrobial resistance detected n (%)	179	19/137 (13.9)	8/42 (19.1)	0.78 (0.25-2.44, p=0.99)
	Any bacterial virulence factors detected n (%)	184	20/141 (14.2)	8/43 (18.6)	0.9 (0.28-2.83, p=0.99)

[a]Estimated in a generalized linear mixed model adjusted for site, sex, and age; [b] full list of screened pathogens in **eTable 7**.
Abbrevations: PS + Probiotic Strain positive, PS - Probiotic Strain negative, CI Confidence Interval, IQR InterQuartile Range, MDRO+ MultiDrug-Resistant Organisms or highly epidemic bacteria

© 2024 Van Rossum T et al. *JAMA Pediatrics.*

eTable 16 Number of enrolled infants per site

Studysite	No of enrolled children
Department of Pediatrics, University Hospital Schleswig-Holstein, Campus Lübeck, Germany	115
Department of General Pediatrics and Neonatology, Saar University Homburg, Germany	82
Department of Neonatology, Allergology and Pediatric Pneumology, Hannover Medical School, Germany	64
Department of Neonatology, University of Heidelberg, Germany	64
Department of Pediatrics, University of Freiburg, Germany	42
Department of Neonatology, Hospital Rostock Südstadt, University of Rostock, Germany	36
Children's Hospital Paderborn, Germany	33
Children's Hospital Hamburg Wilhelmstift and Marien-Hospital Hamburg, Medical School Hamburg, Germany	30
Children's Hospital Mitte Bremen, Germany	29
Department of Neonatology, University of Jena, Germany	24
Department of Neonatology, University of Bonn, Germany	21
Department of Pediatrics I, University of Duisburg-Essen, Germany	21
Helios Children's Hospital Schwerin, Germany	20
Children's Hospital Aschaffenburg-Alzenau, Germany	19
Children's Hospital Horst-Schmidt-Kliniken Wiesbaden, Germany	16
Department of Pediatrics, University of Cologne, Germany	15
Department of Pediatrics, University of Bochum, Germany	8
Department of Neonatology, University of Tübingen, Germany	7

© 2024 Van Rossum T et al. *JAMA Pediatrics.*

eFigure 1: Study concept of the multicentre Phase 3 PRIMAL trial

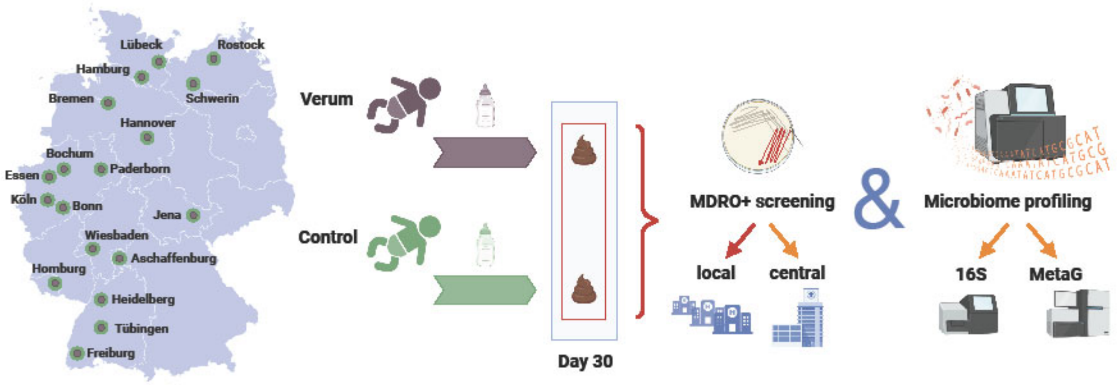

Preterm infants from 18 German study centers were randomized into two groups: probiotic supplementation (verum) and placebo (control) for 28 days. The primary endpoint (red arrow) was local multidrug-resistant organisms or highly epidemic bacteria (MDRO+) screening of stool samples on day 30 of life. Secondary endpoints (orange arrows) included MDRO+ screening at one central facility and microbiome profiling< using 16S rRNA gene and metagenomic (metaG) sequencing. Created with BioRender.com.

© 2024 Van Rossum T et al. *JAMA Pediatrics.*

eFigure 2: Model of eubiosis based on published metagenomes

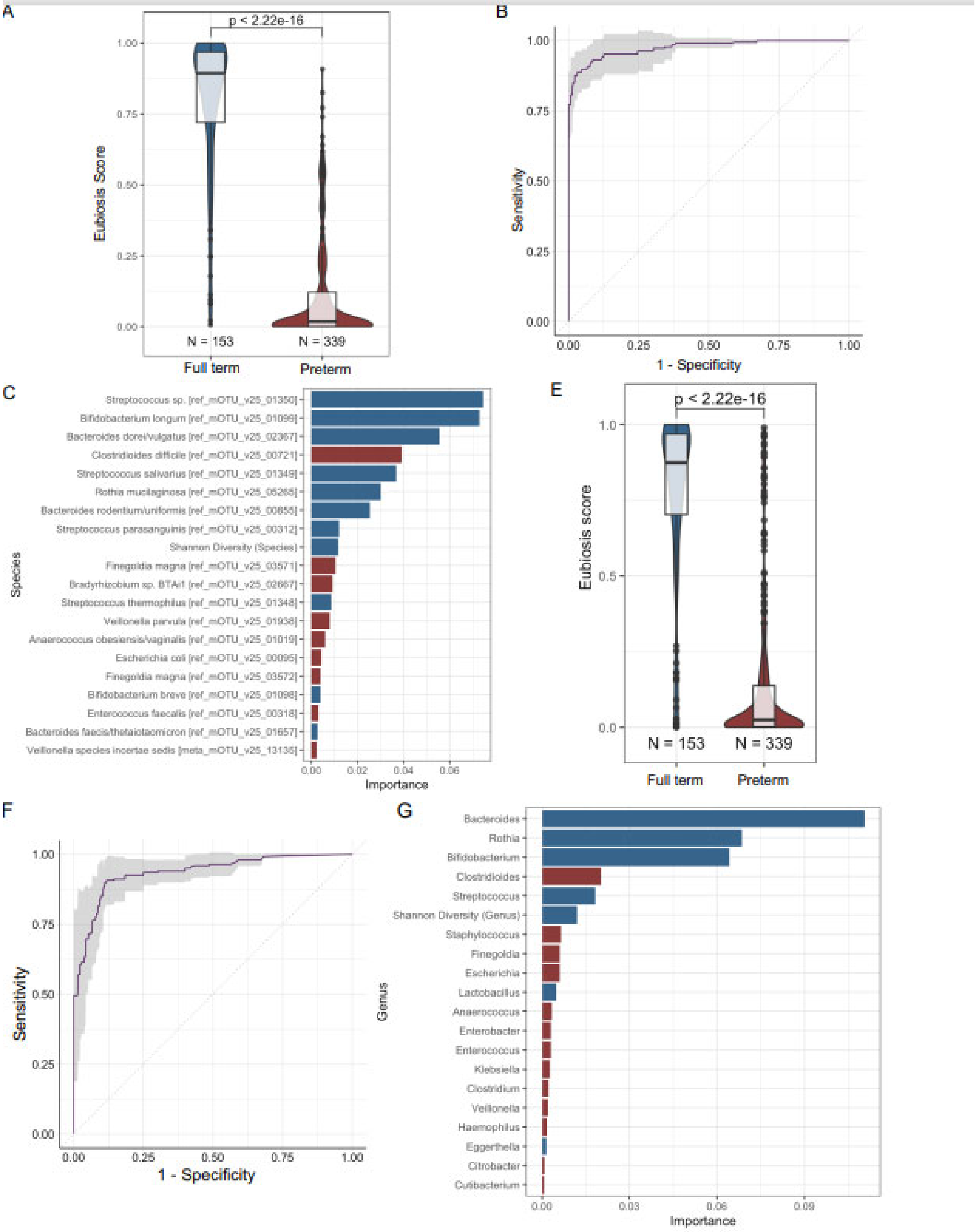

(A) eubiosis scores from species model cross validation results; (B) Receiver operating characteristic curve for species-based classification model of preterm versus full-term infants; (C) Most important taxa for the model predictions and the modeling group that they are more abundant in (blue for full-term, red for preterm); (D-F) Same as A, B, and C but for the genus-based model.

© 2024 Van Rossum T et al. *JAMA Pediatrics.*

eFigure 3. Enrollment, randomization and ITT population of the interim analysis

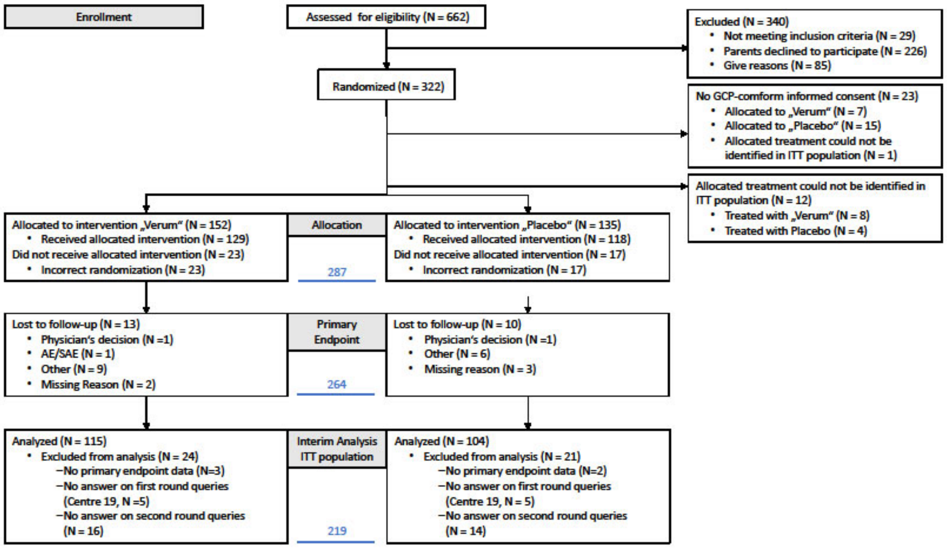

Abbrevation: ITT Itention To Treat population

© 2024 Van Rossum T et al. *JAMA Pediatrics.*

eFigure 4. Central MDRO+ assessment of Day30 samples according to intervention

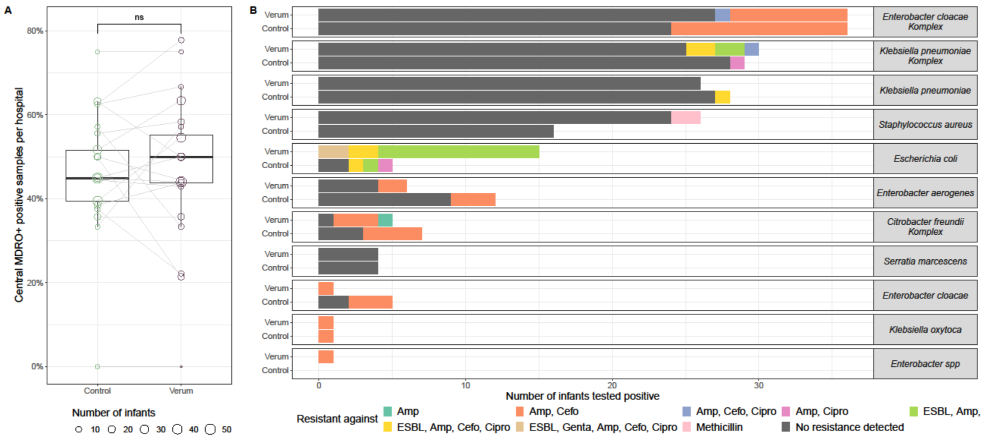

Central MDRO+ assessment of Day 30 fecal samples in as-treated population: (A) Prevalence of MDRO+ tested samples per study site, size of circles indicates the group-wise number of infants per study site. Grey lines connect the same study site between groups; (B) Distribution of detected MDRO+ species in conventional culture between groups. Colors indicate the presence of antibiotic resistance;

Abbrevations: MDRO+ Multidrug-Resistant Organisms or highly epidemic bacteria, Amp Ampicillin, Cefo Cefotaxim, Cipro Ciprofloxacin, ESBL Extended-Spectrum Beta-Lactamases, Genta Gentamicin.

© 2024 Van Rossum T et al. *JAMA Pediatrics.*

eFigure 5: Environmental uptake of probiotic strains

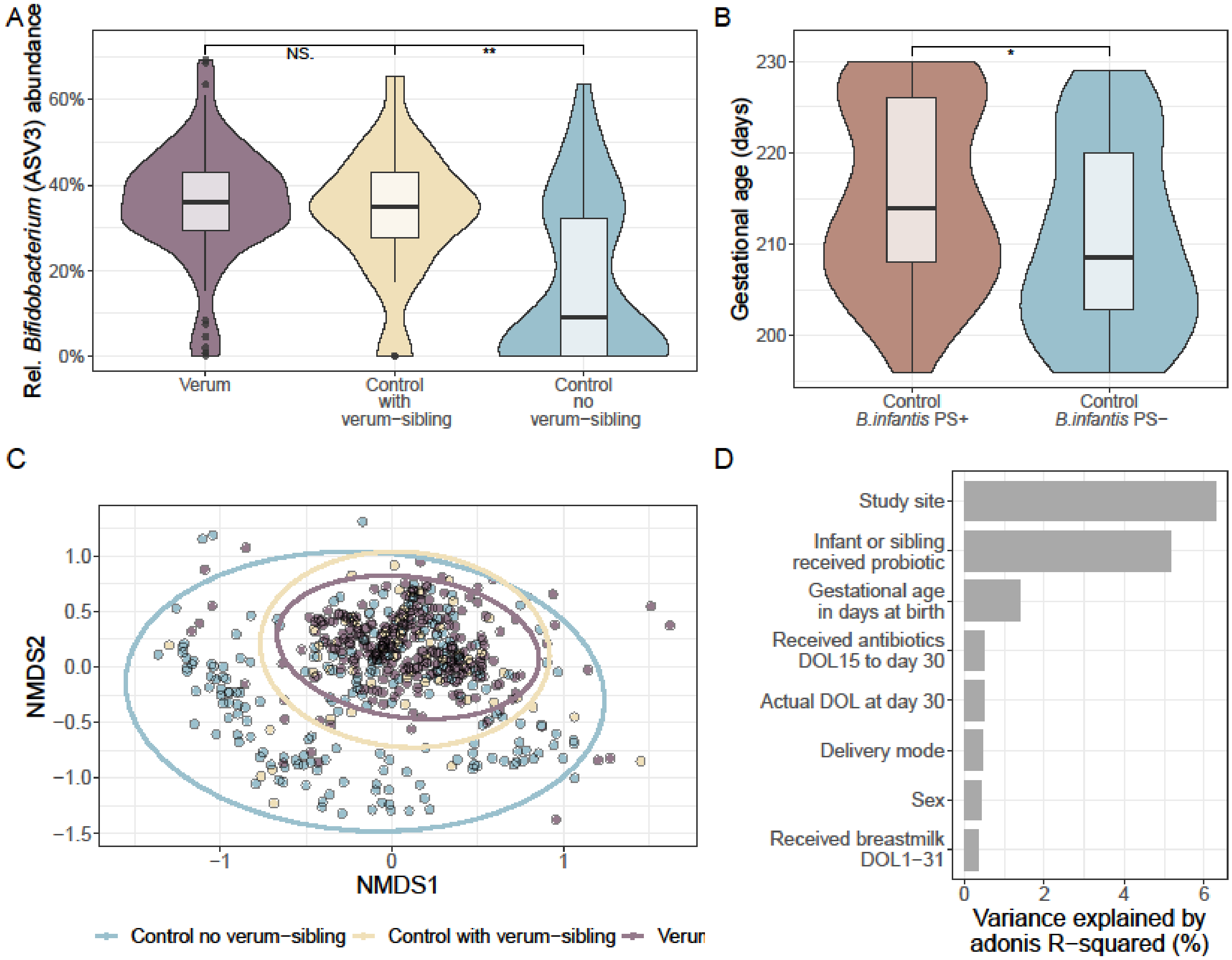

A) Relative abundance of one sub-genus group of *Bifidobacterium* (one amplicon sequence variants ASV ("ASV3")) as detected in the 16S sequenced samples, comparing verum infants, control infants with a sibling receiving the probiotic (Control with verum-sibling), and control infants without a sibling receiving the probiotic (Control no verum-sibling); B) Comparison of the gestational age in days at birth between the control group infants with *B. infantis* PS detected (Control *B. infantis* PS+) and those where it was not detected (Control *B. infantis* PS-); C) NMDS plot visualizing beta diversity at the ASV level for microbial communities for Day 30 samples in the 16S data set. NMDS axes capture Bray-Curtis dissimilarities between samples. Colors and ellipses highlight data dispersion within groups: Purple = verum infants, yellow = control infants with a sibling receiving the probiotic (Control with verum-sibling), blue = control infants without a sibling receiving the probiotic (Control no verum-sibling). Close overlap between verum and control with verum-sibling samples is observed; D) Results of Permanova analysis, where each bar represents the percentage of significant variance explained by adonis R2 for a metadata variable.Wilcoxon test * $p<0.05$, ** $p<0.01$; Abbrevations: PS Probiotic Strain, NMDS Non-metric MultiDimensional Scaling, DOL Day Of Life.

© 2024 Van Rossum T et al. *JAMA Pediatrics.*

eFigure 6: Microbiome variability and impact of verum treatment

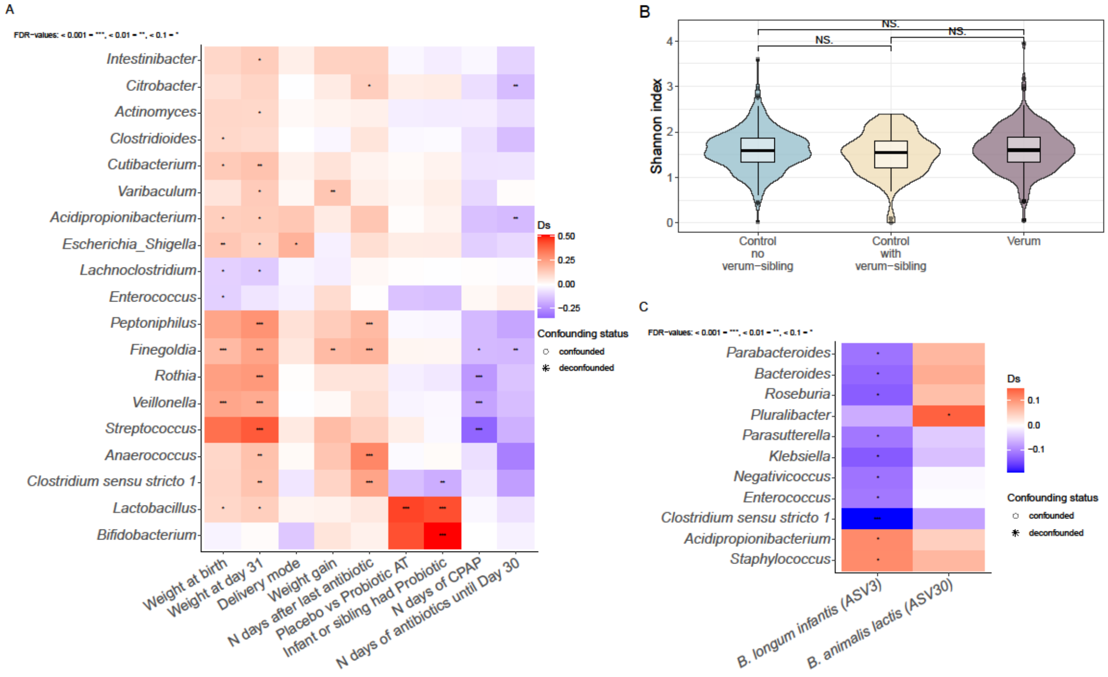

A) Genera significantly correlated with the metadata variables shown at the horizontal axis. Heatmap hues show Cliff's delta signed effect sizes, asterisk indicating post-hoc univariate significance FDR-Values: ***=<0.001, **=<0.01, *=<0.05; B) No significant difference in Shannon diversity index, comparing verum infants, control infants with a sibling receiving the probiotic (Control with verum-sibling), and control infants without a sibling receiving the probiotic (Control no verum-sibling); C) Genera significantly correlated with the ASVs closest matching the probiotic strains. Heatmap hues show Cliff's delta signed effect sizes, asterisk indicating post-hoc univariate significance FDR-Values: ***=<0.001, **=<0.01, *=<0.1.

© 2024 Van Rossum T et al. *JAMA Pediatrics.*

eAppendix 1. Study Efficacy Outcomes

The primary efficacy endpoint was gut dysbiosis at Day 30 of life defined as colonization with MDRO+ based on local microbiological screening. Infants were assigned to have MDRO+ colonization if, in the on-site surveillance according to local standards, one of the following organisms was detected in fresh stool cultures: (1) multidrug-resistant organisms; i.e. gram-negative bacteria with resistance to piperacillin and cefotaxim or ciprofloxacin or meropenem; methicillin-resistant *Staphylococcus aureus* and/or (2) pathogens without resistance characteristics but highly epidemic potential for outbreaks (*Serratia spp; Pseudomonas spp; Klebsiella spp.; Enterobacter spp., Acinetobacter spp)*.[1]

Key clinical safety outcomes are described in detail in the study protocol (**Supplement 1**, chapter 2.10). With regard to the potential risk for probiotic sepsis, all study sites received standard operating procedures to use blood culture bottles as per site standards (BacT/ALERT® PF Plus, bioMérieux Inc., USA). In the case of growth of *Bifidobacterium* spp.or *Lactobacillus acidophilus* the isolate should be recovered and cultured under anaerobic conditions on de Man-Rogosa-Sharpe (Oxoid) medium supplemented with 0.5 g/L cysteine-HCL for 48h, shipped to Mainz and further subjected to whole genome PCR as outlined below.

Secondary efficacy outcome measures included predefined components of gut dysbiosis at Day 30 of life based on (1) central MDRO+ screening and (2) microbiome composition analyses derived from 16S rRNA gene (16S data) and deep metagenomic sequencing (metaG data), and deviation from the microbiome pattern of term, vaginally born, antibiotic-free and fully breastfed neonates, which was defined as "eubiosis", on genus- and species level (eubiosis scores, see below).

eAppendix 2. Statistical Analysis and biostatistics for microbiome analysis

The statistical analysis are described in detail in **Supplement 1**, chapter 2.14. We additionally adjusted for „exposure units" (proximity to a verum treated infant) as a proxy to potential environmental uptake within the reality of performing a multicenter clinical trial. These exposure units were defined as exposure days per verum room neighbor in the NICU.

The biostatistical analysis of microbiome composition was conducted on the 16S dataset using the R environment Version 4.1. To remove spurious taxa, a prevalence threshold of 0.9% (each ASV analyzed present in at least 5 out of 538 samples) was applied as a filter before all subsequent analyses. Samples were excluded from the analysis if they were missing the tested metadata.

We used Non-Metric Dimensional Scaling (NMDS) for multivariate analysis with Bray–Curtis dissimilarities between samples. To assess the impact of various factors on microbiome variability, permutational analysis of variance (PERMANOVA) on distance matrices (Bray-Curtis dissimilarities) was performed in the vegan package (Version 2.6-4), utilizing the adonis2 function. Bray-Curtis dissimilarities were calculated based on relative abundances using the weighted distance function within the phyloseq package Version 1.38.0. The factors tested are listed in **eTable 3**. Furthermore, alpha-diversity measures (Shannon index, observed richness, Simpson index) based on untransformed counts were calculated using the phyloseq package. Differences between groups were compared using linear mixed-effect models. Alpha-diversity measures were considered as dependent variables, while AT-group or sibling group were treated as fixed effects, and sex, study site, and gestational age were treated as random effects.

e**Appendix 3. Microbiome composition analysis**

16S rRNA gene sequencing and deep metagenomic sequencing (metaG data) was performed as described in the study protocol (**Supplement 1,** chapter 2.9). Stool samples at Day 3 timepoint were valid if the sample was collected before or at most one day after the probiotic or placebo treatment began. Regarding 16S rRNA analysis, amplicon sequence variants (ASVs) were taxonomically annotated using the SILVA database V.138. Samples were discarded from further analysis if they contained less than 500 bacterial reads.

MetaG sequencing was conducted on a subset of Day 3 and Day 30 samples. After human host read removal, quality control (QC) assessment, and filtering according to the microbiome inclusion criteria described in **Supplement 1**, 209 samples were deemed suitable for analysis. These included 21 samples from the Day 3 time point and 188 samples from the Day 30 timepoint.

Species profiles were compiled using mOTUs v2.5. Strain-resolved detection results for the probiotic treatment strains (**eTable 1**) were generated using the metagenomic single nucleotide variant (metaSNV)-based ProTection[2] tool and SameStr[3]. For ProTection, the probiotic *B. infantis* strain was detected using genome-specific SNVs (for details, see **Supplement 1**). The probiotic strain was considered present if the reference genome had at least 99% horizontal and 8x mean vertical coverage, and all six of the strain-distinctive SNVs were present (**eTable 6**). If the genome coverage criterium was met but only one to five of the strain-distinctive SNVs were present, then the probiotic strain was classified as "likely present". These four uncertain cases were omitted from analysis and are listed as NA in the results table. In all other cases, the probiotic strain was considered "not detected".

eAppendix 4. Eubiosis score modeling

A model of normative eubiosis was created to contrast the fecal microbiome of dysbiotic preterm infants, untreated with probiotics, with that of eubiotic full-term, vaginally delivered, healthy infants. All samples were collected from infants at a similar age (3 to 12 weeks old). A random forest model (R package randomForest version 4.7 with 1000 trees, mtry of 100) was trained on taxonomic profiles generated from previously published metagenomes to distinguish between "preterm" microbiomes (339 metagenomes from 5 studies) and "full-term" microbiomes (153 metagenomes from 7 studies, **eTable 8**).

eAppendix 5. EMMA full-term cohort

For further evaluation of eubiosis, we enrolled 99 full-term neonates at the time of birth at the PRIMAL study center at the University of Lübeck Hospital. The institutional board of the University of Lübeck approved the EMMA study and written informed consent was given before enrollment. All infants were without perinatal complications and exclusively breastfed until Day 30 of life. Fecal sampling at Day 30 and Day 90 of life and all further processing steps (e.g. MDRO+ tests, 16S rRNA gene sequencing, metagenomic sequencing, and eubiosis score calculation) were conducted adherent to the PRIMAL study protocol and methods described above.

eAppendix 6. Environmental uptake of probiotic strains

We noted a higher abundance of the sub-genus group that contains the *B. infantis* probiotic ("Bifidobacterium ASV3") in the verum and 'control with verum-sibling' groups (**eFigure 5A**) and a correlation of probiotic uptake with later gestational age (**B**). Notably, control infants without a verum sibling had the most diverse range in their microbiome compositions (**C**). The adjusted analysis of the 16S data revealed that 5% of the microbiome variability is explained by the exposure to probiotics (verum group or verum-treated sibling) and 6% by study site, whereas gestational age, delivery mode and feeding human milk had a lower impact (<2%, **D**).

eAppendix 7. Bifidobacterium relative abundance

The most robust observation concerning *Bifidobacterium* relative abundance was the positive correlation with whether the infant or the infant's sibling had received a probiotic. Additionally, this probiotic intake was found to be positively associated with an increase in *Lactobacillus* and a decrease in *Clostridium sensu stricto* 1 relative abundance, respectively. Further, we observed that a set of genera (*Streptococcus, Veillonella, Rothia, Finegoldia, Peptoniphilus*) was increased in relative abundance in infants with higher weight at Day 30. Interestingly an opposing pattern was associated with the number of CPAP treatment days and the number of antibiotic treatment days at Day 30. The number of CPAP treatment days and the number of antibiotic treatment days at Day 30 negatively correlated with the weight of the infant, thus the described set of genera most likely describes the gut microbiome of older and in general healthier preterm infants. Infants born vaginally displayed a higher relative abundance of *Escherichia/Shigella*, however, the MDRO+ prevalence was not modified by birth mode (OR 1.16, 95% CI: 0.7-1.91) in a simple regression model with gestational age and sex.

eReferences

1 Härtel C, Faust K, Fortmann I et al. Sepsis Related Mortality of Extremely Low Gestational Age Newborns after the Introduction of Colonization Screening for Multi-Drug Resistant Organisms." *Antimicrobial Resistance and Infection Control* 2020; 9 (1): 144. https://doi.org/10.1186/s13756-020-00804-8.

2 Van Rossum T, Costea PI, Paoli L, et al. metaSNV v2: detection of SNVs and subspecies in prokaryotic metagenomes. *Bioinformatics.* 2022;38(4):1162–1164. https://doi.org/10.1093/bioinformatics/btab789.

3 Podlesny D, Arze C, Dörner E, et al. 2022. Metagenomic strain detection with SameStr: identification of a persisting core gut microbiota transferable by fecal transplantation." *Microbiome*. 2022; 10(1):53. https://doi.org/10.1186/s40168-022-01251-w.

4 Henrick BM, Rodriguez L, Lakshmikanth T, et al. Bifidobacteria-mediated immune system imprinting early in life." *Cell.* 2021;184(15):3884-3898.e11. doi: 10.1016/j.cell.2021.05.030.

© 2024 Van Rossum T et al. *JAMA Pediatrics.*
