# Supplementary material for: Bifidobacterium and Lactobacillus Probiotics and Gut Dysbiosis in Preterm Infants: The PRIMAL Randomized Clinical Trial
Source: JAMA Pediatr. 2024 Aug 5;178(10):985–95. doi: 10.1001/jamapediatrics.2024.2626 (PMC12549143; doi:10.1001/jamapediatrics.2024.2626)
Supplement: Supplement 4. — Data Sharing Statement. [file jamapediatr-e242626-s004.pdf]

Data Sharing Statement

Van Rossum. Bifidobacterium and Lactobacillus Probiotics and Gut Dysbiosis in Preterm Infants. *JAMA Pediatr*. Published August 05, 2024. doi:10.1001/jamapediatrics.2024.2626

Data
Data available: Yes
Data types: Deidentified participant data
How to access data: We will provide complete URL for data being available in a repository and data can also be requested at haertel_c1@ukw.de
When available: With publication

Supporting Documents
Document types: Statistical/analytic code
How to access documents: We will provide complete URL for data being available in a repository and data can also be requested at haertel_c1@ukw.de
When available: With publication

Additional Information
Who can access the data: Researchers whose proposed use of the data has been approved
Types of analyses: For any puropse that is inline with the content of informed consent
Mechanisms of data availability: after approval of a proposal and with a signed data access agreement
Any additional restrictions: none
